# Designing metaverse interaction systems for the Turkish language enhanced by fine-tuning and retrieval-augmented generation (RAG)

**DOI:** 10.1038/s41598-026-35392-x

**Published:** 2026-04-20

**Authors:** İbrahim ÖZKAL, Fatih BAŞÇİFTÇİ

**Affiliations:** Department of Computer Engineering, Faculty of Technology, Selcuk University, Konya, Turkey

**Keywords:** Metaverse, Natural language processing, Large language models, Retrieval-augmented generation, Fine-tuning, Non-Player character, Engineering, Mathematics and computing

## Abstract

The metaverse refers to a digital environment that enables real-time user interaction through immersive technologies. Recent advancements in deep learning have particularly strengthened the capabilities of Natural Language Processing (NLP) and Large Language Models (LLMs). These developments have made human–computer interactions in the metaverse more natural and responsive, especially those involving users and non-Player Characters (NPCs). Many virtual platforms use LLMs-powered Application Programming Interfaces (APIs) to facilitate these interactions, but these often produce long, semantically irrelevant responses that weaken the user’s immersive experience. This study addresses this limitation by designing NLP systems capable of generating concise, context-aware, and task-oriented outputs for AI-powered NPCs. Unlike open-domain conversational agents, dialogue systems for metaverse-based NPCs operate under strict real-time and contextual constraints. NPC interactions require concise, task-oriented, and context-aware responses, as overly long or semantically irrelevant outputs can disrupt immersion and degrade user experience. Although recent advances in LLMs have improved dialogue generation, most existing studies focus on open-ended conversations or general-purpose question answering. This study addresses this gap by systematically investigating fine-tuning and Retrieval-Augmented Generation (RAG) strategies within a metaverse-focused dialogue domain. We propose a comparative evaluation of systems developed using fine-tuning and RAG techniques between decoder-only models (GPT-2, LLaMA, Qwen) and encoder-decoder models (mBART, mT5). The models trained on the dataset were evaluated using a combination of standard evaluation metrics and semantic-based criteria. All evaluation scores were normalized using the Technique for Order Preference by Similarity to Ideal Solution (TOPSIS) method to ensure objective comparability between models. The findings indicate that RAG provides a more balanced performance, particularly when applied to encoder-decoder models such as mBART (~ 0.652) and mT5 (~ 0.555), even when trained on relatively small datasets. Additionally, this paper presents a speech-based interaction framework designed to enable personalized and real-time communication in metaverse environments. The proposed framework is structured as Speech-to-Text (STT) → LLMs → Text-to-Speech (TTS). This architecture improves interaction quality by enabling coherent and realistic speech-based communication.

**Supplementary Information:**

The online version contains supplementary material available at 10.1038/s41598-026-35392-x.

## Introduction

The digital age continues to expand the boundaries of technology, bringing forth numerous innovations. One of the most notable innovations in this digital process is the concept of the metaverse^12^. The metaverse is broadly defined as a virtual environment where individuals interact through avatars. It integrates virtual and physical worlds to provide users with real-time, interactive, and immersive experiences^81^. This virtual space is not limited to socializing and entertainment; it also paves the way for innovative applications across various sectors such as education, commerce, and healthcare. In particular, its ability to make users’ personal and professional lives more interactive and accessible in the virtual realm further enhances the potential of the metaverse. However, despite all this potential, the creation of realistic and dynamic virtual environments remains a significant challenge. Ensuring the sustainable functioning of these environments is another issue that has not yet been fully addressed.

Advances in AI technologies will enable the development of more empathetic avatars and applications[^4^]. In this context, AI technologies are expected to facilitate more effective user interactions by learning from user behavior and continuously updating this information. By analyzing user behavior, making learning-based decisions, and adapting to environmental changes, AI systems significantly enhance the dynamism and personalization of metaverse experiences^36^. In this context, interactions between AI-powered characters and users are among the key elements that enhance the sense of realism in digital environments^57^.

One of the key application areas of AI-based systems in metaverse environments is the interaction between users and AI-powered artificial characters, known as NPCs. Interactions between NPCs and users constitute a significant application area of AI in the metaverse. In this context, AI-powered NPCs have emerged as active participants capable of engaging in natural and meaningful communication with humans. One of the main tools of this interaction is voice communication^6^.

In metaverse and VR settings, sound helps users feel more present and makes interactions more engaging. Thanks to speech processing technologies, AI-powered NPCs can engage in natural, fluent, and meaningful communication with users, as if interacting in the real world. These characters engage in dialogue with users, perform tasks, and actively participate in interaction processes. For interactions to be effective, dialogues between users and Artificial Intelligence supported non-Player Characters (AI-NPCs) should be designed to be concise, context-aware, and task-oriented. Recently, NLP approaches have become increasingly important in the development of such systems.

Since the 2000s, NLP technologies have advanced rapidly. With the integration of LLMs into daily life in the 2020s, human–AI interaction has significantly improved. NLP is a subfield of AI that encompasses various computational models and learning processes aimed at automatically analyzing and understanding human language, including both speech and text^58^. It focuses on the ability to process and analyze linguistic data effectively. LLMs are massive deep learning models that are pre-trained on vast amounts of data. The advancement of NLP and LLMs technologies has improved the ability of AI systems to communicate in a more human-like way. These technologies support tasks such as text generation, language understanding, sentiment analysis, and multimodal interaction. This situation presents new opportunities for human-centric AI design, especially in highly interactive digital environments such as the metaverse.

Despite the significant advancements in AI technologies, AI-NPCs still encounter various technical limitations and challenges in accurately interpreting user commands and generating appropriate responses^37^. One of the key technical challenges in metaverse and virtual environments is the accurate interpretation of user inputs. Many virtual platforms rely on APIs to facilitate such interactions; however, these often result in lengthy and semantically irrelevant responses, which can diminish the user’s immersive experience. Enabling AI-NPCs to accurately understand user requests in context and enhancing their communication abilities are among the fundamental requirements in this field. Research indicates that NPCs have the potential to provide more immersive and interactive experiences in metaverse environments (Armand et al., 2023; Lukaj et al., 2023^60^). Although AI-powered NPCs are expected to generate context-aware and task-oriented responses, their reliance on APIs may lead to the production of irrelevant or unnecessary information. This negatively affects the user experience by reducing the quality and realism of the interaction. To enhance communication with AI-NPCs in the metaverse, there is a need to optimize LLMs systems and develop specialized AI models capable of generating task-oriented responses only.

Unlike open-domain conversational agents, dialogue systems designed for metaverse-based NPCs operate under strict real-time, immersion-sensitive, and context-dependent constraints. NPC interactions are expected to produce concise, task-oriented, and context-aware responses, as overly verbose or semantically misaligned outputs can interrupt narrative flow and significantly degrade user immersion^69^. However, the majority of existing NLP and LLM-based dialogue studies predominantly focus on open-ended conversation, general-purpose question answering, or chatbot-oriented benchmarks, where response length and exploratory generation are often encouraged rather than constrained^77^. Consequently, the unique communication requirements of metaverse NPC dialogues (e.g., brevity, immediacy, and situational appropriateness) are not sufficiently addressed in the literature. This gap highlights the need for systematic investigation of dialogue generation strategies that are explicitly optimized for metaverse-oriented NPC interactions, where response quality is tightly coupled with real-time performance and experiential realism.

As of 2024, advancements in the field of LLMs have enhanced the effectiveness of techniques such as fine-tuning and Retrieval-Augmented Generation (RAG). These methods enable AI models to generate responses that are both contextually sensitive and up-to-date. The RAG technique is an approach that supports response generation by retrieving information from external data sources. It encompasses both the retrieval and generation processes. On the other hand, fine-tuning aims to improve task-oriented performance by retraining pre-trained LLMs on specialized tasks or datasets. In short, fine-tuning enhances a model’s sensitivity to specific content, while RAG strengthens contextual adequacy by supplementing the model with external information sources^7^.

This study examines LLMs used in AI-NPC characters that enhance the sense of realism in metaverse environments. A comparative analysis was conducted on LLMs whose contextual adequacy was improved through fine-tuning and RAG techniques. RAG provides contextual depth to LLMs through its ability to retrieve external information and integrate it into the model. In contrast, fine-tuning offers a more specialized approach by enhancing model performance for specific tasks or scenarios. By evaluating these two techniques across different model architectures, this study aims to identify the most effective strategy for generating coherent, concise, and contextually appropriate responses in AI-NPCs interactions. The main contributions of this paper are as follows:

- We investigate the most suitable techniques for enabling meaningful, concise, and task-oriented communication between users and AI-NPCs in metaverse environments. Our focus is on short, context-aware question–answer interactions that enhance the sense of realism.
- We implement fine-tuning and RAG theqniques on a custom-designed dataset. In doing so, we employ both decoder-only models (GPT-2, Qwen, LLaMA) and encoder-decoder models (mBART, mT5) to evaluate AI-NPC response generation mechanisms.
- We conduct a comprehensive performance comparison between the models developed using RAG and fine-tuning techniques, highlighting their respective advantages and limitations. To ensure fair comparability across different architectures and metrics, we normalize all evaluation scores using the TOPSIS method.
- To support real-time and personalized communication in virtual environments, we propose a speech-based interaction pipeline structured as STT → LLMs → TTS. This framework enables the transcription of user speech into text, the generation of semantically appropriate responses, and the presentation of outputs through synthesized speech.
- Finally, we present the advantages and limitations of the proposed architecture, offering a critical evaluation of the challenges encountered during the study. Based on these insights, we outline several recommendations to guide future research and further enhance AI-NPCs interaction frameworks in metaverse environments.

The rest of this paper is organized as follows: In the second section of the study, a historical overview of the field of NLP is provided to help readers grasp the foundational conceptual framework. Within this context, the core features of transformer architectures and general information about LLMs are presented. Additionally, Theoretical foundations of fine-tuning and RAG techniques are presented alongside practical examples to illustrate their application in virtual environments. The third section explores the use of LLMs through AI-NPCs in metaverse environments. The fourth section details the methodological framework of the study, including the datasets, model types, evaluation metrics, and steps of the experimental process. In the fifth section, the findings are systematically analyzed under three main themes, and the comparative performance of fine-tuning and RAG techniques is presented. The sixth section presents the overall conclusions, the seventh discusses the study’s limitations, and the final section offers suggestions for future research and references.

## Related work

This section provides readers with an overview of the rapid development of LLMs from past to present, along with fundamental information about the specific LLMs used in the study. Brief descriptions of the relevant techniques are also included to support understanding of the applicability of the models. The content is organized chronologically, beginning with the evolution of NLP, followed by transformer architectures, LLMs structures, and the fine-tuning and RAG techniques. The section concludes with examples demonstrating how these techniques are applied in virtual environments.

### Natural language processing

**Fig. 1:**
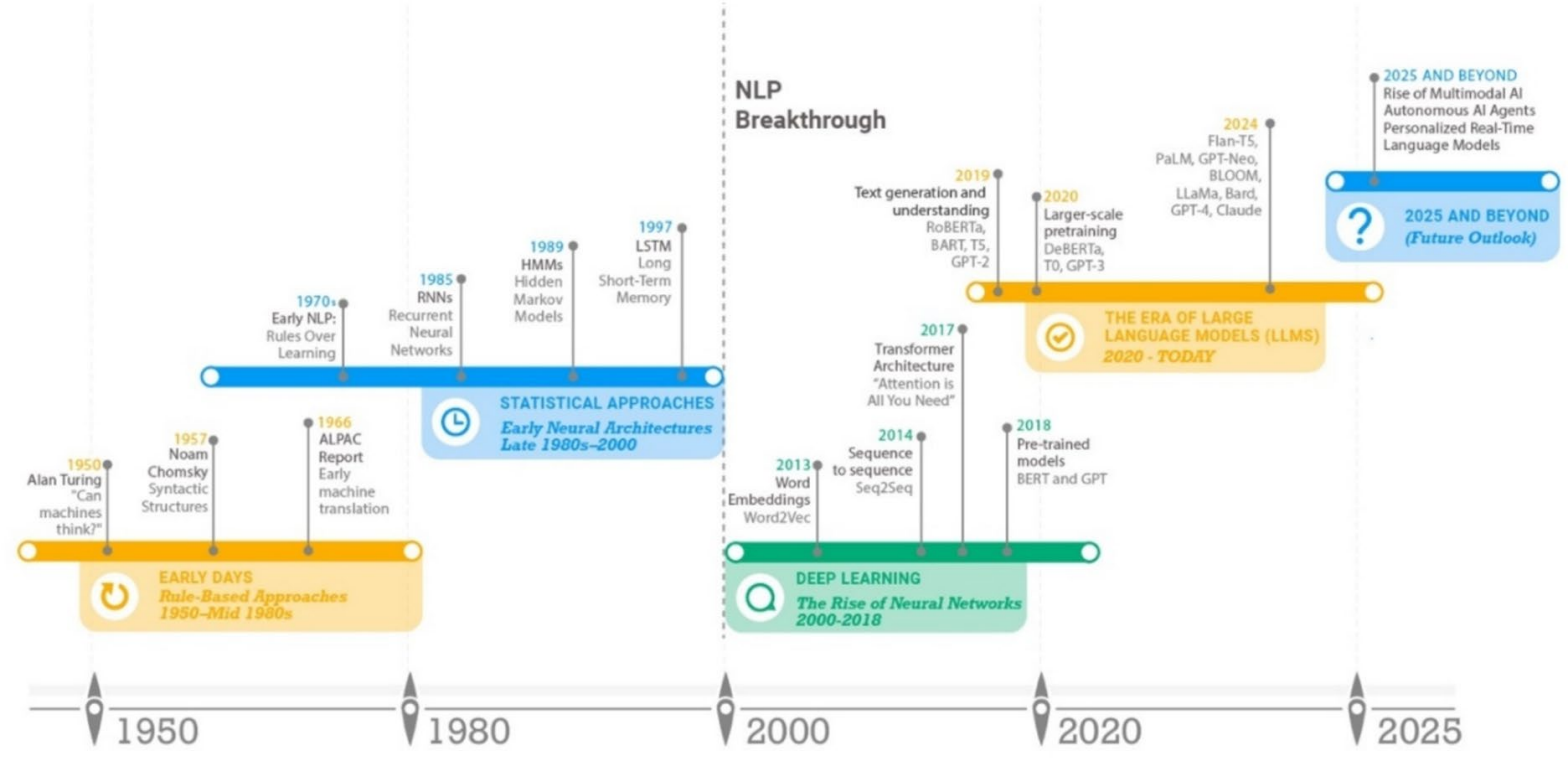
The historical development of NLP.

Advances in the field of NLP have laid the groundwork for the emergence of advanced language models such as LLMs. These models are trained on large-scale datasets and are capable of effectively learning both the structural and contextual features of language. Today, NLP serves as a bridge between human language and AI, focusing on the automatic analysis, comprehension, and generation of language by computers. In its broadest sense, NLP encompasses the process through which natural languages are processed and interpreted by computer systems^20^. NLP research began in the 1950s as a central focus of AI studies and has continuously evolved since then. It gained significant popularity starting in the mid-2010s^86^. The historical development and key stages of NLP are visualized in Fig. 1.

NLP is an interdisciplinary research field situated at the intersection of linguistics and computer science. Throughout its historical development, it has undergone significant transformations influenced by various paradigms. In the 1950s, Alan Turing’s famous question “Can machines think?” and the subsequent introduction of the Turing Test laid the theoretical foundation for both artificial intelligence and natural language processing^10^. Furthermore, Noam Chomsky’s 1957 publication Syntactic Structures introduced the theory of formal grammar, which significantly shaped NLP by promoting rule-based approaches to language modeling.

Traditional neural networks lack an architecture that accounts for the sequential nature of data. As a result, they faced several limitations when dealing with time-dependent or sequential inputs. To address this issue, approaches such as Recurrent Neural Network (RNN) and Hidden Markov Models were introduced in the late 1980s. Subsequently, the introduction of Long Short-Term Memory (LSTM) in 1997 marked a significant advancement in language processing by enabling more effective handling of sequential data^14, 24^. These network architectures align structurally with the natural flow of human language and have achieved high success in text-based applications. In particular, the transformer architecture proposed in 2017 with the paper *“Attention is All You Need”* initiated a revolutionary shift in NLP. By leveraging attention mechanisms, this architecture significantly improved the performance of deep learning-based language models through its ability to process long-range dependencies more effectively.

### Transformer models

**Fig. 2:**
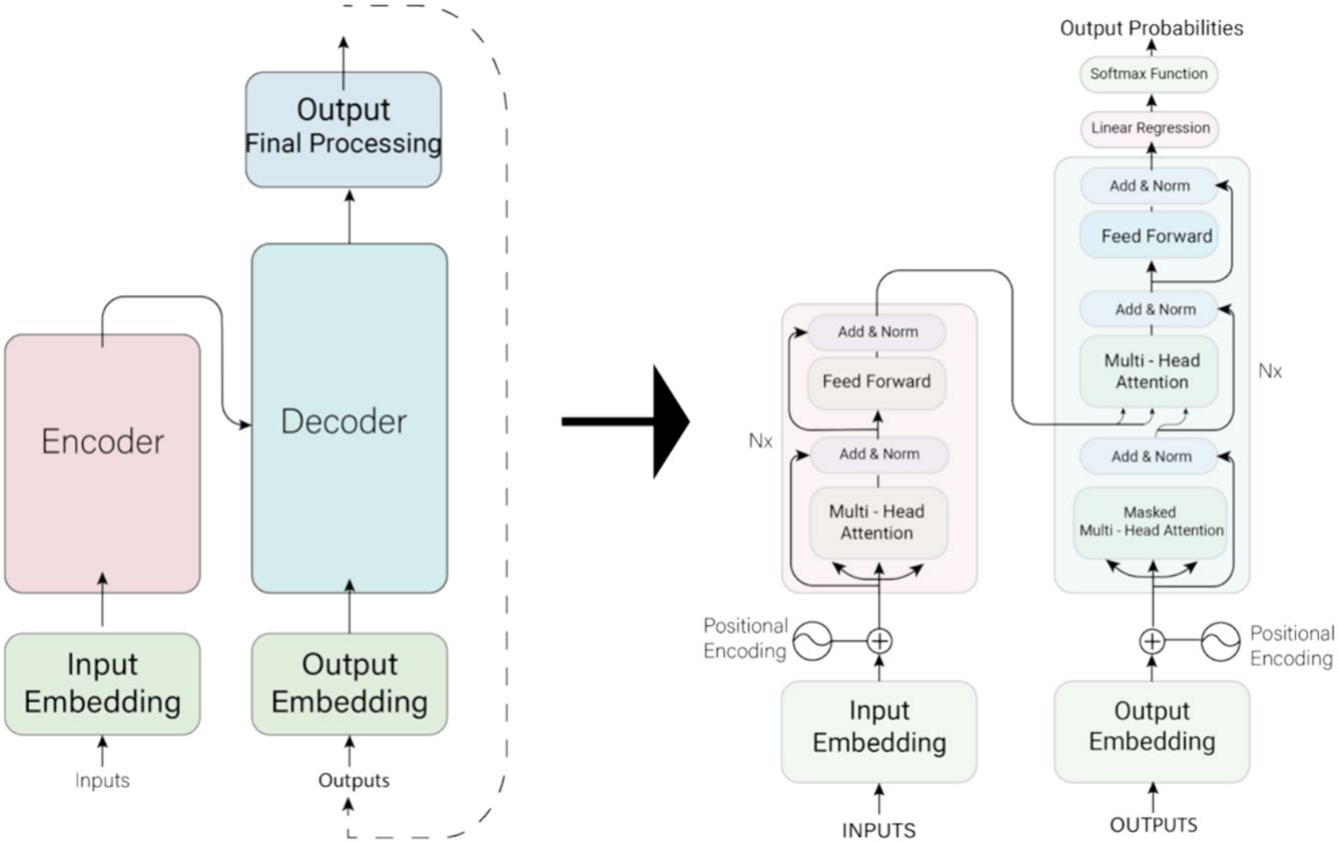
General structure of transformer models.

Transformer models, introduced in 2017, were developed to address the limitations of traditional sequential architectures such as RNN and LSTM. The most distinctive feature of this new architecture is its reliance on an attention mechanism that considers all input data simultaneously, rather than processing it sequentially. This structure enables more effective learning of long-range dependencies and significantly improves computational efficiency during the modeling process^53^. Transformer models are widely used in the field of NLP, particularly for tasks involving sequential data processing such as language translation [^74^]. This architecture generally consists of two main components: an encoder and a decoder. Depending on the nature of the task, the model architecture can be configured as encoder-only, decoder-only, or a combined encoder-decoder structure. The general structure of transformer architectures is presented in Fig. 2.

As shown in Fig. 2, transformer models demonstrate high performance in NLP tasks such as text translation and summarization thanks to their attention-based architecture. This architecture consists of two main components: an encoder and a decoder. The input data is first processed through embedding and positional encoding steps and then passed through multi-layered structures for further computation. The decoder generates new outputs by processing the information received from the encoder along with the previous outputs. These outputs are then transformed into a probability distribution using the softmax function. Introduced in 2017, the transformer architecture marked a significant advancement in NLP and contributed to the development of LLMs after 2018.

### Large language models

The development of the transformer architecture significantly improved the efficiency of model architectures by allowing for parallel computation during training. This advancement addressed the limitations of sequential processing in earlier models, such as RNN and LSTM. It also allowed LLMs to be trained more efficiently on large-scale datasets, leading to faster convergence, improved scalability, and enhanced overall performance^18,31^. These models are trained on large-scale datasets to learn linguistic patterns and structural relationships within the data, enabling them to generate coherent and contextually meaningful content. These models are commonly known as GenAI. They form the foundation of modern conversational systems such as ChatGPT and Bard. GenAI systems generate human-like text primarily through mechanisms that predict the most probable next word. These models are effectively used in a variety of NLP tasks, including text completion, language translation, and text summarization.

As artificial neural networks have become increasingly prevalent in NLP, word embedding models have been widely adopted as general-purpose representations for word modeling^26^. These models generate static vector representations of words based on their context in unlabeled text. They are typically used for word prediction tasks based on dimensionally reduced co-occurrence matrices. Word embedding has improved the performance of neural networks by providing rich, self-supervised representations of words, even in tasks with limited data^85^. Specifically, LLMs are defined as models trained on over a billion words and capable of performing inference through transfer learning. Building on this foundation, transformer-based LLMs represent a significant advancement in the field of NLP. Trained on extensive text datasets and equipped with billions of parameters, these models are designed to understand and generate human-like language across a wide range of tasks. LLMs such as BERT, GPT, and PaLM not only capture complex linguistic patterns but also adapt to various use cases through transfer learning^46^. These models are architecturally based on attention mechanisms. They enable context-aware and coherent language generation, which is central to a wide range of applications—from content creation and translation to code generation and research assistance. These models not only understand text but are also capable of performing a wide range of tasks, including content generation, question answering, code writing, translation, and summarization.

### Fine-tuning process of LLMs

**Fig. 3:**
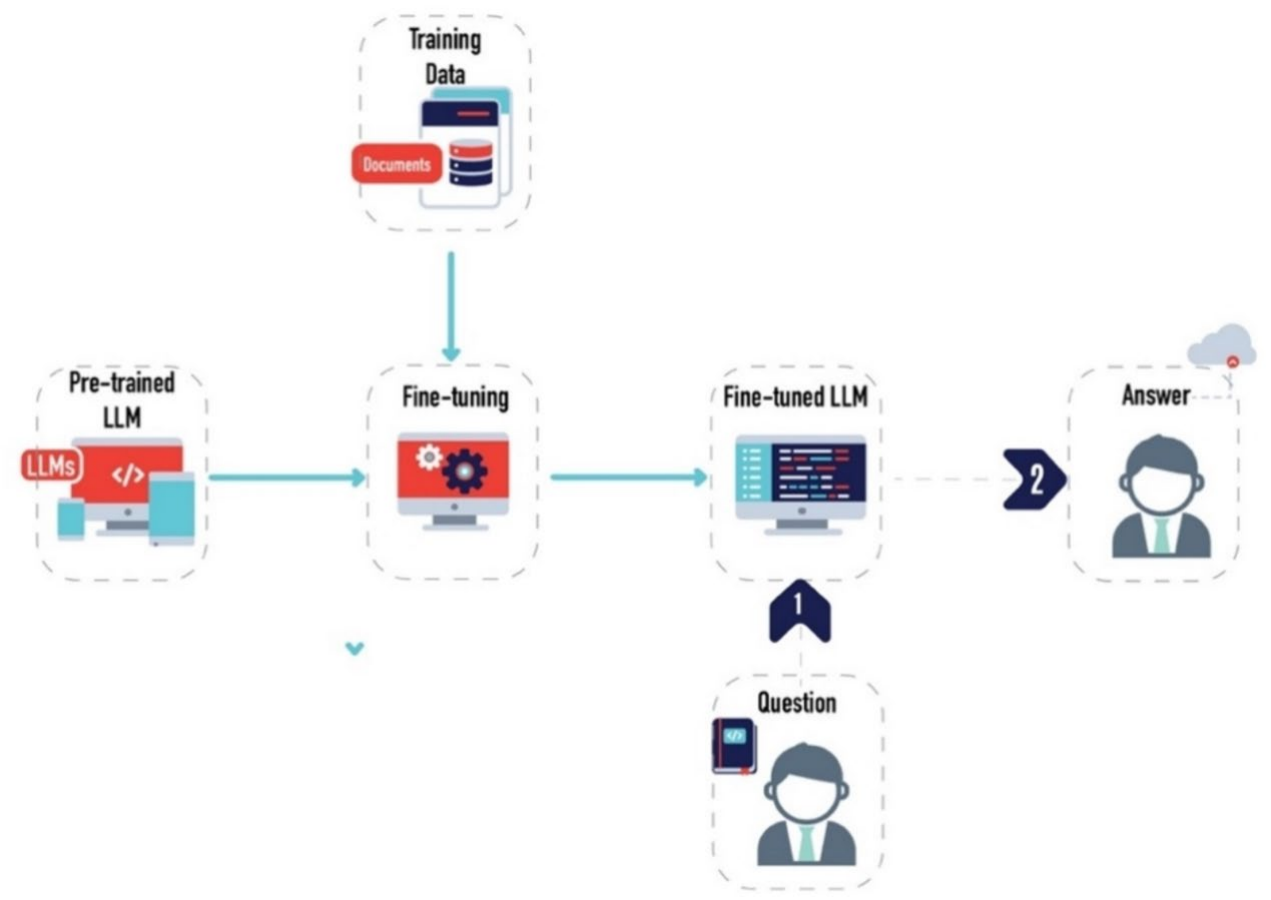
Example of the fine-tuning process.

LLMs are trained on large-scale datasets to learn the general structure of natural language^5^. However, to achieve high performance on specific tasks, these models often require fine-tuning with domain-specific data, enabling them to adapt their general capabilities to particular linguistic structures and use cases. Fine-tuning is the process of retraining a pre-trained model using a smaller, domain-specific dataset^73^. This process helps align the model more closely with a specific task or domain, enabling it to interpret concepts and terminology within the relevant context with greater accuracy. The overall architecture and workflow of the process are visualized in Fig. 3.

Figure 3 presents the fine-tuning process for LLMs. Before initiating this process, the data is prepared texts are cleaned, missing values are handled, and the content is formatted in a way that the model can understand. Next, a pre-trained model is carefully selected; this model must be structurally and contextually aligned with the target task. During the fine-tuning process, technical parameters such as learning rate, number of epochs, and batch size are configured appropriately. Typically, the early layers of the model are frozen while the later layers are fine-tuned, allowing the model to retain prior knowledge while adapting to the new task. During training, the cross-entropy loss function (1) is commonly used to measure the difference between the model’s outputs and the true labels.

1 $$\begin{array}{*{20}c} {L_{Fine - tuning} = - \mathop \sum \limits_{i = 1}^{N} y_{i} \log \left( {y^{\prime}_{i} } \right)} \\ \end{array}$$

This function expresses how accurately the model performs predictions in classification-based tasks. After training, the model’s performance is evaluated using metrics such as accuracy, loss, and precision. If necessary, the parameters are adjusted and reconfigured. In the final stage, the model is deployed into the real-world environment, ensuring that it operates securely, efficiently, and effectively. Through these steps, the model becomes optimized for the specific tasks.

### Retrieval-augmented generation process of LLMs

RAG is an AI approach that enables LLMs to generate more accurate and up-to-date responses by retrieving information from external data sources^49^. One of the primary motivations behind the development of this method was the limitations observed in accessing current data during retrieval processes. To address these shortcomings, the RAG architecture was introduced.

**Fig. 4:**
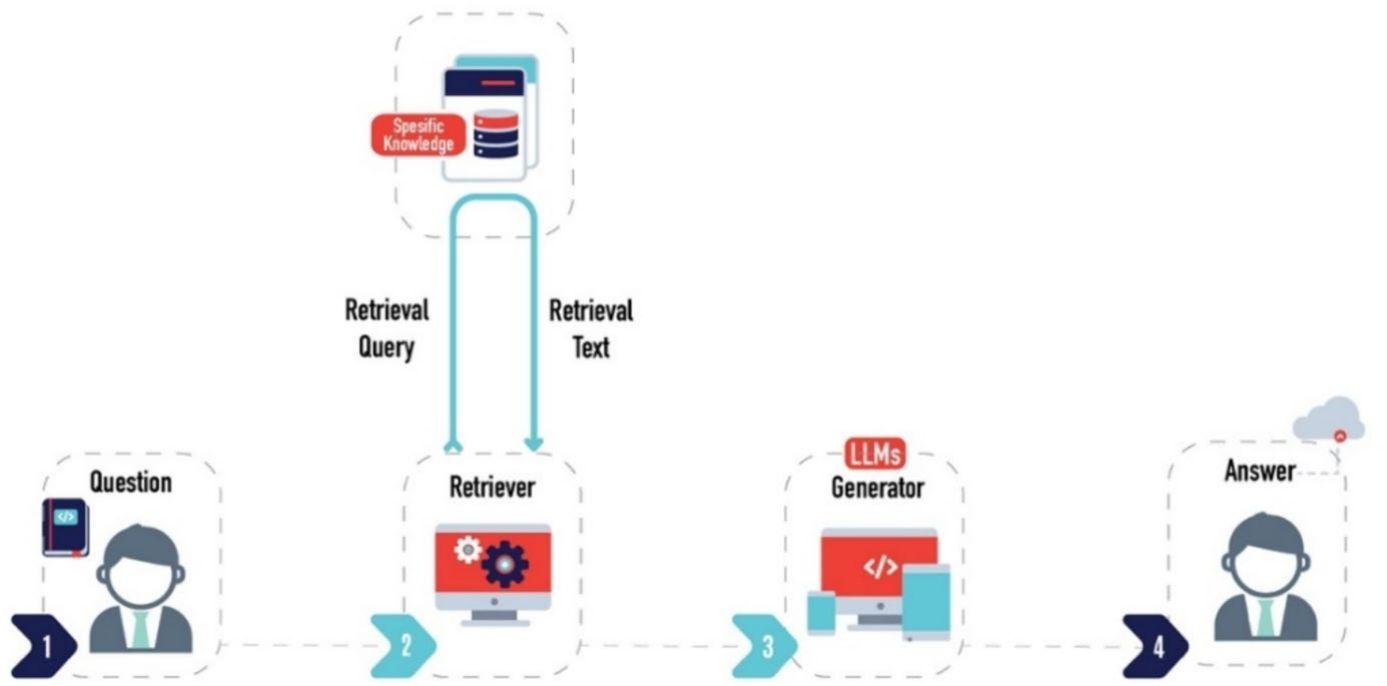
Example of the RAG process.

The RAG technique refines LLMs by integrating real-time information retrieval, allowing them to function independently of their original training datasets. This architecture provides immediate access to external knowledge sources in order to obtain up-to-date and contextually relevant information. Through its retrieval process from external databases or knowledge bases, the model enhances the accuracy and timeliness of the generated content. In this respect, RAG offers a significant advantage over traditional models that are limited by static datasets. The overall operational flow of the architecture is illustrated in Fig. 4.

The basic operation of the RAG process is illustrated in Fig. 4. The process begins when a user submits a query or command, which may aim to retrieve current information or generate creative content. The system’s first task is to understand and interpret this input. This initial interpretation determines what kind of information the system will search for. Once the user input is analyzed, the retriever component of the RAG system is activated. It finds content semantically related to the query by searching external data sources. Retrieval mechanisms such as Best Matching 25 (BM25), Contextualized Late Interaction over BERT (ColBERT), Facebook AI Similarity Search (FAISS) and Dense Passage Retriever (DPR) may be employed during this phase^9^[^44^]^47^. These sources can range from current news articles to academic papers. The aim is to identify information that is relevant to the user’s request. This search process relies not only on keyword matching but also on the semantic meaning of the query, thus yielding more accurate and contextually relevant results.

After the search phase, the system selects and compiles the most relevant content. This step forms the information set to be used in the generation phase. The quality of the retrieved data directly affects the accuracy of the response. For this reason, the system not only prioritizes relevance, but also reliability. This is a critical stage for delivering accurate and reliable information to the user^75^. In the fourth step of the RAG technique, the selected content is used to enrich the original user query. Contextual support is added to the initial input, making the query more precise and comprehensive. This enrichment allows the model to access up-to-date information that is not found in the original training data.

**Fig. 5:**
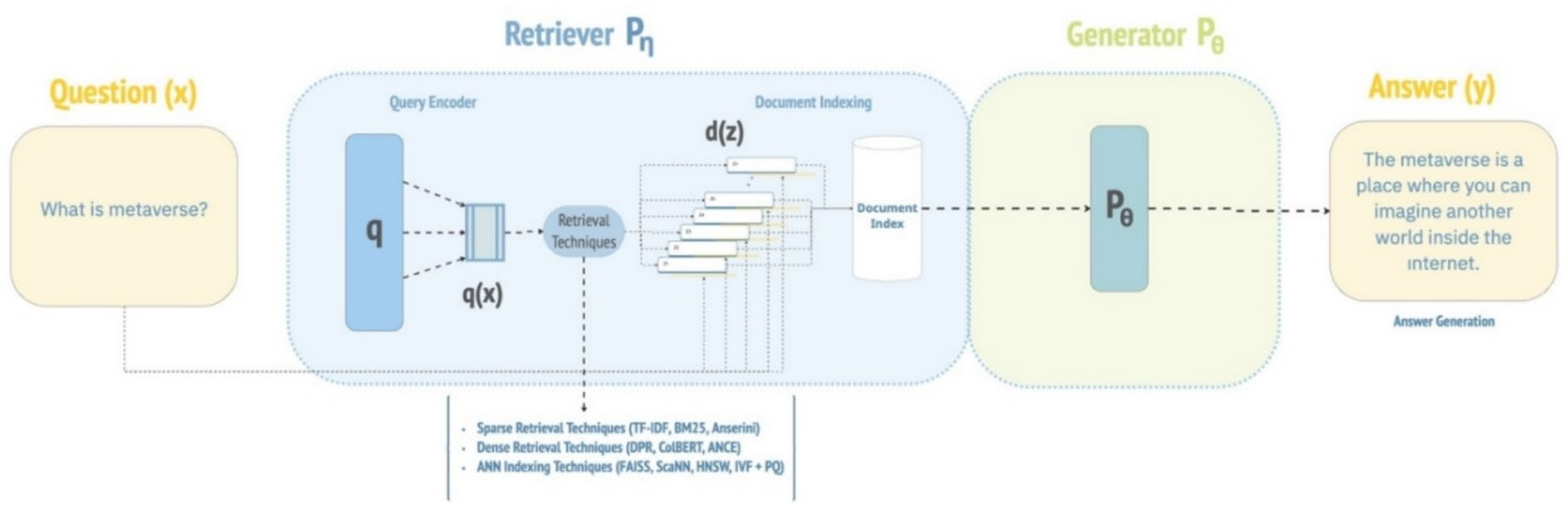
Mathematical explanation of the RAG process.

The enriched query is then sent to the LLMs. Since the model has been trained on a comprehensive corpus of text, it can better understand the context provided by the expanded input. Based on this, it generates a meaningful, consistent, and contextually appropriate response to the user’s query. In the final step, the generated output is communicated to the user. This response is fed not only by the model’s original training data but also by current and query-specific information. As a result, the system provides more accurate, up-to-date, and user-relevant output. Lewis et al.^38^, present the mathematical foundation of the RAG technique within the framework shown in Fig. 5.

Figure 5 visually illustrates how the retriever and generator components of the RAG technique function. The first three steps in the lower section of the figure show how the retriever selects relevant documents based on the user’s input and how this process is defined mathematically. In the last part of the figure, the generator component is shown, and again using mathematical expressions and the documents obtained, it is explained in detail how each output indicator is produced.

First, document-based sequential output generation is targeted. In RAGsequence, the model generates a response to a query by relying on a single document. That is, if a piece of text is to be produced, the same document is used from beginning to end (2).

2 $$\begin{array}{*{20}c} {P_{{RAG - Sequence \left( {y{|}x} \right)}} \approx \mathop \sum \limits_{{z \in top - k\left( {p\left( {.{|}x} \right)} \right)}} p_{n} \left( {z{|}x} \right) \mathop \prod \limits_{i - 1}^{N} p_{\theta } \left( {y_{i} {|}x,y_{1:i - 1} } \right)} \\ \end{array}$$

In this equation, *x* represents the input (e.g., a question), *y* denotes the output text to be generated (e.g., an answer), and *z* refers to the documents retrieved by the retriever. ${{p}_{n}}_{\left(z|x\right)}$ estimates the probability that document *z* is relevant to the input *x*. ${p}_{\theta }\left({y}_{i}|x,{y}_{1:i-1}\right)$ expresses the probability that the generator will produce token ${y}_{i}$ given document *z* and the preceding tokens.

In the second stage, the RAG-token model selects the most relevant document for each output word individually (3). That is, the first word may come from one document, while the second word may be retrieved from another. This enables the generation of more flexible and diverse content. At this stage, for each token ${y}_{i}$ the model considers* k* documents. The contributions from each document are weighted by their probabilities and then summed. This allows the selection and generation of tokens to become dynamic for every output word.

3 $$\begin{array}{*{20}c} {P_{{RAG - token \left( {y{|}x} \right)}} \approx \mathop \prod \limits_{i = 1}^{N} \mathop \sum \limits_{{z \in top - k\left( {p\left( {.{|}x} \right)} \right)}} p_{n} \left( {z{|}x} \right) p_{\theta } \left( {y_{i} {|}x,z, y_{1:i - 1} } \right)} \\ \end{array}$$

In the third stage, the retriever component is activated. The retriever is responsible for identifying documents relevant to the user input. To accomplish this, it represents both the documents and the query as vectors (4). At this stage, ${q}_{\left(x\right)}$ represents the vector representation of the query, while ${d}_{\left(z\right)}$ denotes the vector representation of a document. Similarity is measured using the inner product ${d}^{{\rm T}}{q}$ , and the most similar documents are selected accordingly. The generator combines the user input with the retrieved document to produce language output, similar to how LLMs operate. It is typically implemented as an encoder-decoder model and is particularly effective for sequence-to-sequence (seq2seq) tasks. The encoder receives *x* (the user query) and* z* (the document retrieved by the retriever) as input. The decoder then generates tokens sequentially, thereby producing the final response.

4 $$\begin{array}{*{20}c} {p_{n} \left( {z{|}x} \right) \propto \exp \left( {d_{\left( z \right)}^{{{\rm T}}} q_{\left( x \right)} } \right)} \\ \end{array}$$

### Applications of RAG and fine-tuning techniques in metaverse systems

RAG and fine-tuning techniques supported by LLMs have been widely adopted in many fields in recent years. RAG provides effective solutions using information obtained from external data sources, particularly for tasks such as open-ended question answering, corporate search engines, and contextually correct answers. On the other hand, the fine-tuning technique allows for the development of task-oriented and personalized AI applications by training LLMs on domain-specific dialogues. In the metaverse, both methods are actively used in voice-based interactions in AI-powered NPCs, guidance services, and real-time virtual assistant scenarios. Table 1 presents a series of studies showing the basic applications of LLMs in virtual environments.

**Table 1:** Use of LLMs in virtual platforms.

Reference	Title	Topic	Technique
^66^	Developing an immersive game-based learning platform with generative AI and VR technologies – “LearningverseVR”	RAG-supported LLM-based learning platform was developed through the LearningverseVR application	LLMs – RAG GPT-3.5 APIs
^50^	AI-enhanced interview simulation in the metaverse: Transforming professional skills training through VR and generative conversational AI	The outputs obtained using the GPT, Flan-T5, and BlenderBot APIs were presented in a job interview scenario	LLMs—RAG GPT, Flan T5, BlenderBot APIs
^54^	LLMs in Eduverse: LLM-Integrated English educational game in metaverse	The use of an English game-based platform developed on the Roblox metaverse was examined	GPT-3.5 Turbo APIs
^26^	DriveRP: RAG and prompt engineering embodied parallel driving in cyber-physical-social spaces	With the DriveRP application, autonomous driving and digital twin data were analyzed using an LLM and RAG-supported model	GPT-RAG
^29^	Fostering design thinking mindset for university students with NPCs in the metaverse	The impact of NPCs in the metaverse environment on students’ design thinking skills was investigated	GPT-3 APIs

The studies presented in Table 1 provide an overview of LLMs-based research in the field of virtual environments. The review examines how LLMs are utilized on virtual platforms. The findings indicate that these models are predominantly accessed through APIs. Instead of fine-tuning or using RAG, LLMs are typically integrated through existing API services. This shows that interactions between users and NPCs are primarily carried out through APIs. The notable point is that none of these applications use fine-tuning techniques but instead rely on the RAG technique. While RAG enriches LLMs with contextual depth by retrieving and integrating information from external sources, fine-tuning may be more effective for improving performance in domain-specific tasks or scenarios. This may explain why RAG is preferred in such cases. In this context, it is valuable to compare RAG and fine-tuning techniques in the use of metaverse-based NPCs to understand the rationale behind their selection. In particular, it is very important to evaluate how NPCs can provide concise, contextually relevant, and semantically consistent responses to users. This study aims to address this need.

## Natural language interaction in metaverse environments

This section begins by providing fundamental information about metaverse technology. Next, it investigates the role of AI-NPCs in virtual environments. Finally, it examines and evaluates the use of LLMs through these NPCs.

### Metaverse technology

Technological developments in the digital age have paved the way for innovations that profoundly affect social structures. One such innovation is the concept of the ‘metaverse,’ which refers to a new digital universe where virtual and physical realities converge. In these environments, where users interact through personalized avatars, the potential for transformation extends beyond social interaction to areas such as education, health, commerce and entertainment. During this transformation process, AI and DL algorithms play a critical role in making metaverse environments smarter, more adaptable, and more interactive^3^.

The term ‘metaverse’ was first used in Neal Stephenson’s science fiction novel Snow Crash and has since transcended the realm of fiction to become a technological reality^52^. Today, this concept refers to persistent digital spaces where users can interact with each other and with computer-generated virtual environments in real time. In the literature, the metaverse is typically discussed in terms of three dimensions: companies’ digital presence, activities conducted in virtual environments, and the convergence of advanced technologies such as VR, AR, AI, and blockchain into a single digital ecosystem. In this sense, the metaverse is not merely a technological trend but also a significant milestone in the digital transformation of social life^15^.

The development of the metaverse is not limited to the experiences provided by VR headsets; rather, it is shaped by the convergence of various technologies such as AR, digital twins, blockchain, AI, and token-based economies. This integrated structure enables users to create virtual identities, produce and trade digital assets, and participate in the construction of a new creative economy^79^. Advances in areas such as speech recognition and natural language generation further enhance the realism and inclusivity of the metaverse experience. In conclusion, the metaverse, an important component of digital transformation, presents new opportunities and challenges at both the individual and organizational levels^78^.

### Metaverse and NPCs

**Fig. 6:**
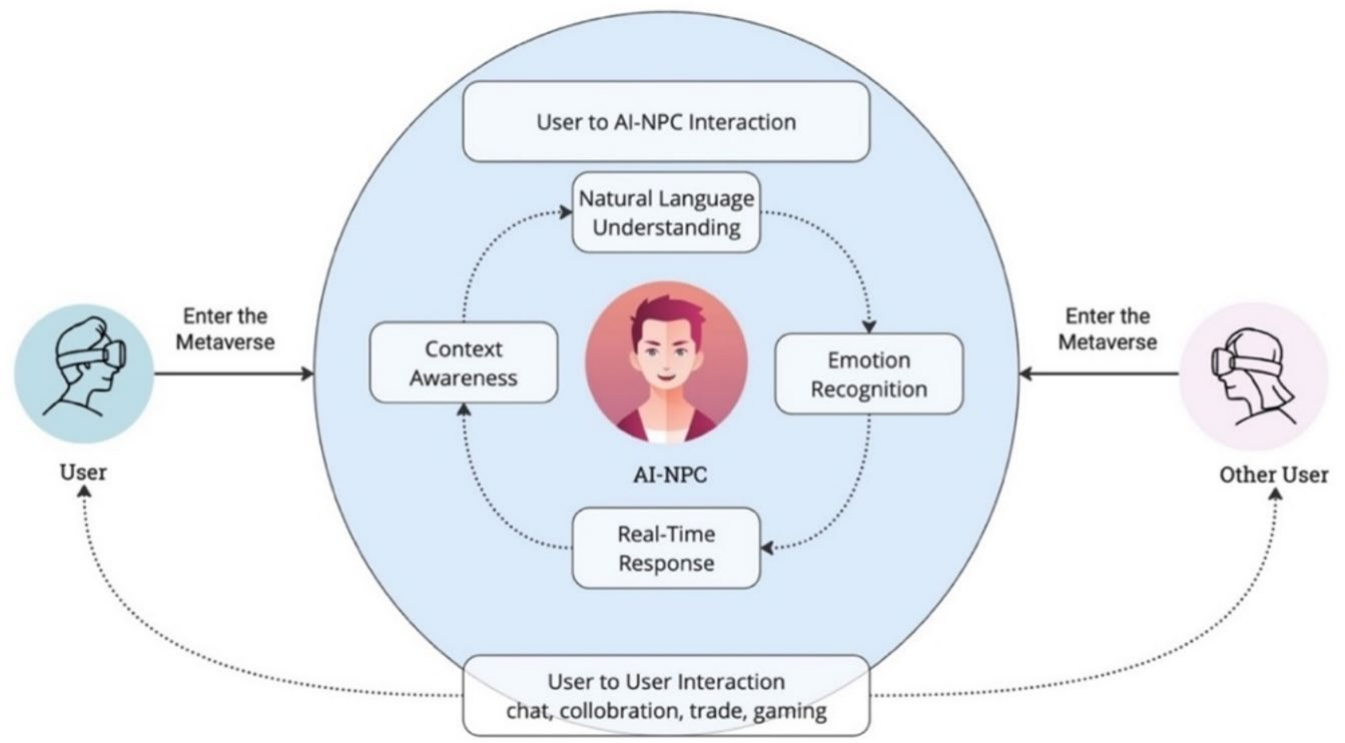
The role of NPCs in interaction within the metaverse.

NPCs are AI-powered virtual characters designed to enhance the user experience in metaverse technologies ^21^.These characters are not just humanoid chatting robots, but also interactive agents equipped with contextual understanding, responsive behaviour, and NLP technologies. As a result, their interactions with users become more natural meaningful, and realistic. Figure 6 illustrates the AI-powered roles of NPCs in metaverse environments and the methods they use to interact with users.

Figure 6 shows the interaction structure between AI-powered NPCs and users in the metaverse environment. This figure presents a two way communication model in which users can interact with both other users and AI-NPCs after entering the metaverse. In this interaction process, AI-NPCs are equipped with three core capabilities: natural language understanding, emotion recognition, and contextual awareness. These capabilities enable NPCs to analyze users’ emotional states, comprehend the meaning of their expressions, and generate real-time responses appropriate to the digital context in which they find themselves.

As a result, interactions are not only scenario-based but also personalized and context-sensitive. In this context, AI-NPCs are not merely information providers in metaverse environments; they also function as agents that enrich the user experience and enhance immersion^1,2^. This interactive structure enables virtual activities such as education, gaming, collaboration, and commerce to be implemented in a more realistic and effective manner. AI-powered characters engage in authentic dialogues that go beyond predefined scenarios. These applications not only increase user engagement but also contribute to the creation of more realistic and participatory metaverse environments^51^. These models enable NPCs to understand context, interpret user intent, and generate responses tailored to individual experiences.

### NLP interaction via LLMs in metaverse environments

NLP is a rapidly developing branch of AI that bridges the gap between human language and machines. This technology is redefining the user experience by enabling more personalized, effective, and human-like interactions in virtual environments such as the metaverse^45^. In particular, the integration of LLMs into NLP significantly improves communication between users and AI-powered characters in the metaverse, both in terms of content depth and interaction quality.

LLMs have the ability to generate meaning and create responses by analyzing the contextual, semantic, and structural features of NLP . This ability enables AI-powered NPCs in the metaverse to engage in real-time, meaningful, and personalized interactions with users^29^. Thanks to LLMs based NLP systems, multilingual communication becomes possible, and language barriers between users are effectively eliminated. Real-time translation, sentiment analysis, and content generation capabilities contribute to making the metaverse a more global and inclusive social space.

The impact of LLMs based NLP technologies on the metaverse is multifaceted. In educational settings, virtual instructors can provide personalized learning experiences tailored to individual students. In the healthcare sector, virtual advisors can analyze medical records to provide early diagnosis and guidance. In the gaming and entertainment industries, LLMs-supported characters can engage users in dynamic, unscripted dialogue that goes beyond predefined scenarios. All of these applications contribute to making metaverse environments more realistic and participatory, in addition to increasing user engagement^51^. Thanks to NLP approaches pioneered by models such as Embeddings from Language Models (ELMo), Bidirectional Encoder Representations from Transformers (BERT), and Guaranteed Pension Trust (GPT), and further advanced by more sophisticated large language models such as Roberta Place Long-Term Care Home Mass Tort (RoBERTa), Text-To-Text Transfer Transformer (T5), Predicting Actions through Language Models (PaLM), and Claude AI (Claude), NPCs in metaverse environments are no longer limited to predefined dialogues. These models enable NPCs to understand context, interpret user intent, and generate responses tailored to individual experiences^19^.

## Method

This section presents the modelling process and methodology for AI-NPC characters in metaverse environments using LLMs. The datasets and data processing steps applied in the fine-tuning and RAG techniques used in the study are explained. The system architecture of the metaverse platform incorporating AI-NPCs is described. Additionally, the evaluation tools used in the study are introduced, and the application steps of the TOPSIS method, a multi-criteria decision-making normalization technique, are detailed.

### Dataset information

**Fig. 7:**
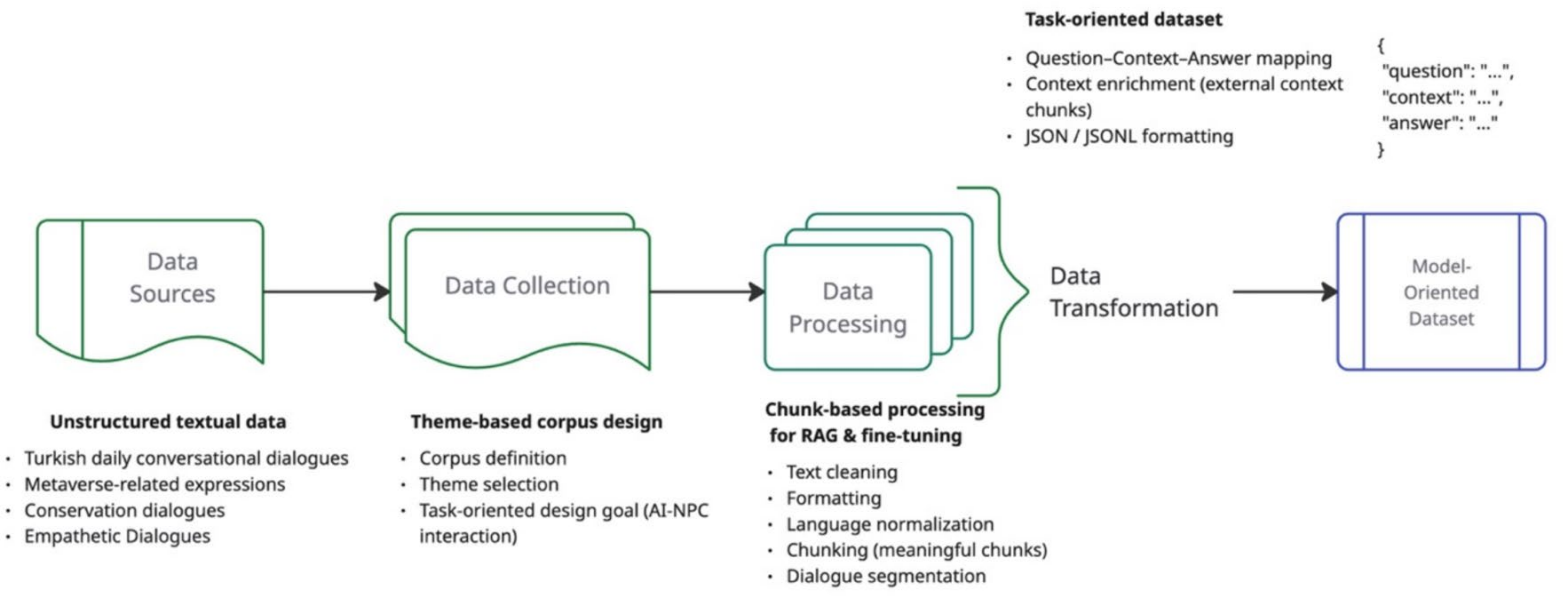
The role of NPCs in interaction within the metaverse.

RAG and fine-tuning processes enable LLMs to generate knowledge-based and task-oriented outputs. Data should be systematically structured to support accurate contextual interpretation. Dataset used in both RAG and fine-tuning should be divided into meaningful segments. Accordingly, the dataset was constructed from daily conversational dialogues related to the metaverse and environmental protection. In this study, the dataset was constructed from daily conversational dialogues and expressions related to the metaverse and environmental protection, as illustrated in Fig. 7.

As shown in Fig. 7, the data preparation process is designed to emphasize short expressions suitable for metaverse environments. The process began with the collection of unstructured Turkish data, including daily conversational dialogues and metaverse-related expressions, supplemented by environmental protection dialogues and empathetic interactions. The dataset supplemented daily with appropriate datasets^32^. These data are then systematically structured to support AI-NPC interactions in metaverse contexts. Following data collection, chunk-based processing is applied for RAG and fine-tuning. Text cleaning, formatting, and language normalization are conducted to ensure consistency. Contextual representation is further enhanced through meaningful segmentation and dialogue structuring. The processed data are organized in a question–context–answer format, and contextual enrichment is achieved using external context chunks. Finally, the dataset is converted into a standardized json/jsonl format to ensure model readiness, and its scope is expanded by incorporating additional question and answer content. Summary information on the final dataset is presented in the Table 2.

**Table 2:** Characteristics of the dataset in this study.

Dataset Component	Corpus Category	Data Quality Note	Frequency (*f*)	Percentage (%)
External Context Chunks (RAG, Fine-tuning)	Emotional Experiences	Low proportion of short answers; high contextual coherence	47,184	84.29
	Social and Daily Dialogues	Limited number of short responses in daily conversations	7,368	13.17
	Daily Life Contexts	Well-balanced questions and answers	866	1.55
	Educational Dialogues	Conceptually consistent questions and answers	223	0.40
	Public Service Dialogues	Moderate risk of short responses	68	0.12
	Work and Economy Dialogues	Structurally clean; sufficient contextual information	150	0.27
	Digital / Metaverse Dialogues	Limited volume; contextually rich	66	0.12
	Daily Informations	Responses are generally short and direct	50	0.09
	Records with Missing Fields	Question, answer, or context field missing	28	0.05
	Records with Short Answers (< 120 characters)	Enriched before RAG and fine-tuning	5,282	9.43

As shown in Table 2, the dataset developed in this study is designed to support task-oriented communication between users and AI-NPCs in metaverse environments. The dataset focuses on multiple domains to enhance linguistic coherence and contextual relevance. Daily dialogue scenarios include common real-life conversational expressions and emphasize language related to navigation, interaction, and guidance within virtual environments.

This dataset structure is specifically designed to improve the model’s ability to generate contextually consistent responses in interactive scenarios. The dataset particularly emphasizes short yet meaningful expressions that are well suited for real-time AI-NPCs interactions in immersive environments. To support both RAG and fine-tuning processes, the dataset is divided into meaningful chunks and structured using a standardized json format. Each data instance consists of three core components: question, context, and answer (5).

5 $$\begin{gathered} {\mathrm{json}} \hfill \\ \{"{\mathrm{question"}}:\;{\mathrm{''What}}\;{\mathrm{is}}\;{\mathrm{metaverse}}?", \hfill \\ {\mathrm{"context}}:\;{\mathrm{"The}}\;{\mathrm{metaverse}}\;{\mathrm{is}}\;{\mathrm{like}}\;{\mathrm{a}}\;{\mathrm{virtual}}\;{\mathrm{world}} \hfill \\ {\mathrm{within}}\;{\mathrm{the}}\;{\mathrm{internet}}.", \hfill \\ {\mathrm{"answer}}":{\mathrm{"It}}\;{\mathrm{is}}\;{\mathrm{a}}\;{\mathrm{type}}\;{\mathrm{of}}\;{\mathrm{online}}\;{\mathrm{universe}}." \hfill \\ \end{gathered}$$

### Data processing

All model training processes in this study were conducted using the Google Colab platform. Python 3 was used as the primary development environment, and an NVIDIA Tesla T4 GPU served as the hardware accelerator. To accommodate the increased memory demands of large-scale model training, Colab’s high-RAM configuration was enabled. During the training processes, approximately 54.8 GB of system memory, 15 GB of GPU memory, and 235 GB of disk space were utilized. These computational resources provided sufficient capacity for the efficient fine-tuning and RAG training of LLMs , while also supporting context-aware and task-oriented learning. In cases where GPU resources were unavailable, the training processes were executed using CPU-based computation.

To increase computational efficiency during training, the batchsize parameter was typically set between 2 and 4. In some cases, the gradientsteps parameter was used to effectively increase the group size in order to provide stable training under limited GPU memory through larger virtual groups. Encoder decoder models such as mBART and mT5 were trained for 5 epochs using the RAG technique, while other models were typically trained for 3 epochs. To reduce memory consumption and accelerate training, mixed-precision (FP16) computation was used on compatible hardware. This feature was disabled in cases of hardware incompatibility. To ensure the traceability and recoverability of the training process, model weights and checkpoints were saved at the end of each epoch. In some large-parameter models, 8-bit quantization and LoRA techniques were used to optimize memory usage and training speed. The libraries used in the RAG and fine-tuning training processes included modern DL tools such as Transformer, Accelerate, FAISS, Sentence-Transformer, PEFT, and BitsAndBytes, which provide flexible and resource-efficient model training. The fine-tuning procedure is summarized in Algorithm 1.

**Algorithm 1:**
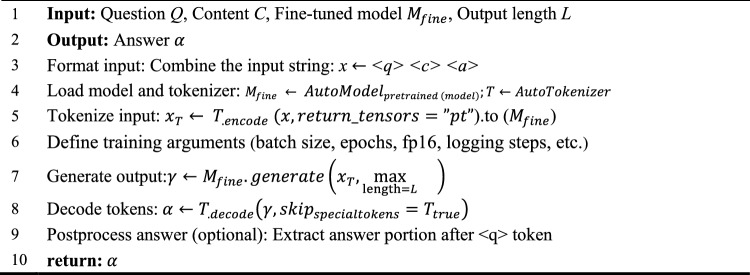
Fine-tuning process for the study.

Algorithm 1 describes the fine-tuning process applied in this study. The process was specifically designed for a question-answering task in accordance with the structure of the customized dataset. It begins with a user-provided question (*Q*) and its associated context (*C*), which are merged into a single text sequence using special tags *(*< *q* > *,* < *c* > *,* < *a* >). This combined sequence is then tokenized and tensorized using a tokenizer to prepare it for processing by the fine-tuned model ${(M}_{fine}$), which is loaded via the hugging face library^28^. Training parameters such as batch size, number of epochs, precision mode (FP_16_), and logging steps are defined. The model then generates a response based on the provided input, constrained by a specified maximum sequence length (*L*). The output is subsequently detokenized back into natural language format, and optionally, only the answer portion may be extracted and returned.

Algorithm 2 summarizes the steps involved in applying the RAG technique. The process begins with a query (*Q*), its corresponding context (*C*), a pre-trained language model ${(M}_{pre}$), a tokenizer (*T*), and a maximum output length (*L*). In the first step, these components are concatenated into a single input string *x* using special tags *(*< *q* > *Q* < *c* > *C* < *a* >*)*. Then, the model and tokenizer are loaded using the *AutoModelForSeq2SeqLM* and *AutoTokenizer* classes. To ensure correct token alignment and sequence formatting, the tokenizer’s pad token is explicitly set to match the eos token. The formatted input is then tokenized and converted into a tensor ${x}_{T}$ suitable for model input. This tensor is passed to the model’s *generate()* function, which produces an output sequence within the specified maximum length (*L*). The resulting token sequence is decoded into natural language using the tokenizer’s *decode()* method. If necessary, post-processing is applied to refine the output. Finally, the generated response α is returned.

**Algorithm 2:**
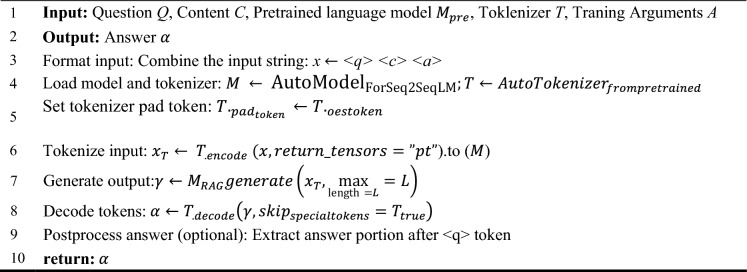
RAG process for the study.

**Fig. 8:**
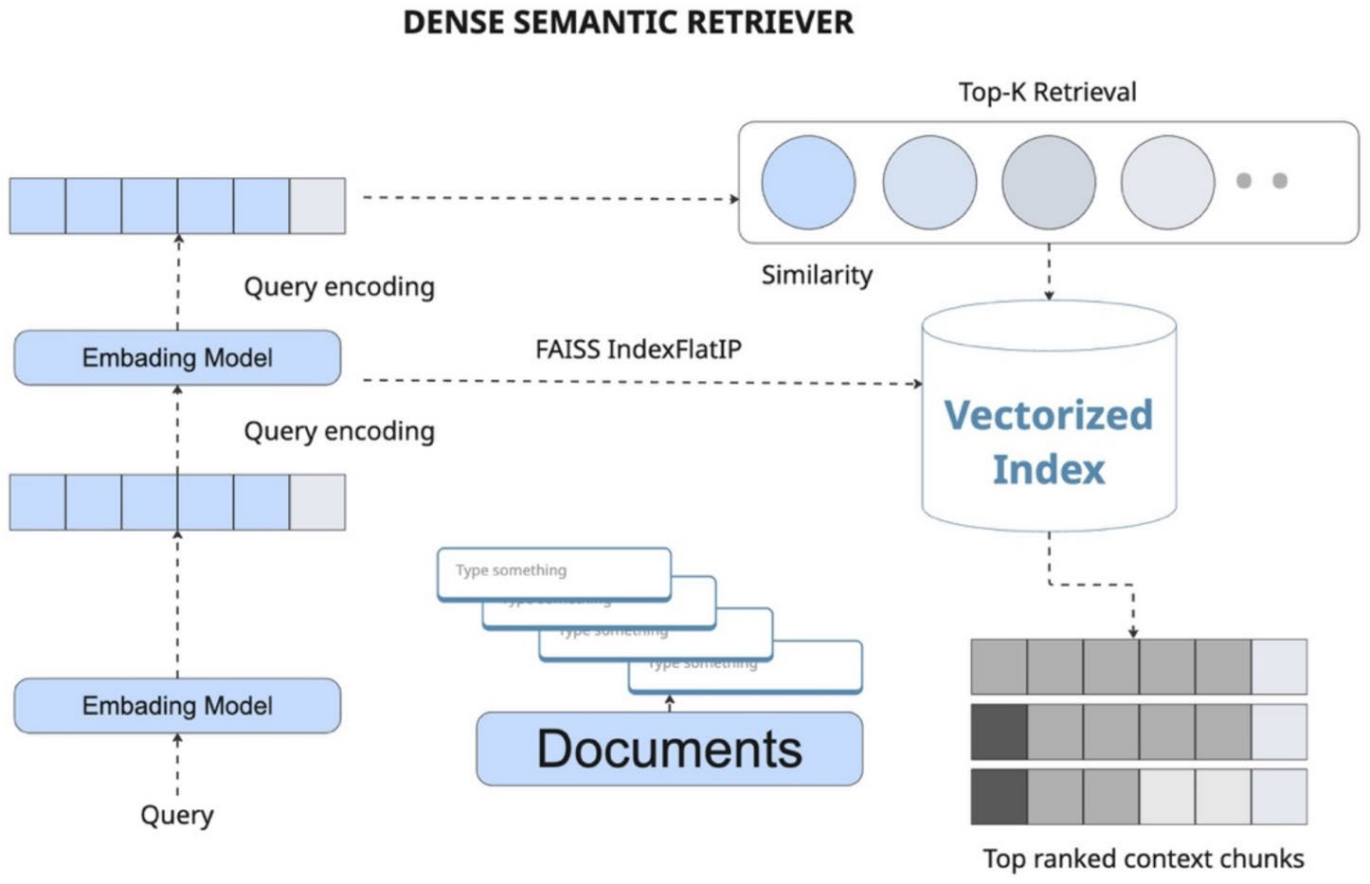
Dense retriever and FAISS Index.

In this study, dense retrieval is used in the RAG training process. Dense retrieval represents queries and documents as vector embeddings and matches them based on keywords similarity. This approach allows the model to retrieve more contextually relevant information. The dense retrieval structure used in the study is shown in the Fig. 8.

As shown in Fig. 8, the RAG process starts by converting the user query into a dense vector using an embedding model. Depending on the model, documents are either split into smaller parts using a character-based sliding window or treated as a single context unit. These document parts are then transformed into dense document vectors and indexed using FAISS IndexFlatIP. By normalizing the embeddings, the inner product calculation corresponds to cosine similarity. The query vector is compared with the indexed document vectors, and similarity scores are used to rank the results. A cosine similarity–based Top-K retrieval strategy (K = 3 or 4) is applied to return the most relevant context segments. In addition, Algorithm 2 presents an RAG-based answer generation process that produces answers based on the given question and related context.

Algorithms 1 and 2 are structured to serve the main purpose of this work. They are designed to facilitate the execution of multi-step tasks that support contextual text generation in metaverse-based interactions. In this context, the algorithms in this work are geared towards effectively exploiting the context-driven generative capabilities of LLMs. AI-NPC characters answer questions posed at the relevant context point within this framework.

### Metaverse system architecture

In metaverse environments, the integration of NLP and LLMs approaches is essential for enabling effective and natural interactions between users and AI-NPC characters^50^. Within these virtual settings, voice emerges as the primary medium of communication between humans and AI-NPCs. These voice-based interactions, which facilitate users’ active participation in the metaverse, generally occur in two stages: The first is Speech-to-Text (STT), which converts spoken signals into written text; the second is Text-to-Speech (TTS), which transforms text into speech signals.

**Fig. 9:**
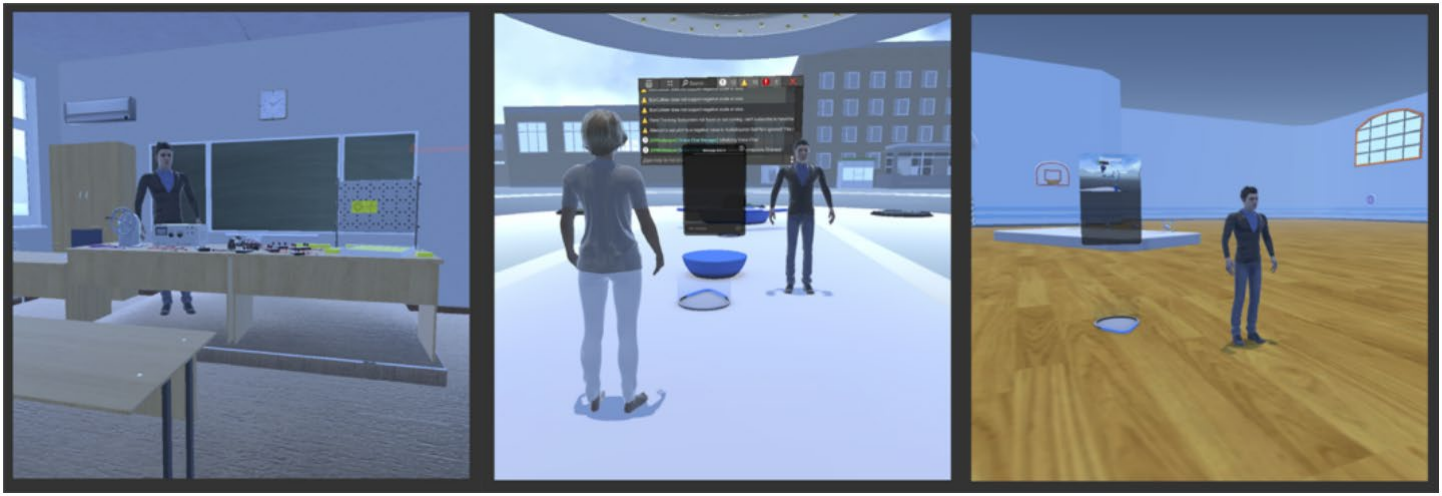
Visualization of the metaverse environment developed within the scope of the study.

The system architecture supporting this structure is structured to enable communication between both human users and AI-NPCs . It is designed to allow users to interact with AI-NPCs in the metaverse environment and acquire relevant information through these interactions. Visual representations of the developed environment are presented in Fig. 9.

**Fig. 10:**
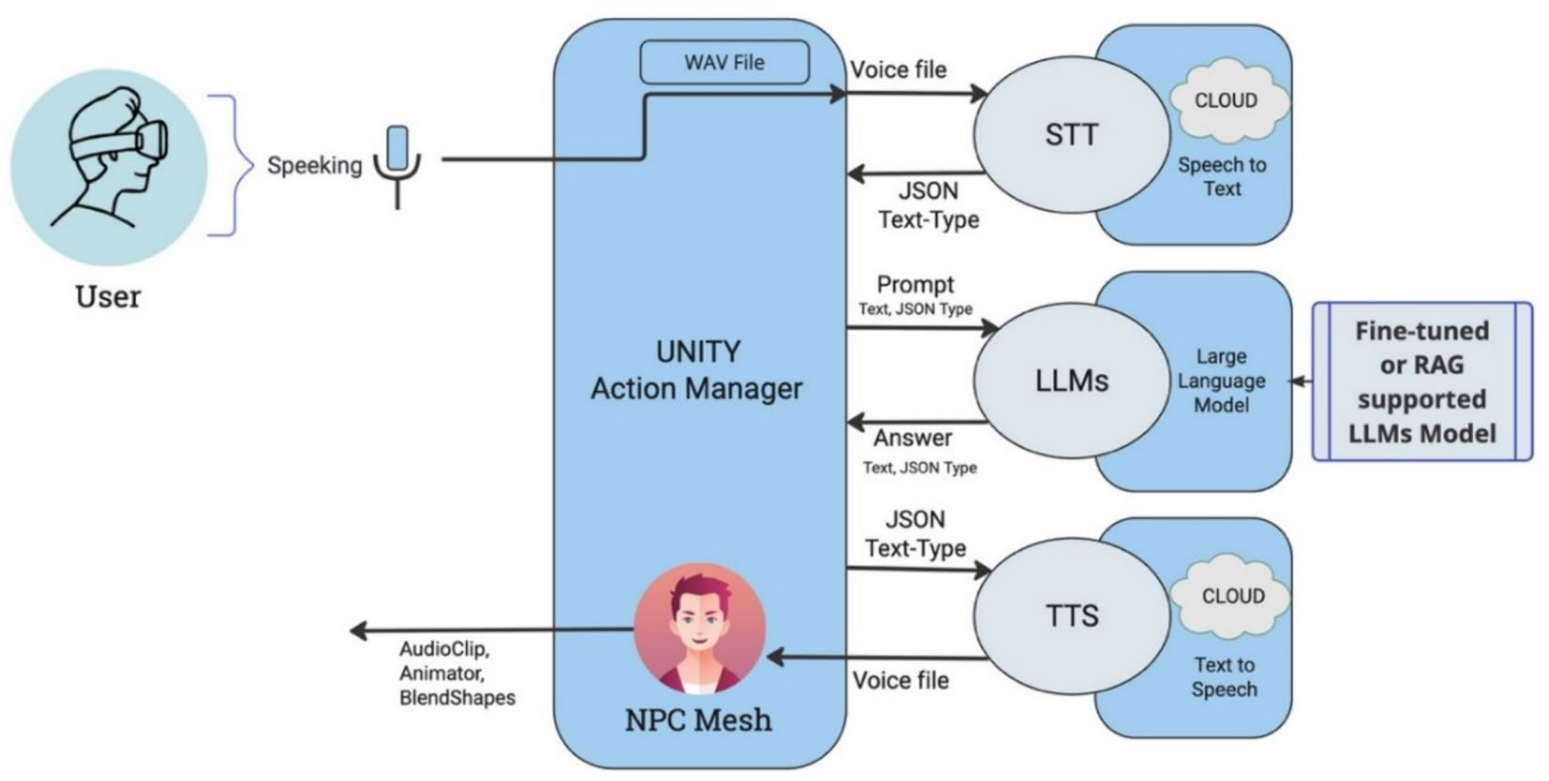
Designing speech-enabled AI-NPCs environments with LLMs.

As seen in Fig. 9, the platform developed in this study allows up to participants to access the system simultaneously through VR headsets. In addition, users experience natural interaction through voice-based communication with AI-NPCs . As technical perspective, the movements and animations of these AI-NPCs are typically managed using advanced game engines such as Unity^71^ or Unreal Engine^72^. The system architecture implemented in this study integrates STT, LLMs and TTS components and is shown schematically in Fig. 10.

Figure 10 illustrates the real-time processing of users’ voice-based interactions in the metaverse environment and the transformation of these interactions into both audio and visual outputs through cloud-based AI systems. With the help of interaction tools such as virtual reality headsets, users can navigate the 3D environment and engage in natural verbal communication with AI-NPCs.

Communication with AI-NPCs starts with the STT component converting audio signals into structured text. The resulting text is then processed by LLMs trained using RAG and fine-tuning techniques to generate a context-appropriate response. This response is converted back into audio output through the TTS system. If relevant content is not found in the system’s knowledge base, the response is generated based on the LLM’s pre-trained grammar and general world knowledge, or through fallback responses such as “I don’t know”. As a result, the dialog between the user and the AI-NPCs is maintained in a manner consistent with NLP .

The generated response is synchronized with both auditory and visual expressions through the integration of audioClip, animator, and blendShapes components embedded in the mesh structure of the AI-NPCs. This setup enables the delivery of realistic responses enriched with facial animations and expressions. As a result, users receive not only content-driven but also visually and emotionally engaging feedback, enhancing the naturalness of the interaction. For this communication, fine-tuned and RAG techniques adapted by the researcher were used to support question answering tasks consistent with the scope of the study. As a result, the AI-NPCs in the system are able to produce context-appropriate, meaningful and consistent responses in Turkish natural language processing tasks.

### Large language models used in this study

LLMs are probabilistic structures that model the joint probability distribution of sequences of text units^59^. These models predict the probability of words occurring together in a given order by predicting the next token relative to its predecessors, thus enabling the generation of new and meaningful word sequences. In addition to text generation, LLMs have demonstrated high performance in various tasks such as sentiment analysis, auto-completion in email or messaging services, and dialog-based AI applications^67^. In this work, the evaluation is focused on the question answering capabilities of AI-NPC characters in the metaverse environment.

LLMS are generally categorized into three main groups based on their architectural design: encoder-only, decoder-only, and encoder-decoder models^83^. Models with an encoder-decoder architecture utilize bidirectional encoder layers to deeply analyze the input, while employing unidirectional decoder layers to generate output sequentially. This structure provides effective results in both language understanding and generation tasks^35^. Table 3 provides detailed information on the three architectures and examples of representative models.

**Table 3:** Comparison of LLMs architectures.

Features	Encoder-only LLMs	Decoder-only LLMs	Encoder-Decoder LLMs
Architecture	Encoder layers only	Decoder layers only	Encoder + Decoder layers
Purpose	Understanding tasks (e.g., classification, analysis)	Text generation and completion tasks	Input understanding and output generation
Text generation	–	Autoregressive generation	Sequence-to-sequence (seq2seq) generation
Training objective	Masked language modeling (MLM)	Next token prediction (NTP)	Sequence-to-sequence (seq2seq)
Strengths	Deep contextual understanding	Strong for fluent text generation	Effective for translation, summarization
Weaknesses	Ineffective for generation tasks	Limited contextual comprehension	Higher computational cost
Example	BERT, DeBERTa	GPT, Llama, Bard, Alpaca, Qwen, Mistral, Palm, Gemini, Claude	MT5, Flan-T5

Table 3 presents a comparative overview of LLMs architectures. Encoder-only models are primarily effective in comprehension-based tasks such as classification and analysis, and are typically trained using masked language modeling. Main strength lies in their capacity to deliver deep contextual understanding; however, they are limited in their ability to generate text. In contrast, decoder-only architectures are used for tasks involving text generation and completion. These models work through autoregressive generation, allowing them to produce coherent text sequences, but their contextual understanding is relatively narrow. Encoder-decoder models are designed to perform both input comprehension and output generation tasks. Trained through input–output mapping, these models are particularly effective in seq2seq applications such as translation and summarization. However, these architectures tend to incur higher computational costs.

In this study, decoder-only and encoder-decoder architectures capable of both text generation and completion and input understanding and output generation are used. These architectures are compatible with fine-tuning and RAG based training processes. Accordingly, the objective was to enhance the models’ question-answering capabilities. Table 4 presents the characteristics of the models employed in this study along with the training approaches utilized.

**Table 4:** Decoder-only and encoder-decoder LLMs models used in the study.

Model Structure	Models	Number of parameters	Fine-tuning Method	RAG Retriever Method
Decoder-only	GPT-2	125M*	Fully fine-tuning	FAISS L2
	Qwen	1.5B*	LoRA + FAISS	FAISS L2
	Mistral	7B*	8-bit quantization + LoRA	FAISS L2
Encoder-Decoder	MBART	610M*	Fully fine-tuning (seq2seq)	FAISS L2
	MT5	300M*	Fully fine-tuning (seq2seq)	FAISS L2

* M: million, B: billion.

Table 4 summarizes the architectures, parameter sizes and implementation strategies of the LLMs used in this study. This study incorporates not only decoder-only architectures such as GPT-2, Qwen, and LLaMA, but also encoder-decoder architectures, including mBART and mT5. Models such as GPT-2 and mBART were adapted through full fine-tuning. In contrast, more lightweight and computationally efficient methods—such as LoRA and 8-bit quantization—were employed for models like Qwen and LLaMA. Additionally, during the retriever phase of the RAG training process, all models utilized a FAISS L2-based retrieval technique to support information retrieval.

### Evaluation metrics of RAG and fine-tuning

This study explores the capacity of AI-powered NPCs deployed in metaverse environments to generate concise, meaningful, and task-oriented responses to user queries in natural language. To this end, the evaluation metrics used to assess the developed language models are summarized in Table 5.

**Table 5:** Evaluation metrics used in the study.

Metric Name	Category	Primary Purpose	Description
Perplexity Score	Content Quality	Measures how well a language model predicts the next word	Lower perplexity indicates more fluent and predictable text
Layer-Freezing Perf	Computational Efficiency	Evaluates which layers to train during transfer learning	Aims to reduce computational cost while maintaining similar performance
BLEU	Content Quality	Measures n-gram overlap between generated and reference text	Common in machine translation and summarization tasks
METEOR	Content Quality	Improves upon BLEU by accounting for semantics and word order	Considers synonyms, stemming, and word alignment
ROUGE-L	Content Quality	Measures overlap between summaries and references	Uses longest common subsequence for comparison
Inference Time	Computational Efficiency	Measures how fast a model generates a response	Crucial for real-time applications
BERTScore	Semantic Quality	Measures semantic similarity between generated and reference text	Computes cosine similarity using BERT-based embeddings
BLEURT	Semantic Quality (Human-like)	Produces scores aligned with human judgments	Effective for evaluating natural language generation
DialogRPT	Interactional Relevance	Evaluates how engaging or relevant a dialogue response is	Predicts user engagement using a Reddit-trained model

Table 5 summarizes the evaluation metrics utilized for various analytical purposes within this study. The selected metrics jointly evaluate dialogue generation performance from complementary perspectives, including fluency and linguistic coherence (Perplexity), lexical alignment with reference responses (BLEU, ROUGE-L), semantic adequacy beyond surface overlap (METEOR, BERTScore, BLEURT), interactional relevance in conversational settings (DialogRPT), and practical deployment constraints such as computational efficiency and real-time responsiveness (layer-freezing performance and inference time). This multi-dimensional framework ensures a balanced and dialogue-oriented evaluation of both model quality and system usability. A detailed description and justification of the selected evaluation metrics are provided in Appendix C (Evaluation metrics used in the study)**.**

Generally, perplexity assesses the predictive accuracy and naturalness of language models, with lower scores indicating superior performance^33,34^. Layer freezing involves fixing the weights of certain layers during fine-tuning to optimize training efficiency (about 50% in this research), especially useful for limited computational resources or small datasets^16^. The BLEU score measures output quality by evaluating n-gram precision between generated texts and reference materials, particularly in translation and text-generation tasks. METEOR offers semantic depth by evaluating exact matches, synonymy, stemming, and semantic interpretation. ROUGE-L assesses summarization effectiveness based on the longest common subsequence overlap between generated and reference summaries, primarily emphasizing lexical matches^8^. Inference time denotes the duration required for a model to produce outputs from given inputs. BERTScore evaluates semantic similarity using contextual embeddings from the BERT model, focusing on deeper meaning rather than mere lexical overlap. BLEURT, trained as a regression model, quantitatively assesses both fluency and semantic accuracy closely aligned with human judgment^50^. Finally, Dialogue RPT ranks dialogue response quality by leveraging models fine-tuned on preference-based datasets^17^.

### The normalization process within the TOPSIS approach

The TOPSIS method, originally introduced by Hwang et al.^30^, is a widely used approach for solving multi-criteria decision-making problems. The basic principle of TOPSIS is to evaluate alternatives based on their relative proximity to a positive ideal solution (representing the best-case scenario) and distance from a negative ideal solution (representing the worst-case scenario). By measuring these distances, the method allows alternatives to be ranked to reflect their proximity to the optimal decision^39^.

In this study, the TOPSIS method was applied to integrate results derived from multiple evaluation metrics into a unified, comparable structure. The process began with the construction of a decision matrix (6), followed by the normalization of each criterion column based on its Euclidean norm (7). In the third step, a weighted decision matrix was formed using the normalized values, and both the ideal (maximum) and anti-ideal (minimum) values for each criterion were determined (8). Finally, the distances of each alternative to the ideal and anti-ideal solutions were calculated, and a relative closeness score was derived accordingly. The mathematical expressions corresponding to these computations are presented below.

6 $$\begin{array}{*{20}c} {X = \left[ {x_{ij} } \right]} \\ \end{array}$$

7 $$\begin{array}{*{20}c} {r_{ij} = \frac{{x_{ij} }}{{\sqrt {\mathop \sum \nolimits_{i = 1}^{m} x_{ij}^{2} } }}} \\ \end{array}$$

8 $$\begin{array}{*{20}c} {v_{ij} = w_{j} . r_{ij} } \\ \end{array}$$

In the equations above, the term ($${x}_{ij})$$ denotes the value of the $$\left(j\right)$$ criterion for the $$(i$$) alternative. Here, $$m$$ represents the total number of alternatives, $$n$$ while indicates the total number of criteria. The normalized value $${r}_{ij}$$, derived during the normalization phase, constitutes the element of the normalized decision matrix and is scaled within the range of 0 to 1. In the weighted normalization step, $${w}_{i}$$ refers to the weight assigned to the $$\left(j\right)$$ criterion, and $${v}_{ij}$$ represents the corresponding weighted normalized value. This weighting process ensures a fairer evaluation by incorporating the relative importance of each criterion into the decision-making model. The TOPSIS scores were obtained by applying the decision matrix presented in Table 6, and the resulting TOPSIS decision matrix is provided below.

**Table 6:** TOPSIS decision matrix.

Model	Perplexity Score	Layer-Freezing Perform	Evaluation Metric Scores			Inference Time (s)	BERT Score	BLEURT	DialogRPT
			BLEU	METEOR	ROUGE-L				
mBART	2.48	+ 0.027	0.352	0.749	0.110	1.31	0.582	0.290	0.648
LLaMA	4.80	−0.002	0.114	0.395	0.226	0.51	0.515	0.269	0.452
GPT-2	6.26	−0.014	0.085	0.518	0.184	0.59	0.594	0.592	0.476
mT5	2.43	−0.002	0.004	0.004	0.184	0.39	0.367	0.118	0.619
Qwen	9.25	+ 0.004	0.001	0.036	0.014	0.39	0.338	0.293	0.394

Table 6 presents the decision matrix used as input for the TOPSIS analysis, encompassing all evaluation criteria along with their corresponding optimization directions. This explicit formulation enables the normalization, weighting, and ranking procedures to be systematically and reproducibly applied within the multi-criteria evaluation framework.

## Results

This section presents AI-NPCs response mechanisms developed using decoder-based models (GPT-2, Qwen, and LLaMA) and encoder–decoder models (such as mBART and mT5) that integrate RAG and fine-tuning techniques. Detailed training results are provided in Appendix A (RAG training loss curves), Appendix B (fine-tuning training loss curves), Appendix D (parameter updates), and Appendix E (model-generated responses). This section summarizes the key experimental findings and provides a comparative performance analysis of the models developed using fine-tuning and RAG strategies.

### RAG-based generation performance results of LLMs

Information on the RAG training results is reported in Appendix A. Training and loss analyses reveal taht a common pattern among decoder-only models. They are characterized by a rapid initial loss decrease followed by slow and prolonged convergence, with micro-fluctuations and pronounced plateau tendencies in the loss curves. GPT-2 struggles to effectively distinguish retrieved documents in the RAG setting, resulting in stable but limited loss reduction, while Qwen reaches early saturation around a loss value of approximately 1.0, indicating shallow early learning with limited semantic depth. Among the decoder-only models, LLaMA exhibits the most favorable loss curve, reflecting strong pre-training representations; however, its learning trajectory suggests limited architectural alignment with RAG-based structural knowledge integration.

In contrast, encoder–decoder models demonstrate qualitatively different learning behaviors. mBART shows a very high initial loss followed by a sharp early decrease and subsequent stabilization along a nearly flat trajectory, closely aligning with the core principles of the RAG . The mT5-Small model displays slower yet highly regular and monotonic loss reduction, indicating architectural suitability for RAG despite limited capacity for representing complex contexts.

RAG-based, knowledge-intensive, and task-oriented generation scenarios, encoder–decoder architectures—particularly mBART and mT5—exhibit more stable learning dynamics, lower uncertainty (perplexity), and higher semantic accuracy than decoder-only models. The results obtained from conventional evaluation metrics applied to the models are presented in Table 7.

**Table 7:** Conventional RAG-based generation performance results.

Model Structure	Model	Perplexity Score	Layer-Freezing Perform	Evaluation Metric Scores			Inference Time (s)
				BLEU	METEOR	ROUGE-L	
Decoder-only	GPT-2	7.26	0.144	0.185	0.418	0.184	1.109
	LLaMA	4.80	0.220	0.114	0.395	0.226	2.269
	Qwen	9.25	0.147	0.124	0.265	0.145	0.599
Encoder-Decoder	mBART	2.48	0.270	0.352	0.549	0.210	1.310
	mT5	2.43	0.267	0.418	0.420	0.184	0.899

The results presented in Table 7 demonstrate the evaluation outcomes of model training conducted using the RAG technique. These outcomes were assessed through both conventional metrics such as Perplexity, Layer-Freezing Performance, BLEU, METEOR, ROUGE-L, and inference time. According to the findings, models based on encoder-decoder models outperformed decoder-only models. Among these, mBART and mT5 achieved notably low perplexity scores $$\left({mBART}_{Perp.}=2.48,{mT5}_{Perp.}=2.43\right)$$, indicating a stronger predictive capacity and a higher degree of fluency in natural language generation. These results suggest that both models are capable of producing more coherent and human-like text. In addition, both models demonstrated superior layer freezing performance $$\left({mBART}_{L-F.Perf.}=0.27,{mT5}_{L-F.Perf. }=0.267\right)$$, implying high potential for transfer learning. Transfer learning refers to the reuse of knowledge gained from a large dataset to improve performance on a more specific task using a smaller dataset. The mT5 model achieved a BLEU score of 0.418, indicating a strong alignment between the generated outputs and the reference texts. On the other hand, mBART achieved the highest METEOR score ($${mBART}_{METEOR}=0.549)$$, demonstrating superior performance in semantic and root-based similarity and flexible word order consistency.

In contrast, the performance of models containing only decoders appears to be relatively limited. For example, Qwen exhibited a high complexity score $\left(Qwe{n}_{Perp.}=9.25\right)$ and low layer freezing performance $\left(Qwe{n}_{L-F.Perf. }=0.147\right)$, indicating that this architecture is insufficient in terms of overall model effectiveness. Although the LLaMA model achieved a relatively better ROUGE-L score $\left({LLaMA}_{Rouge-L }=0.226\right)$, indicating structural overlap in word sequences, it is less suitable for practical application scenarios requiring fast responses due to its high inference time $\left({LLaMA}_{I-time }=2.27\right)$. Similarly, GPT-2 performed poorly in both BLEU and METEOR evaluations $\left({GPT-2}_{BLEU}=0.185,{GPT-2}_{METEOR.}=0.418\right)$ and fell short in terms of reference similarity, root-based semantic alignment, and flexible word order generation.

The encoder-decoder models mBART and mT5 outperform other evaluated models in generating concise, task-oriented responses, demonstrating superior accuracy, semantic consistency, natural language generation capabilities, and strong transfer learning potential. These models particularly excel in short question–answer scenarios and maintain high adaptability across diverse linguistic contexts. While Qwen may be preferred for applications requiring quicker inference at the expense of output quality, LLaMA’s advantages in summarization and contextual accuracy are counterbalanced by its slower response time, necessitating careful consideration. Traditional evaluation metrics such as BLEU, ROUGE-L, and Layer-Freezing Performance, based solely on average scores, inadequately measure the accuracy and contextual consistency of brief, task-oriented responses^31,48,56^. Consequently, traditional text similarity-based evaluation methods prove insufficient for assessing concise and semantically coherent outputs, highlighting the need for modern evaluation tools incorporating contextual and semantic dimensions. In this context, the study employs advanced evaluation metrics (BERTScore, BLEURT, and DialogRPT) to comprehensively assess response quality, and the results of these metrics applied to RAG are presented in Table 8.

**Table 8:** Contextual RAG-based Generation Performance Results.

Model Structure	Model	BERTScore	BLEURT	DialogRPT
Decoder-only	GPT-2	0.595	0.403	0.416
	LLaMA	0.516	0.293	0.352
	Qwen	0.338	0.269	0.394
Encoder-Decoder	mBART	0.583	0.590	0.648
	mT5	0.467	0.319	0.419

Table 8 shows the evaluation results of RAG-based performance using semantic-based metrics, including BERTScore, BLEURT, and DialogRPT. In this context, the mBART model demonstrated balanced and effective performance, achieving high scores in all three metrics. It demonstrated strong semantic alignment between words $\left(mBAR{T}_{BERTscore }=0.583\right)$, showed greater consistency with human judgement in response generation $\left(mBAR{T}_{BLEURT }=0.590\right)$, and provided high-quality dialogue output as rated by dialogue modelling systems $\left(mBAR{T}_{DialogRPT }=0.648\right)$. Interestingly, despite GPT-2's low performance on traditional evaluation metrics, it unexpectedly achieved higher scores when evaluated with modern contextual evaluation tools $\left(GPT-{2}_{BERTscore }=0.595\right), \left(GPT-{2}_{BLEURT }=0.403\right),\left(GPT-{2}_{DialogRPT }=0.416\right)$. These findings suggest that GPT-2 can still serve as a functional alternative in scenarios that require short but contextually consistent responses.

In contrast, the mT5 model scored above average on traditional measures, but relatively lower on contextual assessments $$\left(mT{5}_{BERTscore }=0.467\right), \left(mT{5}_{BLEURT }=0.319\right),\left(mT{5}_{DialogRPT }=0.419\right)$$. Similarly, although the LLaMA model performed well on traditional measures such as ROUGE-L, it showed limited success on context-sensitive evaluations $$\left(LLaM{A}_{BLEURT }=0.293\right),\left(LLaM{A}_{DialogRPT }=0.352\right)$$. These findings suggest that both models may be more advantageous for processing longer, structurally rich and formally complex texts; however, they seem to fall short in producing short and direct responses with sufficient contextual depth. The Qwen model performed poorly on both traditional and contextual measures $$\left( {Qwen_{{BERTscore}} = 0.338} \right),$$$$\left( {Qwen_{{BLEURT}} = 0.269} \right),$$$$\left( {Qwen_{{DialogRPT}} = 0.394} \right)$$ , indicating limited suitability for short-form and semantically grounded production tasks.

**Fig. 11:**
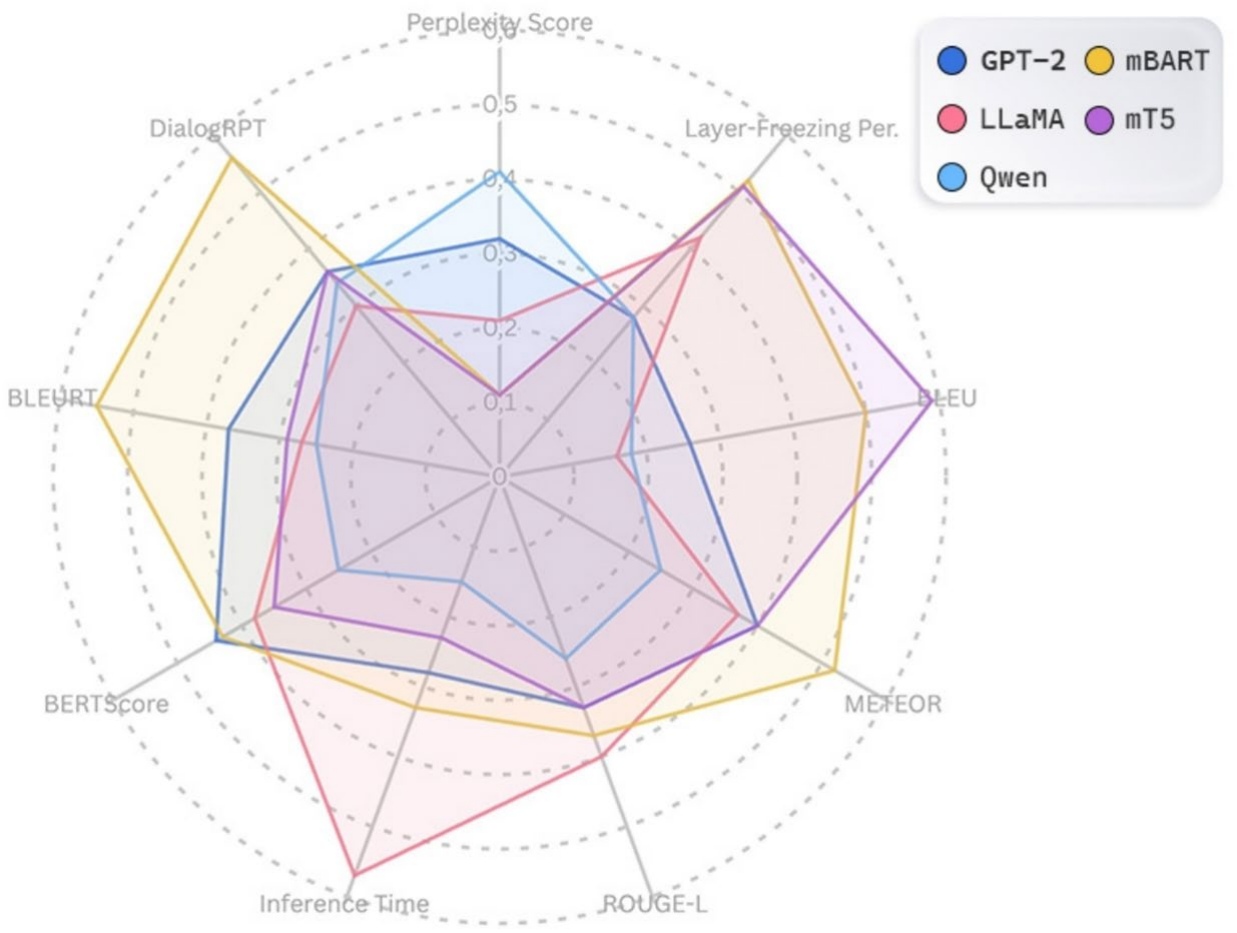
Comparative metrics analysis of LLMs—RAG techniques.

Comparative analysis indicates that the mBART model demonstrates superior performance across both traditional and contemporary evaluation metrics for generating short responses. The model achieves high scores on multiple contextual metrics, reflecting its capability in producing balanced and contextually effective outputs. Additionally, mBART aligns closely with human judgments concerning semantic relevance. Conversely, GPT-2 shows lower scores with classical metrics but surpasses expectations in context-based evaluations, highlighting its utility in brief, context-rich scenarios. Although mT5 and LLaMA models excel according to traditional metrics, their performance declines in contextual assessments, suggesting they are better suited for longer, structured texts rather than brief, direct responses. In contrast, Qwen’s consistently weak results across all metrics indicate its inadequacy for short-response tasks. Figure 11 visually summarizes these performance differences among the five LLM models (GPT-2, LLaMA, Qwen, mBART, and mT5) via a radar chart, standardized by the TOPSIS mean method. It is important to note metric-specific interpretations: lower scores in Perplexity and Inference Time indicate better performance, whereas higher scores are preferred for BLEU, ROUGE-L, and BERTScore metrics. Therefore, directional differences in these metrics must be appropriately considered during analysis.

Figure 11 visually summarizes standardized evaluation results of models employing the RAG technique, highlighting that encoder-decoder architectures generally exhibit superior performance. Specifically, the mBART model, depicted prominently in the radar chart, achieves high scores across both traditional metrics (BLEU, METEOR, ROUGE-L) and contextual evaluations (DialogRPT, BLEURT), alongside a strong BERTScore. Despite these strengths, mBART demonstrates lower performance in Perplexity and Inference Time metrics, indicating reduced linguistic ambiguity and faster response generation. Conversely, the Qwen model, occupying the smallest area on the chart, consistently underperforms across most evaluation metrics, reflecting its limited effectiveness in short-response tasks. GPT-2 exhibits strengths similar to mBART in terms of Perplexity and BERTScore but shows narrower performance in traditional metrics like BLEU and METEOR, suggesting a relative advantage in contextual consistency but weaker linguistic accuracy. Meanwhile, although LLaMA and mT5 perform notably well on traditional metrics, particularly BLEU and ROUGE-L, they display lower scores on contextual criteria, with LLaMA also having notably inefficient inference times, reflecting prolonged response generation durations.

### Quantitative results of fine-tuning

This study used a specially designed dataset to enhance short question–answer interactions for AI-NPCs in metaverse environments. Due to the limited size and complexity of Turkish examples, encoder-decoder models like mBART and mT5 demonstrated low accuracy and high training losses, ultimately failing to achieve adequate fine-tuning performance. Consequently, they were excluded from further evaluation. Conversely, decoder-only models (GPT-2, LLaMA, Qwen) produced more stable and operationally viable results.

These findings are supported by the fine-tuning training loss curves presented in Appendix B. The GPT-2 model shows a rapid loss reduction in the early epochs, followed by a slower and more fluctuating decline. The Qwen model exhibits a sharp initial decrease, then enters a long plateau with a gradual and stable reduction. The LLaMA model starts with a low loss value and decreases in a smooth and monotonic manner. In contrast, the mBART model begins with a very high loss but shows a sharp early drop, indicating rapid adaptation to the task. The mT5-Small model starts with a high loss and decreases steadily over a long period; however, its convergence at a higher loss level suggests limited model capacity for learning complex contextual relations. The flattening of the loss curves in encoder–decoder models indicates early convergence and restricted learning gains. Large and modern decoder-only models (Qwen and LLaMA) show more stable learning with lower variance. Encoder–decoder models (mBART and mT5) adapt quickly at the beginning, but their final performance is more sensitive to data size, model capacity, and training strategy. Despite its older architecture, GPT-2 demonstrates meaningful learning when trained with limited data and appropriate hyperparameters.

Comparative fine-tuning performance analyses for these models, including specific evaluation metrics, are presented in Table 9.

**Table 9:** Conventional Fine-tuning based generation performance results.

Model Structure	Model	Perplexity Score	Layer-Freezing Perform	Evaluation Metric Scores			Inference Time (s)
				BLEU	METEOR	ROUGE-L	
Decoder-only	GPT2	15.29	0.044	0.207	0.377	0.278	1.086
	LLAMA	7.59	0.146	0.152	0.254	0.190	2.232
	QWEN	6.81	0.122	0.270	0.178	0.131	0.398

As shown in Table 9, the performance evaluation of decoder-only models trained via fine-tuning yielded generally lower scores compared to RAG-based approaches. Notably, the GPT-2 model exhibited a very high perplexity score $\left(GPT-{2}_{Perp.}=15.29\right)$, indicating weakened language prediction capabilities and statistically inconsistent outputs. Although the LLaMA and Qwen models demonstrated relatively lower perplexity values $\left(LLaM{A}_{Perp.}=7.59, Qwe{n}_{Perp.}=6.81\right)$, reflecting more controlled text generation than GPT-2, these values still fell short of the results achieved through RAG-based training. From an evaluation metrics perspective, the Qwen model showed a notable improvement in BLEU score $\left({Qwen}_{BLEU}=0.270\right)$ compared to the other models. However, it underperformed in METEOR $\left({Qwen}_{METEOR}=0.178\right)$ and $\left({Qwen}_{ROUGE-L}=0.131\right)$, suggesting that while the model can generate surface-level similar expressions, it struggles with maintaining semantic coherence and sequential flow. The GPT-2 model offered a relatively balanced performance with $\left(GPT-{2}_{BLEU}=0.207\right)$ and METEOR $\left(GPT-{2}_{METEOR}=0.377\right)$ scores. Nevertheless, its high perplexity and low layer-freezing performance $\left(GPT-{2}_{L.F.Perf.}=0.044\right)$ indicate a strong dependency on the training process. LLaMA, on the other hand, recorded the highest inference time $\left(Llam{a}_{I.Time}=2.23 sec.\right)$ rendering it inefficient for real-time applications. The contextual fine-tuning results of these models are presented in Table 10.

**Table 10:** Contextual fine-tuning generation performance results.

Model Structure	Model	BERTScore	BLEURT	DialogRPT
Decoder-only	GPT-2	0.518	0.547	0.417
	LLaMA	0.345	0.201	0.092
	Qwen	0.367	0.247	0.358

**Fig. 12:**
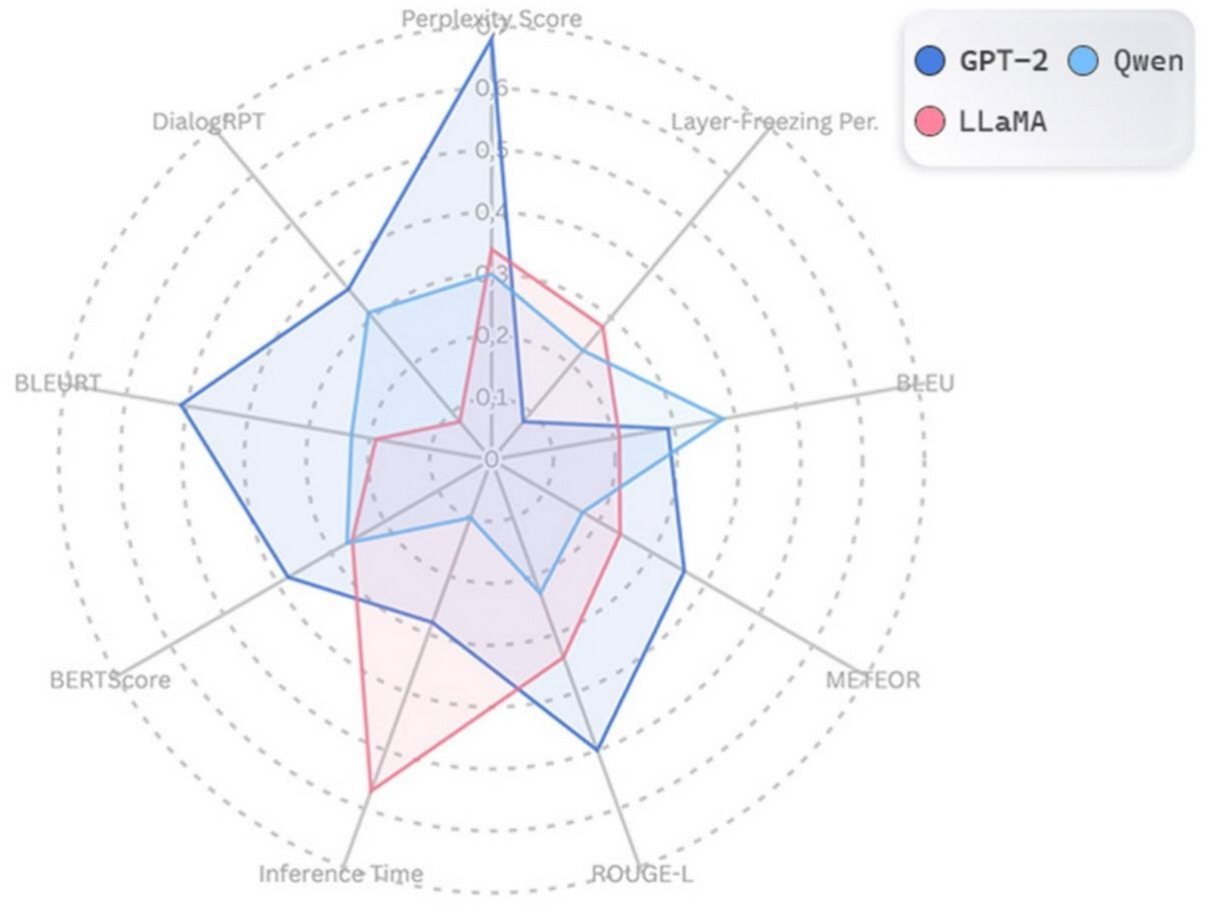
Comparative metrics analysis of LLMsFine-tuning.

The results presented in Table 10 highlight the contextual quality performance of decoder-only models trained using the fine-tuning approach. The GPT-2 model achieved strong results in both BERTScore $\left(GPT-{2}_{BERTscore }=0.518\right)$ and $\left(GPT-{2}_{BLEURT }=0.547\right)$ , indicating a high degree of semantic similarity between its outputs and reference texts, as well as alignment with human judgment. Additionally, GPT-2’s DialogRPT score $\left(GPT-{2}_{DialogRPT}=0.417\right)$ suggests a strong potential for user engagement. In contrast, the LLaMA model recorded the lowest performance across all three metrics $(LLaMA\_(BERTscore )=0.345), (LLaMA\_(BLEURT )=0.201),(Llama\_(DialogRPT )=0.092)$, indicating a deficiency in generating contextually appropriate responses, both in terms of semantic alignment and user interest. The notably low DialogRPT score reflects the model’s inability to sustain natural conversational flow. While Qwen showed moderate scores in BERTScore $\left(Qwe{n}_{BERTscore }=0.367\right)$ and $\left(Qwe{n}_{BLEURT }=0.247\right)$, it stood out with a relatively strong DialogRPT score $\left(Qwe{n}_{DialogueRPT }=0.358\right)$. This suggests that although its outputs may lack semantic depth or refined quality, they still possess characteristics that can engage users. The comparative performance analysis of the three decoder-only LLMs (GPT-2, LLaMA, Qwen) in fine-tuning is visualized in the radar chart presented in Fig. 12. The metrics included in the chart have been standardized using the TOPSIS mean calculation method to ensure cross-model comparability.

Figure 12 presents a comparative analysis of the performance metrics for three different LLMs (GPT-2, LLaMA and Qwen) after the fine-tuning process. According to the results, GPT-2 performed strongly in the contextual and language modeling dimensions, especially in metrics such as Perplexity, BLEURT, BERTScore and DialogRPT. This suggests that GPT-2 has a relatively higher capacity to produce linguistically and semantically consistent outputs during fine-tuning. Although the LLaMA model seems to have an advantage in terms of extraction time, it scores lower in overall language production quality, especially in BLEURT and BLEU. This suggests a trade-off where LLaMA offers processing efficiency at the expense of textual quality. Qwen, on the other hand, showed a balanced but limited performance profile. The model achieved moderate results across all metrics, offering a consistent but not exceptional structure in terms of both computational efficiency and linguistic consistency.

Overall, these findings suggest that the fine-tuning method provides only limited effectiveness when applied to models with small and task-oriented datasets. This reinforces the idea that RAG-based approaches that integrate richer contextual information offer more effective knowledge transfer and response generation. Furthermore, the observed low layer freezing performance scores suggest that fine-tuned models require more structural parameter updates, leading to increased demands on computational resources and training time.

### Comparative evaluation of fine-tuned vs. RAG-enhanced models

This section presents a performance comparison of the models developed using fine-tuning and RAG techniques. The scores obtained from all evaluation metrics were normalized using the TOPSIS method to enhance comparability across models. The resulting comparative findings are detailed in Table 11.

**Table 11:** Normalized TOPSIS scores for RAG-based and Fine-tuned models.

Technique	Model Structure	Model	Perplexity Score	Layer-Freezing Per	BLEU	METEOR	ROUGE-L	Inference Time	BERTScore	BLEURT	DialogRPT
RAG	Decoder-only	GPT-2	0,32	0,28	0,26	0,40	0,33	0,28	0,44	0,37	0,36
		LLaMA	0,21	0,42	0,16	0,37	0,40	0,57	0,38	0,27	0,30
		Qwen	0,41	0,28	0,18	0,25	0,26	0,15	0,25	0,25	0,34
	Encoder-Decoder	mBART*	0,11	0,52	0,50	0,52	0,37	0,33	0,43	0,55	0,56
		mT5*	0,11	0,51	0,59	0,40	0,33	0,23	0,35	0,29	0,36
Fine-tuning	Decoder-only	GPT-2	0,68	0,08	0,29	0,36	0,50	0,28	0,38	0,51	0,36
		LLaMA	0,34	0,28	0,21	0,24	0,34	0,57	0,26	0,19	0,08
		Qwen	0,30	0,23	0,38	0,17	0,23	0,10	0,27	0,23	0,31

*Fine-tuning results for mBART and mT5 could not be obtained.

As shown in Table 11, the encoder-decoder models mBART and mT5 exhibit high performance when used with the RAG technique. The mBART model stands out in terms of both linguistic similarity and dialogue quality, achieving strong results in METEOR (0.52), ROUGE-L (0.37), BLEURT (0.55), and DialogRPT (0.56). Meanwhile, mT5 attained the highest BLEU score (0.59), indicating superior accuracy in knowledge-based generation. In contrast, decoder-only models yielded comparatively weaker results under RAG-based scenarios. For example, while GPT-2 underperformed in inference time (0.28), it delivered above-average results in content quality metrics such as BLEURT (0.37) and BERTScore (0.44). The LLaMA model achieved the highest inference efficiency with a score of 0.57, making it the most computationally efficient among the RAG-based variants.

As a significant methodological limitation, the fine-tuned variants of the encoder-decoder models mBART and mT5 could not yield valid results. The dataset employed in this study was designed to enhance the capabilities of AI-NPCs in generating short, context-specific responses in Turkish within metaverse environments. However, the limited quantity and diversity of Turkish language examples posed training challenges during the fine-tuning process. This issue led to high training loss and low accuracy rates, particularly in the encoder-decoder models mBART and mT5. Consequently, only the RAG-based versions of these models were included in the comparative analyses. The comparative analysis results of both RAG and fine-tuning techniques are summarized in Table 12.

**Table 12:** Comparative overall performance of RAG and Fine-tuning techniques.

Model	Technique	Model Architecture	TOPSIS Avg. Score (Approx.)	Overall Performance Evaluation
mBART	RAG	Encoder-Decoder	~ 0.652	Demonstrates the most balanced and high-level performance among the evaluated models
mT5	RAG	Encoder-Decoder	~ 0.555	Exhibits strong results in conventional metrics and moderate performance in contextual evaluations
GPT-2	Fine-tuning	Decoder-only	~ 0.404	Performs effectively on contextual metrics; however, its overall reliability is limited due to high perplexity
GPT-2	RAG	Decoder-only	~ 0.390	Shows contextual advantages but yields moderate results in conventional evaluations
LLaMA	RAG	Decoder-only	~ 0.334	Offers efficient inference time but overall performance remains low
LLaMA	Fine-tuning	Decoder-only	~ 0.287	Despite its speed, it lacks semantic coherence and overall adequacy
Qwen	RAG	Decoder-only	~ 0.293	Displays balanced performance, though its overall effectiveness is low
Qwen	Fine-tuning	Decoder-only	~ 0.268	Presents a balanced yet weak profile, delivering limited and low-level performance

According to the normalized TOPSIS scores presented in Table 12, the RAG technique demonstrates more balanced and higher overall performance, particularly when applied with encoder-decoder models such as mBART and mT5. The mBART model achieved the highest average score (~ 0.652), while the mT5 model exhibited strong performance in conventional metrics and moderate performance in contextual metrics. Although the decoder-only GPT-2 model gained contextual advantages under the RAG, its reliability remained limited under fine-tuning due to its high perplexity, despite strong results in contextual metrics. The LLaMA model proved efficient in inference time across both techniques, but its linguistic coherence remained weak. Similarly, the Qwen model maintained a balanced yet low-performance profile under both RAG and fine-tuning configurations.

**Fig. 13:**
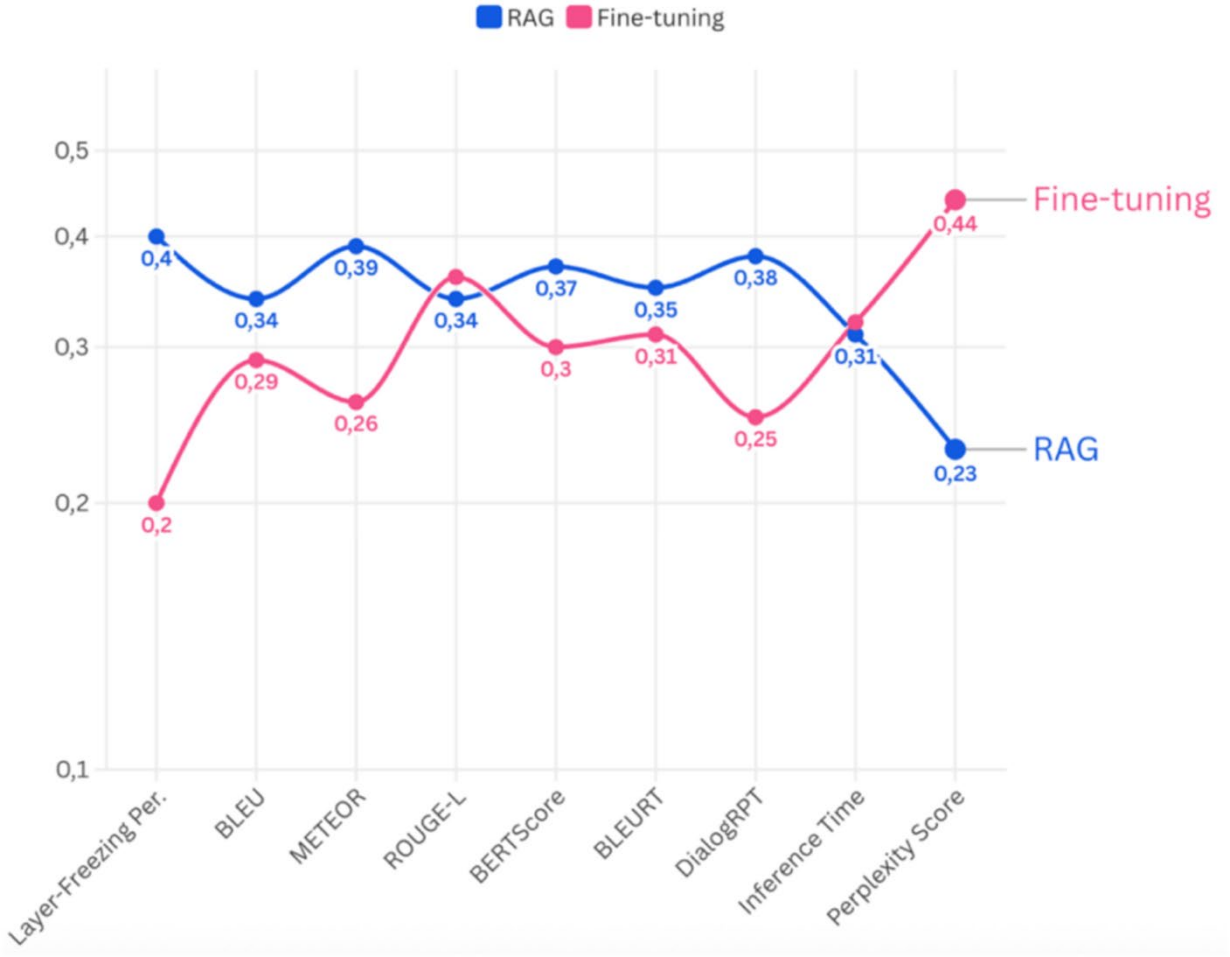
Comparative metrics of RAG and fine-tuning.

These findings highlight the superiority of the RAG in delivering deeper contextual understanding and greater textual coherence. Visual comparison of RAG and fine-tuning metrics is illustrated in Fig. 13.

As illustrated in Fig. 13, the RAG generally demonstrates a more balanced and stable performance profile, whereas fine-tuning exhibits notable spikes in specific metrics. These discrepancies suggest that the choice of method should be guided by the nature of the target tasks. Table 13 presents a comprehensive evaluation of the best-performing models across all metrics.

**Table 13:** Comparative overall performance of RAG and Fine-tuning techniques.

Metric	RAG Best Score	Fine-Tuning Best Score	Comparison
Perplexity Score*	mBART (0.11)	LlaMA (0.21)	RAG models yield lower uncertainty in terms of language model prediction and naturalness
Layer-Freezing Perf	mBART (0.52)	LLaMA (0.28)	RAG demonstrates better transfer learning and adaptation capabilities
BLEU	mT5 (0.59)	Qwen (0.38)	RAG models better align with the reference texts
METEOR	mBART (0.52)	GPT-2 (0.36)	mBART shows stronger performance in stem similarity
ROUGE-L	LLaMA (0.40)	GPT-2 (0.50)	GPT-2 in fine-tuning stands out in terms of structural similarity, especially in long texts
Inference Time*	Qwen (0.15)	Qwen (0.15)	Qwen produced fast responses in both techniques
BERTScore	GPT-2 (0.44)	GPT-2 (0.38)	RAG is more effective; GPT-2 excels in contextual alignment
BLEURT	mBART (0.55)	GPT-2 (0.51)	RAG provides responses closer to human judgment
DialogRPT	mBART (0.56)	GPT-2 (0.36)	mBART performed better in dialogue response quality

* Lower scores are more meaningful for Perplexity Score and Inference Time metrics.

**Fig. 14:**
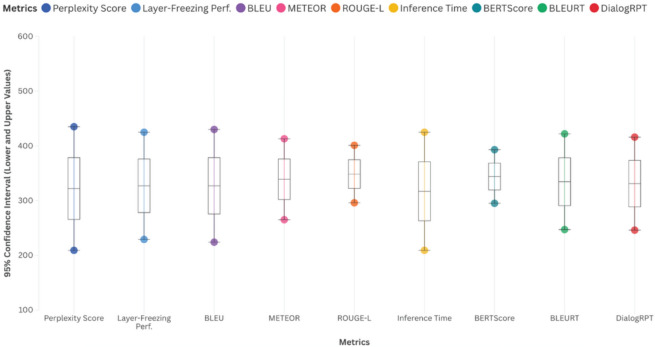
Bootstrap 95% confidence interval results.

According to the overall findings of the comparative evaluation presented in Table 13, the RAG technique outperforms the fine-tuning method in several key metrics, including Perplexity, BLEU, METEOR, BLEURT, and DialogRPT. These results indicate that RAG operates with lower uncertainty, offers higher linguistic accuracy than fine-tuning, and demonstrates superior contextual coherence. Furthermore, Table 12 supports these results. Both techniques, the Qwen model demonstrated the fastest inference time. Overall, RAG offers a more balanced and effective approach in terms of contextual depth and semantic coherence. This observation is further supported by the 95% confidence interval graph derived from bootstrap analysis, as illustrated in Fig. 14.

As illustrated in Fig. 14, 95% bootstrap confidence interval was applied across all evaluation metrics. This non-parametric method is commonly used in statistical analysis to assess the reliability of results, particularly when sample sizes are limited or when the assumptions of parametric tests (e.g., t-tests) are not met. The graph presents 95% bootstrap confidence intervals, offering critical insights into the statistical robustness of the evaluated metrics. Upon examining the chart, it becomes evident that metrics such as ROUGE-L, DialogRPT, and BLEURT exhibit relatively narrow confidence intervals, indicating that they serve as more stable and reliable indicators of model performance. In contrast, the wide confidence intervals observed for metrics like Perplexity Score and Inference Time suggest high variability across models, implying that these metrics may be less suitable as standalone decision-making criteria. The graph highlights the importance of considering not only average scores in model comparisons, but also the associated confidence intervals to ensure more robust and informed evaluations.

### Ablation study

In this section, ablation study is performed to analyze how individual training strategies and RAG contribute to performance variations in short Turkish question–answering tasks. Table 14 shows the results of the fine-tuning ablation study.

**Table 14:** Fine-tuning ablation study results.

Model	Model Architecture	Initial Loss	Final Loss	Relative Improvement	Early Slope	Loss Volatility
GPT-2	Decoder-only	18.215	16.117	0.8313	− 0.00044	0.039
LLaMA	Decoder-only	0.294	0.049	0.7948	− 0.00057	0.090
Qwen	Decoder-only	0.521	0.107	0.1152	− 0.00138	0.094
mBART	Encoder–Decoder	0.469	0.379	0.7260	− 0.00951	0.779
mT5	Encoder–Decoder	21.513	5.767	0.1900	− 0.02241	0.439

Table 14 presents the results of the fine-tuning ablation study. Encoder–decoder models (mBART and mT5) exhibit faster early adaptation and larger relative loss reductions, with mBART achieving the highest improvement; however, these gains are accompanied by substantially higher loss volatility, indicating less stable optimization. In contrast, decoder-only models (GPT-2, LLaMA, and Qwen) show slower convergence and more moderate or model-dependent improvements, while consistently maintaining lower loss volatility and smoother training dynamics. Table 15 presents the results of the RAG ablation study.

**Table 15:** RAG ablation study results.

Model	Model Architecture	Initial Loss	Final Loss	Relative Improvement	Early Slope	Loss Volatility
GPT-2	Decoder-only	3.149	2.430	0.228	− 0.00050	0.038
LLaMA	Decoder-only	0.940	0.698	0.258	− 0.00057	0.090
Qwen	Decoder-only	3.096	1.618	0.478	− 0.00138	0.094
mBART	Encoder–Decoder	10.624	0.375	0.965	− 0.00959	0.781
mT5	Encoder–Decoder	27.394	5.779	0.789	− 0.02486	0.491

**Fig. 15:**
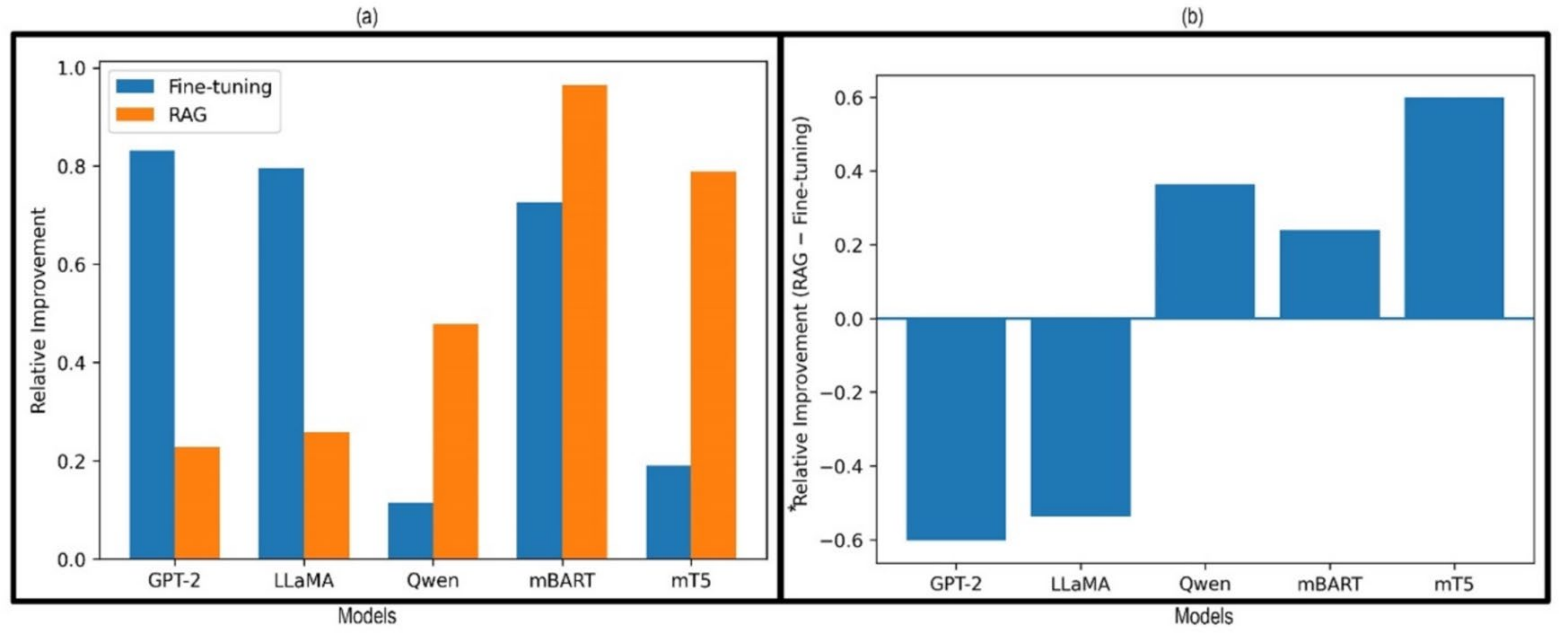
Performance comparison between RAG and fine-tuning across different model architectures: (**a**) Relative improvement by model architecture for RAG and fine-tuning, and (**b**) normalized improvement gain. (* relative improvement = RAG − fine-tuning)

Table 15 presents the ablation results of RAG. Encoder–decoder models (mBART and mT5) exhibit notably faster early convergence and substantially higher relative improvements, with mBART achieving the largest gain, indicating that retrieval augmentation is particularly effective for architectures with explicit cross-attention mechanisms. However, these gains are accompanied by significantly higher loss volatility, reflecting increased sensitivity to retrieved context and less stable optimization. In contrast, decoder-only models (GPT-2, LLaMA, and Qwen) show more moderate performance gains under RAG while consistently maintaining lower loss volatility and smoother training trajectories. Figure 15 provides an overview of the ablation study results for RAG and fine-tuning training.

Figure 15 demonstrate that the effectiveness of RAG and fine-tuning is strongly dependent on model architecture. As shown in Fig. 15a, fine-tuning yields higher relative improvements for certain decoder-only models (GPT-2 and LLaMA), whereas RAG provides substantially larger gains for encoder–decoder architectures (mBART and mT5) and for Qwen. This trend is further clarified in Fig. 15b through the normalized improvement gain analysis, which highlights a clear strategy preference: fine-tuning is more advantageous for some decoder-only models, while RAG consistently outperforms fine-tuning in encoder–decoder models . These results indicate that the choice between RAG and fine-tuning should be guided by architectural characteristics rather than treated as a universally optimal strategy.

### Human results vs. generated results

This section presents a comparative analysis between human-generated responses and LLM-generated outputs obtained through fine-tuning and RAG. The comparison results are reported in Table 16.

**Table 16:** Comparison bbetween human responses and LLMs outputs (%).

Model	Training Strategy	Accuracy vs. Human (%)
mT5	RAG	86%
mBART	RAG	78%
LLaMA	RAG	32%
GPT-2	Fine-tuning	29%
GPT-2	RAG	25%
LLaMA	Fine-tuning	22%
Qwen	RAG	17%
Qwen	Fine-tuning	15%

As shown in Table 16, mT5-RAG achieves the closest alignment with human evaluations. RAG integration consistently reduces hallucination risk and enhances contextual relevance across all models. However, decoder-only architectures (GPT-2, LLaMA, and Qwen) remain significantly below human performance even when augmented with RAG. In addition, the human evaluation scores for the mT5 and mBART models were obtained from six expert evaluators with diverse professional backgrounds. Each evaluator rated the responses using a 5-point likert scale (1–5) based on predefined criteria. In Table 16, arithmetic means were calculated for each criterion and subsequently converted into percentages for comparative analysis. Results show that the the human evaluation results demonstrate strong performance in contextual relevance (91%) and role consistency (88%), indicating reliable alignment with conversational intent. High scores in naturalness (82%) and conversational flow (85%) further confirm compliance with everyday dialogue standards, while the overall immersion score (89%) establishes human responses as a robust reference baseline.

 , 

## Conclusion

In recent years, the metaverse has emerged as a new digital space that enables realistic interactions between users and AI-powered characters. In this immersive environment, communication between users and AI-powered NPCs significantly shapes the overall user experience. Among the various interaction modalities, voice serves as the primary medium that plays a crucial role in reinforcing the sense of presence and realism in the metaverse. Leveraging advanced speech processing technologies, AI-NPCs are able to establish natural and coherent dialogues that closely mimic real-life human communication. These agents can initiate conversations, perform set tasks and actively participate in interactive scenarios. Accordingly, ensuring that dialogs between users and AI-NPCs remain concise, context- based, and task-oriented is crucial for maintaining effective and immersive interactions.

### Theoretical implications

First, existing research shows that users mainly interact with AI-NPCs through API-based solutions^25,29,50,55,66,87^. Experimental findings show several challenges in scenarios where LLMs interact directly via APIs. Most notably, the loss of conversational context often results in AI-NPCs producing unnecessarily long, semantically inconsistent or irrelevant responses. These limitations negatively impact user engagement, reducing both satisfaction and interaction efficiency. To enable effective communication with AI-NPCs, it is crucial that responses are short, context-sensitive and task-oriented. To meet this need, developers are encouraged to adapt their preferred LLMs using RAG and fine-tuning techniques based on specialized datasets. These models offer practical and efficient solutions, especially in applications that require short question–answer interactions.

The performance differences observed among the evaluated Turkish LLMs primarily arise from the interaction between model architecture, contextual grounding strategy, and the morphological characteristics of the Turkish language^61,70,80^. Short, task-oriented conversational scenarios require not only fluent text generation but also high levels of contextual precision and semantic stability. Within this setting, RAG consistently outperforms conventional fine-tuning techniques ^13,60^. By conditioning generation on dynamically retrieved external context at inference time, RAG reduces semantic ambiguity and contextual drift that commonly occur in short and information-dense queries. This advantage is reflected in higher scores on context-sensitive metrics such as BLEURT, METEOR, and DialogRPT, indicating improved semantic alignment and discourse coherence.

From an architectural perspective, encoder–decoder models such as mBART and mT5 benefit from a structural separation between context encoding and response generation, enabling more controlled and stable outputs. This design is particularly effective for morphologically rich and agglutinative languages like Turkish. In contrast, decoder-only models, while offering faster inference, exhibit weaker contextual consistency and semantic alignment, especially when trained solely through fine-tuning. These findings indicate that encoder–decoder architectures combined with RAG provide a more balanced and reliable solution for short, context-sensitive conversational tasks.

Secondly, the fine-tuning requires access to broader and more diverse datasets in order to enhance the model’s generalization capability. This requirement is also evident in the study conducted by some studies [^66^]. Similarly, in our experiments, expanding the dataset by at least threefold was necessary to mitigate the risk of overfitting during model training. In contrast, studies utilizing the RAG technique have demonstrated that effective responses can be generated even with relatively small datasets^42^. However, the implementation of RAG-based systems has raised concerns regarding security^22^. Therefore, future research should include evaluations of the security and reliability of both RAG-enhanced models and fine-tuning approaches.

Third, traditional evaluation metrics such as BLEU, ROUGE-L and METEOR are generally effective for evaluating long text generation scenarios^40^. However, in situations requiring the evaluation of short, context-sensitive outputs, the use of more advanced context-sensitive metrics such as BLEURT, BERTScore and DialogRPT has proven to be more beneficial. In this study, these modern metrics are used to assess the semantic relevance, user engagement and discourse coherence of model outputs. Accordingly, the findings emphasize the importance of incorporating these advanced evaluation metrics in communication environments such as the metaverse, where brevity and contextual sensitivity are crucial. Future research could benefit developers by categorizing and refining these metrics to better suit a variety of interactive and productive tasks.

### Summary of findings

The findings of the study indicate that Turkish LLMs, including GPT-2, LLaMA, Qwen, mBART, and mT5, were trained using fine-tuning and RAG techniques. Their performance was subsequently evaluated based on a set of quantitative metrics.

- Based on the empirical findings of this study, RAG should be preferred in low-resource conversational settings where training data are limited, domain knowledge changes frequently, or high contextual accuracy is required. RAG enables models to leverage external knowledge at inference time, reducing the need for large-scale fine-tuning while improving semantic alignment and response relevance. In contrast, fine-tuning remains a viable option when computational resources are severely constrained, ultra-low latency is critical, and the target domain is narrow and stable. In such cases, fine-tuned decoder-only models can provide faster response generation, albeit at the cost of reduced contextual robustness. Therefore, practitioners should select RAG or fine-tuning by balancing contextual accuracy requirements against available computational resources and real-time performance constraints.
- The evaluation results reveal that the RAG technique outperforms fine-tuning on multiple evaluation metrics. In particular, higher scores on the perplexity, BLEU, METEOR, BLEURT and DialogRPT metrics demonstrate RAG’s superiority in reducing language model ambiguity and increasing contextual accuracy. This performance confirms that RAG is an effective method for producing concise and task-oriented responses, especially in scenarios requiring high contextual precision and target alignment.
- The decoder-only models trained with the fine-tuning technique showed lower overall performance compared to the RAG-based techniques. The fine-tuned technique achieved relatively higher scores only in Perplexity (0.44), which reflects prediction fluency, and inference time (0.31), which indicates faster response generation. However, mismatches between tokenizer structures and morphological features of suffixed languages such as Turkish limited the effectiveness of this method. These findings suggest that fine-tuning can provide an advantage in processing speed under certain conditions but tends to produce weaker responses in terms of contextual consistency and semantic precision.
- The mBART model, which adopts an encoder-decoder architecture, exhibited notable performance when integrated with the RAG technique. Achieving the lowest perplexity score (2.48) among all evaluated models underscores its stability in language prediction. Furthermore, high scores in METEOR (0.549) and DialogRPT (0.648) metrics highlight the model’s ability to generate semantically rich and user-engaging responses. These results position mBART as a strong candidate for short and context-sensitive NLP tasks.
- The GPT-2 model, running on a decoder-only architecture, showed remarkable success in contextual evaluation metrics when trained through fine-tuning. In particular, the model achieved strong scores on BLEURT (0.547) and BERTScore (0.518), demonstrating its capacity to produce semantically coherent and human-compatible responses. However, the high disfluency score (15.29) indicates inconsistencies in fluency and linguistic reliability. These findings imply that the GPT-2 can be effective in tasks requiring semantic depth, but its use in context-rich scenarios requires careful optimization to ensure response stability and consistency.
- Only the decoder architectures, Qwen and LLaMA, exhibited overall poor performance in both fine-tuning and RAG-based techniques. While the Qwen model achieved the fastest extraction time (0.398 s), its performance on the contextual metrics fell short, with scores of BLEURT (0.247) and DialogRPT (0.394) reflecting limited semantic competence. In contrast, the LLaMA model was not suitable for real-time applications, showing one of the slowest inference times (2.232 s) and poor contextual alignment as indicated by the low DialogRPT score (0.092). Although both models performed relatively better on surface-level metrics such as ROUGE-L, this did not translate into deeper contextual quality, highlighting limitations in their ability to support semantically rich interactions.
- According to TOPSIS scores, the RAG technique shows more balanced and higher performance, especially when applied to encoder-decoder architectures such as mBART (~ 0.652) and mT5 (~ 0.555). The mT5 model achieved the highest BLEU score (0.418), showing strong performance in producing short, task-oriented responses. These findings suggest that encoder-decoder architectures are particularly advantageous for tasks that require concise and target-specific text generation, strengthening their suitability for context-sensitive NLP applications.
- Conventional evaluation metrics such as BLEU, ROUGE-L and METEOR are often insufficient for assessing semantic quality and contextual alignment in short text scenarios. In this study, modern context-sensitive metrics, including BLEURT, BERTScore, and DialogRPT, are used to assess semantic relevance, user engagement, and discourse coherence of model outputs. The findings therefore highlight the importance of adopting these advanced metrics, especially in communication environments such as the metaverse where brevity and contextual sensitivity are critical. It is therefore recommended that next-generation evaluation frameworks prioritize context-sensitive metrics over traditional metrics in similar applications.

Overall, the findings show that encoder-decoder models such as mBART and mT5, when combined with the RAG technique, offer significant advantages for producing short and task-oriented responses. However, in application scenarios where response time is a critical factor, such as real-time interactive systems, models such as Qwen, which offer faster but more superficial outputs, can serve as suitable alternatives. These results emphasize the need to strike a balance between contextual accuracy and computational efficiency when selecting models according to the specific demands of the use case.

### Implications for future metaverse applications

In metaverse environments, natural and effective communication between users and AI-NPCs is possible with the integration of NLP technologies. In particular, voice-based interaction plays a key role in enhancing the realism of virtual experiences and strengthening the sense of presence. In this context, the implementation of STT and TTS systems is a fundamental requirement in metaverse platforms. Using advanced LLMs, natural language input provided by the user is subjected to semantic interpretation and then context-appropriate responses are generated. As a result, users equipped with VR headsets and controllers can engage in real-time, voice-based conversations with AI-NPCs.

The study shows that the STT → LLMs → TTS architecture in virtual environment development provides a pipeline in which user speech is first converted into text, a meaningful response is generated from this text, and finally the output is communicated back to the user verbally. This architecture transforms the metaverse experience into a communication system that is not only interactive, but also personalized, context-aware and real-time. Besides these benefits, STT and TTS processes are usually executed through external APIs. However, structural challenges arise in this setup. In particular, the implementation of STT and TTS transformations through external APIs often causes latency, which compromises the principle of real-time interaction. Therefore, configuring game development engines (e.g. unity or unreal engine) to natively support these conversions is crucial to ensure optimal timing and performance.

This study specifically aimed to establish a brief but meaningful communication between users and AI-NPCs in the metaverse. However, future research is expected to go beyond predefined or pre-trained models to explore affective computing, especially emotion recognition, to enrich the interaction between users and virtual agents. Ultimately, this system architecture enables metaverse platforms to evolve into environments that support not only visual and auditory interaction, but also semantic and emotional interaction. Such an approach enhances the user experience and contributes to the development of metaverse environments as multidimensional and responsive systems.

### Challenges in adapting LLMs for real-time applications

The Turkish dataset used in this study negatively affected model performance due to its limited content diversity and short sample lengths. Especially tokenizer mismatches, which are commonly observed in suffixed languages such as Turkish, limited the contextual accuracy of the fine-tuned technique, resulting in low accuracy scores and high training loss values. Encoder-decoder models such as mBART and mT5, which require contextual processing on both the input and output side, could not provide sufficient learning under these data constraints and were therefore excluded from the evaluation. In contrast, decoder-only models such as GPT-2, LLaMA and Qwen, which have fewer parameters and handle context only on the decoding side, produced more stable and consistent results under limited data conditions.

This study encountered several methodological and technical challenges during the training of fine-tuning and RAG-based language models. One of the primary challenges was achieving stable optimization for encoder–decoder models, which requires careful coordination between encoder and decoder updates, particularly in low-resource and small-batch training settings. This issue was addressed by expanding the dataset. Another major challenge involved detecting and preventing silent training failures, such as zero-loss scenarios caused by misconfigured labels or data collators, which do not produce explicit runtime errors but invalidate the learning process. In addition, RAG introduced further complexity by coupling retrieval quality with generation performance, making the models highly sensitive to context length, retrieval noise, and retriever–generator alignment. The strategies developed to mitigate the challenges and errors identified during the training process are presented in detail in Table 17.

**Table 17:** Training challenges and error mitigation strategies.

Challenge	Observed Issue	Mitigation Strategy
Optimization instability in encoder–decoder models	High initial loss and gradient fluctuations, especially in early training stages	Use lower learning rates, apply gradient clipping, and introduce warm-up steps for stable convergence
Silent training failures (loss = 0)	Learning does not occur despite the absence of runtime errors due to label or data collator misconfiguration	Validate label tensors before training, verify padding masks (-100), and monitor loss values from the first training steps
Sensitivity to data–model–loss pipeline	Minor configuration errors invalidate the entire training process	Implement automated pipeline checks and sanity tests with small data subsets
Increased complexity in RAG training	Loss variability caused by retrieval noise and heterogeneous contexts	Improve retriever quality, apply context filtering, and limit maximum context length
Retriever–generator misalignment	Retrieved documents do not support the generation objective	Perform retriever–generator alignment tuning and task-aware retrieval
Resource constraints (small batch sizes, limited data)	Reduced training stability and slower convergence	Use gradient accumulation, mixed precision training (FP16), and effective batch size scaling

As shown in table 17, overcoming these challenges required comprehensive diagnostics, iterative pipeline validation, and architecture-specific tuning strategies, underscoring the practical difficulty of deploying encoder–decoder models and RAG-based techniques in constrained experimental environments. In addition, the RAG technique inherently depends on a large and diverse document pool, as it leverages external knowledge sources to support the base model. However, in this study, the datasets used within the RAG pipeline consisted of a limited number of documents. Despite achieving low training loss values, this limitation led to overfitting and restricted the model’s generalization capability. Therefore, for more reliable performance evaluation, training RAG-based techniqueon comprehensive, diversified, and task-oriented datasets is recommended. Overall, the findings indicate that both model architecture and dataset characteristics —particularly data quality, coverage, and linguistic structure—play a critical role in determining model performance. Therefore, data quality and dataset adequacy should be prioritized in model customization studies for under-resourced languages such as Turkish.

Furthermore, one of the main technical challenges in voice-based interaction architectures is the handling of STT and TTS transformations. Converting user speech to text, generating meaningful responses through LLMs, and converting the output back to speech extends the communication process. Since these transformations are usually executed through external APIs, they cause transmission delays and undermine the principle of real-time interaction. Therefore, integrating these processes directly into game engines such as unity or unreal engine will reduce processing time and contribute to smoother and more natural interactions 

## Discussion

Recent studies have systematically examined the trade-offs between fine-tuning and RAG for question answering tasks. The literature consistently shows that fine-tuning is particularly effective in short-context, closed-domain, and domain-specific settings due to its low inference latency and reliance on parametric knowledge representations^11,64,65,82^. However, this reliance on static internal knowledge constrains its applicability in scenarios requiring frequent knowledge updates or complex reasoning across multiple sources^43,62^.

In contrast, RAG technique have become the dominant approach for knowledge-intensive and multi-hop reasoning tasks. Prior work demonstrates that RAG-based models consistently outperform fine-tuned counterparts on benchmarks such as HotpotQA, 2WikiMultiHopQA, and StrategyQA, where answers require evidence aggregation and reasoning over multiple documents^27,84^. The ability to retrieve external and up-to-date information improves factual grounding and reduces hallucinations, although performance remains highly sensitive to retrieval quality^41,63^

Our findings closely mirror these established trends. Fine-tuned technique achieve competitive performance on single-hop datasets such as natural questions, whereas RAG-based approaches provide substantial gains on multi-hop and commonsense reasoning benchmarks. This confirms that reasoning depth and knowledge intensity are decisive factors in determining the relative effectiveness of fine-tuning versus RAG.

Moreover, our results align with recent shifts toward adaptive retrieval strategies. Dynamic RAG variants, which trigger retrieval based on uncertainty or semantic signals, demonstrate a more favorable balance between retrieval efficiency and reasoning accuracy than static retrieval techniques^23,76^. This observation reinforces the growing consensus that fine-tuning and RAG should be viewed as complementary rather than competing paradigms: fine-tuning remains suitable for low-latency, domain-specific tasks, while RAG is better suited for knowledge-intensive and multi-hop reasoning scenarios requiring explicit evidence grounding.

Beyond model architecture, our analysis further highlights the importance of data formulation in RAG training. In particular, a structured question–context–answer format proves to be more effective for RAG-based learning, as it explicitly aligns retrieval outputs with generation targets. This structure is especially beneficial for short question-answering scenarios, where precise context selection directly influences response accuracy. However, these benefits come at the cost of increased computational complexity. Training RAG technique with large-scale question–context–answer datasets often requires prolonged training times and substantial hardware resources, which can pose practical challenges for large-scale experimentation and deployment. This trade-off underscores the need for more efficient training strategies and resource-aware RAG technique in future work.

## Limitations

Within the framework of the proposed metaverse-based AI-NPC architecture, both encoder-decoder and decoder-only models are integrated to enable voice-based interaction. The primary goal was to facilitate the effective operation of AI-driven characters in the Turkish language while providing natural and realistic user interaction.

However, several limitations were encountered during the implementation of the system. First, insufficient hardware resources (i.e. limited processing power and memory) and slow internet connectivity caused delays and performance degradation during both training and real-time deployment of the models. These technical limitations prevented the full evaluation of large-scale encoder-decoder LLMs.

Moreover, the limitations of the dataset negatively affected the model performance. The fact that the Turkish examples used in the training were limited in terms of both content and variety limited the generalization capabilities of the models and reduced their learning efficiency. Considering the morphologically rich structure of the Turkish language, this problem is even more pronounced. In this context, only two encoder-decoder models that offer Turkish language support (mBART and mT5) could be analyzed in this study. The exclusion of other models was due to both hardware-software incompatibilities and the lack of Turkish language support. Future studies should consider including a wider range of encoder-decoder models with Turkish language support and reporting both quantitative and qualitative evaluation results.

Finally, it is important to note that relying solely on automated metrics is insufficient to evaluate such systems. Integrating user feedback and human-based evaluations is necessary for a more comprehensive evaluation, especially in subjective aspects such as dialog naturalness, response relevance and interaction effectiveness.

## Future work

Future research is encouraged to present the results of extensive experiments with various language models and languages. In the current study, only five LLMs were examined. This limitation restricted broader comparisons in terms of both linguistic and architectural diversity. Therefore, it is critical that future work be extended to include multilingual scenarios and different model architectures.

This study focused only on a level of communication based on short and information-oriented responses. However, language models should be evaluated not only for their capacity to convey factual content, but also for their ability to detect emotional states and produce appropriate emotional responses. Accordingly, it is recommended that future research should develop models capable of emotion recognition. Such developments are expected to make significant contributions in human-centered fields such as healthcare, psychological support, elderly care and rehabilitation, where emotionally aware AI systems can play a transformative role.

Furthermore, the language models in this study were trained using only three thematic domains. However, given the multidimensional nature of real-world applications, it is important to include a variety of usage scenarios in subsequent research. Testing the interaction capabilities of AI-NPCs systems in various sectors, including education, healthcare, tourism and finance, will contribute to domain-specific optimization and customization processes. The pace of digital transformation in these sectors is expected to increase, especially as 5G technology continues to be adopted. Therefore, ensuring readiness at the infrastructure and application level will be a critical aspect of future work.

Moreover, in this study, only the FAISS retriever was used in the RAG architecture to maintain consistency. However, it is well known that different retrieval mechanisms such as BM25, DPR, Contriever or ColBERT may have different effects on the overall model performance. Including different retrieval strategies in future studies would allow for a more comprehensive assessment of how the retrieval method affects response quality. Therefore, the inclusion of alternative retrieval components in future research is highly recommended.

## Supplementary Information

Supplementary Information 1.

Supplementary Information 2.

Supplementary Information 3.

Supplementary Information 4.

Supplementary Information 5.

## Data Availability

The datasets generated and analyzed during the current study are publicly available on the Hugging Face Datasets Hub at the following repository: https://huggingface.co/datasets/ibokajordan/turkish_metaverse_eco_dialogues The dataset, entitled “Turkish Metaverse and Eco Dialogues (RAG + Fine-tuning)”, consists of Turkish question–context–answer triples developed to support Retrieval-Augmented Generation (RAG) and fine-tuning processes for large language models. It includes dialogues related to daily life, metaverse interactions, and eco-friendly communication themes. The dataset is released under the Creative Commons Attribution 4.0 International (CC BY 4.0) license and can be freely accessed and reused for research and educational purposes, provided that appropriate credit is given to the original author.

## Abbreviations

AI: Artificial intelligence
AI-NPCs: Artificial intelligence supported non-player characters
API: Application programming interface
AR: Augmented reality
BERT: Bidirectional encoder representations from transformers
BLEU: Bilingual evaluation understudy
DPR: Dense passage retriever
FAISS: Facebook AI similarity search
GAN: Generative adversarial network
GenAI: Generative artificial intelligence
LLMs: Large Language Models
LSTM: Long short-term memory
METEOR: Metric for evaluation of translation with explicit ordering
NLP: Natural language processing
NPC: Non-player character
RAG: Retrieval-augmented generation
RNN: Recurrent neural networks
ROUGE: Recall-oriented understudy for gisting evaluation
ROUGE-L: Recall-oriented understudy for gisting evaluation longest common subsequence
Seq2seq: Sequence-to-sequence
STT: Speech-to-text
TOPSIS: Technique for order preference by similarity to ideal solution
TTS: Text-to-speech
VR: Virtual reality
