# Supplementary figures and images for: Designing metaverse interaction systems for the Turkish language enhanced by fine-tuning and retrieval-augmented generation (RAG)
Source: Sci Rep. 2026 Apr 20;16:18294. doi: 10.1038/s41598-026-35392-x (PMC13261109; doi:10.1038/s41598-026-35392-x)

# Appendix A. RAG Training Loss Curves


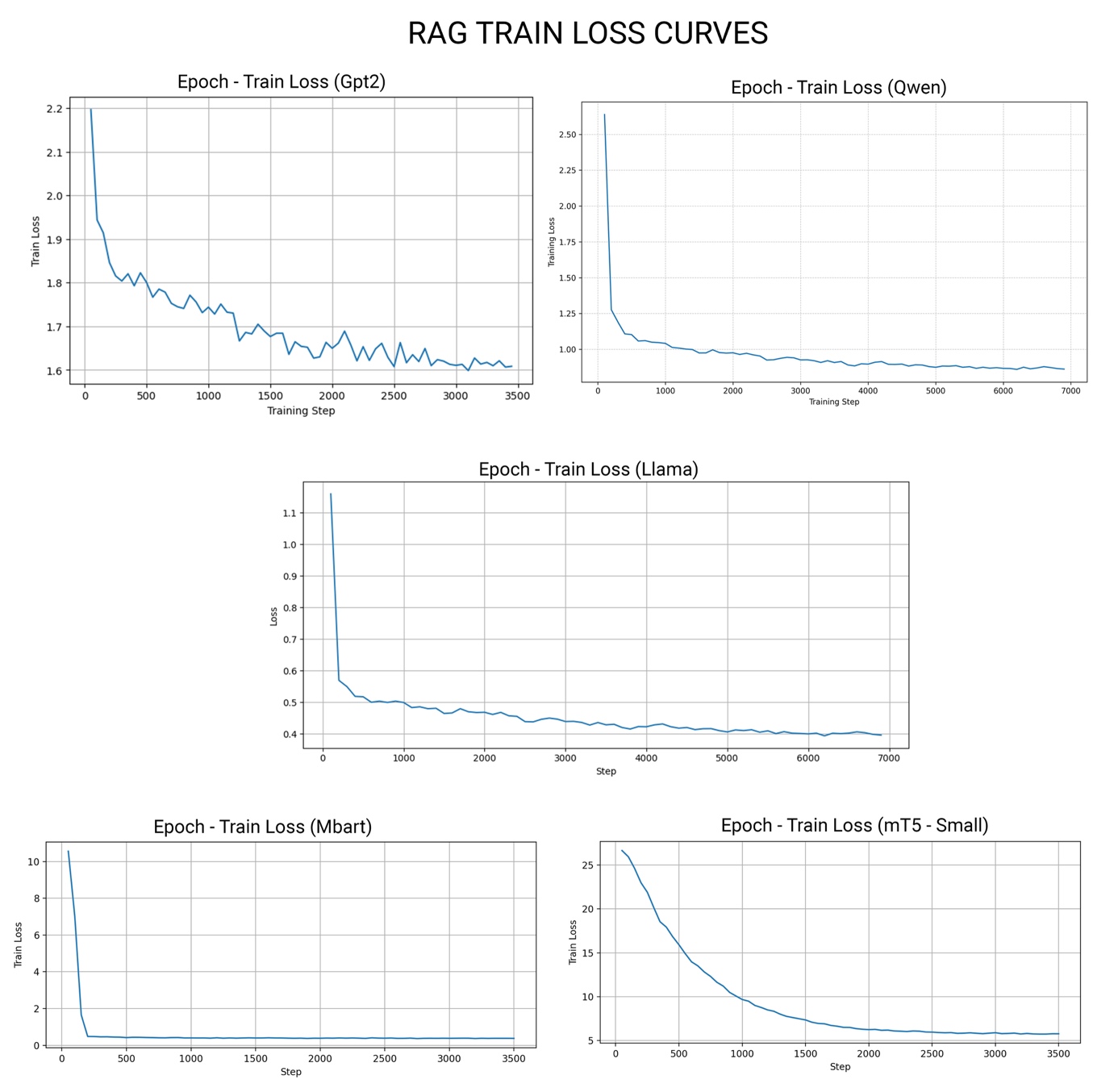

Supplement: Supplementary file 1 — Supplementary Information 1. [file 41598_2026_35392_MOESM1_ESM.docx]

# **Appendix B. Fine Tuning Training Loss Curves**


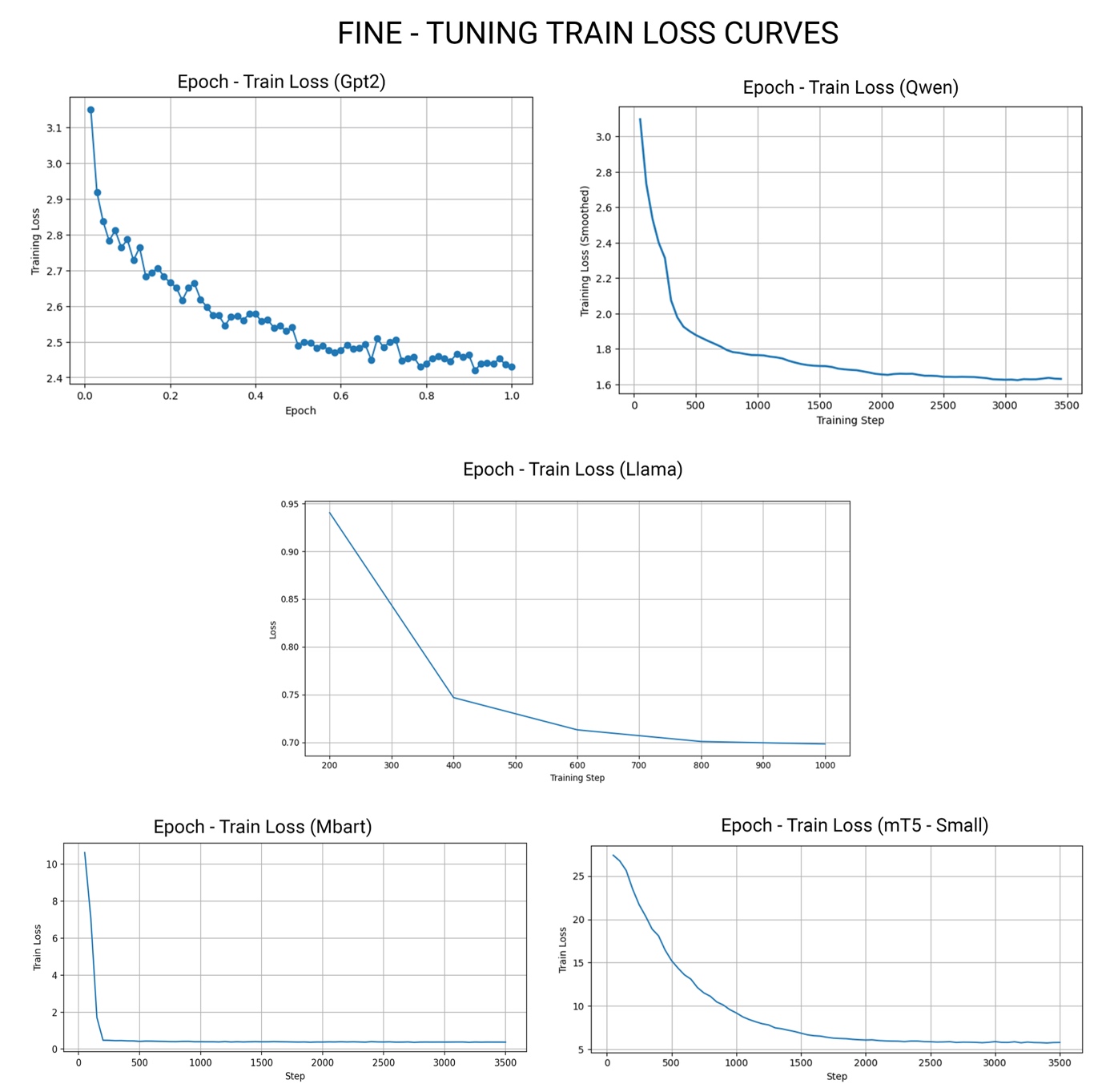

Supplement: Supplementary file 2 — Supplementary Information 2. [file 41598_2026_35392_MOESM2_ESM.docx]
