# Supplementary material for: Designing metaverse interaction systems for the Turkish language enhanced by fine-tuning and retrieval-augmented generation (RAG)
Source: Sci Rep. 2026 Apr 20;16:18294. doi: 10.1038/s41598-026-35392-x (PMC13261109; doi:10.1038/s41598-026-35392-x)
Supplement: Supplementary file 3 — Supplementary Information 3. [file 41598_2026_35392_MOESM3_ESM.docx]

# Appendix C. Evaluation metrics used in the study

This study investigates the capacity of AI-NPCs, deployed within the metaverse environment, to generate short, meaningful, and task-oriented responses to natural language queries from users. For this purpose, the developed language models were benchmarked in terms of content quality and computational efficiency. The various quantitative metrics used in the evaluation process to measure model performance are summarized in Table 5.

**Table 5.** Evaluation metrics used in the study.

| **Metric Name** | **Category** | **Primary Purpose** | **Description** |
| --- | --- | --- | --- |
| Perplexity Score | Content Quality | Measures how well a language model predicts the next word | Lower perplexity indicates more fluent and predictable text |
| Layer-Freezing Perf. | Computational Efficiency | Evaluates which layers to train during transfer learning | Aims to reduce computational cost while maintaining similar performance |
| BLEU | Content Quality | Measures n-gram overlap between generated and reference text | Common in machine translation and summarization tasks |
| METEOR | Content Quality | Improves upon BLEU by accounting for semantics and word order | Considers synonyms, stemming, and word alignment |
| ROUGE-L | Content Quality | Measures overlap between summaries and references | Uses Longest Common Subsequence (LCS) for comparison |
| Inference Time | Computational Efficiency | Measures how fast a model generates a response | Crucial for real-time applications |
| BERTScore | Semantic Quality | Measures semantic similarity between generated and reference text | Computes cosine similarity using BERT-based embeddings |
| BLEURT | Semantic Quality (Human-like) | Produces scores aligned with human judgments | Effective for evaluating natural language generation |
| DialogRPT | Interactional Relevance | Evaluates how engaging or relevant a dialogue response is | Predicts user engagement using a Reddit-trained model |

Table 5 summarizes the evaluation metrics employed for different analytical objectives within the scope of this study.

Perplexity is a foundational metric used to assess the predictive accuracy of probabilistic language models, particularly in estimating the naturalness of generated text. It quantifies the model’s difficulty in anticipating the next word in a sequence, with lower perplexity values indicating more effective language modeling (Jelinek et al., 1977). Mathematically, it is derived by calculating the exponential of the average negative log-likelihood of each word given its preceding context (Jin et al., 2024), as described in equation (1). Here, *N* represents the total number of words, and the conditional probability $P_{\left( w_{i} | w_{1-i} \right)}$ reflects the model’s confidence in its predictions. The resulting value offers an interpretable, positive score for evaluating linguistic coherence and fluency.

| $Perplexity=exp(-\frac{1}{N}\sum_{i=1}^{N} \log P_{\left( w_{i} \vert w_{1-i} \right)}$ | (1) |
| --- | --- |

Layer freezing is a training optimization technique in which specific layers of a pre-trained large language model are kept fixed during fine-tuning, meaning their weights remain unchanged. This method is particularly advantageous in scenarios with limited computational resources or small datasets, as it helps reduce training time while enhancing model performance (Figueroa, 2023). In this study, approximately 50% of the model layers were frozen, following the parameter configuration shown in equation (2). In this configuration, the model parameters *θ* are divided into two subsets: frozen layers, which remain static during training, and trainable layers, which continue to be updated. This selective training strategy provides a balanced compromise between computational efficiency and the model’s ability to adapt to new tasks.

$$\begin{aligned} \theta_{frozen}=\left[ \theta_{frozen}, \theta_{trainable} \right]\#\# \#\left( 2 \right) \end{aligned}$$

The BLEU score is a widely adopted metric for evaluating the output quality of models in tasks such as machine translation and text generation. It evaluates the agreement between model-generated and reference texts by calculating the precision of n-gram overlaps (Rivera-Trigueros, 2022). BLEU scores range from 0 to 100, with higher scores indicating closer alignment with the reference output. The metric is based on the geometric mean of n-gram match rates ${(p}_{n}$) is calculated as a weighted average of their logarithms, where the weights $(w_{n}$) is usually assigned uniformly. This logarithmic mean is then exponentiated and scaled by a brevity penalty *(BP)* to produce the final score as defined in equation (3).

$$\begin{aligned} \mathrm{BLEU}=BP.\exp\left( \sum_{n=1}^{N} {(w}_{n}\log p_{n}) \right)\#\left( 3 \right) \end{aligned}$$

The METEOR score is a semantic-oriented metric frequently used in machine translation and text generation tasks. It evaluates the similarity between candidate and reference sentences by incorporating multiple levels of comparison, including exact word matches, synonymy, stemming, and semantic interpretations. Unlike metrics that rely solely on surface-level overlap, METEOR integrates both precision and recall through their harmonic mean ${(F}_{mean}$​) and introduces a penalty term that accounts for discrepancies in word order alignment (Denkowski & Lavie, 2014). The final score is calculated by adjusting the $F_{mean}$​ value with this penalty factor, as illustrated in equation (4).

$$\begin{aligned} METEOR=F_{mean}.\left( 1-Penalty \right)\#\left( 4 \right) \end{aligned}$$

The ROUGE-L score is one of the most commonly used metrics for evaluating the performance of text summarization systems. It measures the degree of n-gram overlap between system-generated summaries and human-written reference summaries. Unlike metrics that account for semantic or syntactic accuracy, ROUGE focuses purely on surface-level lexical matches (Citarella et al., 2025). Specifically, ROUGE-L calculates an F-measure that balances precision (*P*) and recall (*R*) using the *β* parameter, as outlined in equation (5). This enables an objective assessment of summary quality based on the longest common subsequence between texts.

$$\begin{aligned} ROUGE-L=\frac{\left( 1+\beta^{2} \right).P.R}{R+\beta^{2}.P}\#\left( 5 \right) \end{aligned}$$

Inference time refers to the duration required for a deep learning model to generate an output in response to a given input. This metric is important for real-time applications, as it directly impacts the system’s responsiveness to user interactions. Formally, inference time (*T)* is defined as the difference between the start time ($t_{start}$) and the end time ($t_{end}$) of the processing operation (Ndimbo et al., 2025), as shown in equation (6). Efficient inference is essential for ensuring seamless user experiences, especially in latency-sensitive environments.

$$\begin{aligned} T=t_{end}-t_{start}\#\left( 6 \right) \end{aligned}$$

BERTScore is a semantic evaluation metric that differs from traditional surface-level methods by leveraging the contextual representation power of the BERT model to assess the similarity between generated and reference summaries. Rather than focusing solely on exact word matches, BERTScore captures meaning by computing the cosine similarity between contextual embeddings of words from candidate and reference texts. This enables accurate evaluation of paraphrased outputs that preserve semantic integrity. Given a candidate sentence $x=\left\{ x_{1},x_{2},x_{3},\ldots,x_{n} \right\}$, and a reference sentence $y=\left\{ y_{1},y_{2},y_{3},\ldots,y_{n} \right\}$, the method first extracts embeddings $E\left( x_{i} \right),E\left( y_{j} \right)$, then calculates precision and recall through maximal cosine similarity scores, culminating in an *F_1_* score that reflects overall alignment, as detailed in Equation (7).

$$\begin{aligned} Precision=\frac{1}{\left| x \right|}\sum_{x_{i}\epsilon x} \max_{y_{i}\epsilon y} \cos\left( E\left( x_{i} \right), E\left( y_{j} \right) \right)\#\left( 7 \right) \end{aligned}$$

$$Recall= \frac{1}{\left| y \right|}\sum_{y_{i}\epsilon y} \max_{x_{i}\epsilon x} \cos\left( E\left( y_{i} \right), E\left( x_{j} \right) \right)$$

$$F_{1}=\frac{2.Precision.Recall}{Precision+Recall}$$

BLEURT is an evaluation metric designed to assess both the fluency and semantic adequacy of a candidate sentence by comparing it to a reference sentence. It is particularly effective in tasks such as machine translation, summarization, and text generation, where output quality is crucial. Unlike traditional rule-based metrics, BLEURT is trained as a regression model to produce quality scores that align closely with human judgments (Nofal et al., 2025). This learning-based approach allows it to capture nuanced linguistic features and contextual relevance more effectively.

The Dialogue RPT score evaluates the quality of dialogue responses using a pre-trained model fine-tuned on ranking-based data. Its primary goal is to predict which response among multiple candidates is superior in quality. Rather than producing an absolute evaluation, the model assigns a relative score that reflects how a given response compares to others in the set. A higher score indicates a stronger preference, signifying that the model considers the response more appropriate or relevant **(Gao et al., 2020).**

**REFERENCES**

Citarella, A. A., Barbella, M., Ciobanu, M. G., De Marco, F., Di Biasi, L., & Tortora, G. (2025). Assessing the effectiveness of ROUGE as unbiased metric in Extractive vs. Abstractive summarization techniques. *Journal of Computational Science*, *87*, 102571. <https://doi.org/10.1016/j.jocs.2025.102571>

Denkowski, M., & Lavie, A. (2014, June, 2014). Meteor universal: Language specific translation evaluation for any target language. Proceedings of the ninth workshop on statistical machine translation, Maryland, USA, <https://doi.org/10.3115/v1/W14-3348>

Figueroa, R. (2023). *Evaluating adaptive layer freezing through hyperparameter optimization for enhanced fine-tuning performance of language models* Massachusetts Institute of Technology]. Massachusetts Institute of Technology. <https://hdl.handle.net/1721.1/157169>

Gao, X., Zhang, Y., Galley, M., Brockett, C., & Dolan, B. (2020). Dialogue response ranking training with large-scale human feedback data. Proceedings of the 2020 Conference on Empirical Methods in Natural Language Processing, <https://doi.org/10.18653/v1/2020.emnlp-main.28>

Jelinek, F., Mercer, R. L., Bahl, L. R., & Baker, J. K. (1977). Perplexity—a measure of the difficulty of speech recognition tasks. *The Journal of the Acoustical Society of America*, *62*(S1), S63-S63. <https://doi.org/10.1121/1.2016299>

Jin, K., Xiong, Y., Lou, S., & Yu, Z. (2024). MAFD: Multiple adversarial features detector for enhanced detection of text based adversarial examples. *Neural Processing Letters*, *56*(6), 1-24. <https://doi.org/10.1007/s11063-024-11710-0>

Nofal, A. B., Ali, H., Hadi, M., Ahmad, A., Qayyum, A., Johri, A., Al-Fuqaha, A., & Qadir, J. (2025). AI-enhanced interview simulation in the metaverse: Transforming professional skills training through VR and generative conversational AI. *Computers and Education: Artificial Intelligence*, *8*, 100347. <https://doi.org/10.1016/j.caeai.2024.100347>

Rivera-Trigueros, I. (2022). Machine translation systems and quality assessment: A systematic review. *Language Resources and Evaluation*, *56*(2), 593-619. <https://doi.org/10.1007/s10579-021-09537-5>
