# Supplementary material for: Designing metaverse interaction systems for the Turkish language enhanced by fine-tuning and retrieval-augmented generation (RAG)
Source: Sci Rep. 2026 Apr 20;16:18294. doi: 10.1038/s41598-026-35392-x (PMC13261109; doi:10.1038/s41598-026-35392-x)
Supplement: Supplementary file 4 — Supplementary Information 4. [file 41598_2026_35392_MOESM4_ESM.docx]

Appendix D. Parameter Updates

**Table 1.** Parameter Updates for duxx/DeepSeek-R1-Distill-Qwen-1.5B-Turkish

| **Category** | **Parameter** | **Initial Setting** | **Updated Setting** | **Description** |
| --- | --- | --- | --- | --- |
| Model Loading | model_name | - | DeepSeek-R1-Distill-Qwen-1.5B-Turkish | Larger and more recent decoder-only architecture with improved reasoning capacity |
|  | trust_remote_code | False | True | Required for Qwen-based custom model implementations |
|  | device_map | – | auto | Efficient device placement for 1.5B parameters |
| Tokenizer | pad_token | None | eos_token | Prevents padding-related runtime errors |
|  | max_length | 1024 | 512 | Balanced memory usage and contextual coverage |
| Precision | fp16 | False | True (GPU) | Accelerates training and reduces memory footprint |
| Batching | per_device_train_batch_size | 4 | 1 | Memory-safe configuration for 1.5B model |
|  | gradient_accumulation_steps | 1 | 4 | Preserves effective batch size (4) |
| Optimization | learning_rate | 5,00E-05 | 2,00E-05 | More stable convergence for large decoder-only models |
|  | num_train_epochs | 1 | 3 | Improved convergence without overfitting |
| Training Strategy | prediction_loss_only | False | True | Reduced overhead, focused loss tracking |
| RAG – Retriever | Embedding model | – | distiluse-base-multilingual-cased-v2 | Lightweight multilingual semantic retrieval |
|  | FAISS index | IndexFlatL2 | IndexFlatIP + normalization | Inner product used as cosine similarity |
|  | top_k | 1 | 2 | Richer contextual grounding |
| RAG – Prompting | Prompt format | Plain concatenation | Instruction-style (Question, Context, Answer) | Improves alignment with instruction tuning |
| Loss Design | Label assignment | labels = input_ids | Prompt masked (-100) | Loss applied only to answer tokens |
| Parameter-Efficient FT | LoRA usage | None | Enabled | Reduces trainable parameters |
|  | LoRA r | – | 8 | Trade-off between capacity and stability |
|  | LoRA alpha | – | 32 | Scaling for low-rank updates |
|  | LoRA target modules | Limited | Expanded (q,k,v,o,gate,up,down) | Better adaptation across attention and MLP layers |
| Quantization (optional) | load_in_8bit | False | True (if available) | Memory-efficient training on supported systems |

In Table 1. ***duxx/DeepSeek-R1-Distill-Qwen-1.5B-Turkish*** model, several parameters were systematically adjusted to ensure training stability, memory efficiency, and alignment with instruction-based generation objectives. Due to the increased model size (1.5B parameters), the per-device batch size was reduced to one, while gradient accumulation was introduced to preserve an effective batch size of four. The learning rate was decreased to $2\times{10}^{-5}$to mitigate instability commonly observed in large decoder-only models.

For RAG-SFT, dense retrieval was implemented using a multilingual sentence embedding model and a cosine-similarity-based FAISS index. A critical modification involved masking prompt tokens during loss computation, ensuring that optimization focused solely on answer generation rather than prompt memorization. Additionally, parameter-efficient fine-tuning was achieved via LoRA, significantly reducing the number of trainable parameters while maintaining adaptation capacity.

**Table 2.** Parameter Updates for redrussianarmy/gpt2-turkish-cased

| **Category** | **Parameter** | **Initial Setting** | **Updated Setting** | **Description** |
| --- | --- | --- | --- | --- |
| Model | Architecture | - | GPT-2 (decoder-only) | Baseline Turkish causal language model |
| Tokenizer | pad_token | Not defined | eos_token | Prevents padding-related runtime errors |
|  | Vocabulary resize | Not applied | resize_token_embeddings | Synchronizes model with tokenizer |
| Input Format | Prompt structure | Simple concatenation | Question,Contexti, Answer order | Encourages context-aware generation |
| Sequence Length | max_length | 1024 (implicit) | 512 | Memory–context balance |
| Batching | per_device_train_  batch_size | 4 | 4 (→ 2/1 if OOM) | Adjusted for GPU memory constraints |
|  | gradient_  accumulation_steps | 1 | 2 (optional 4) | Preserves effective batch size |
| Optimization | learning_rate | Default | 5,00E-05 | Stable convergence for 124M GPT-2 |
|  | num_train_epochs | 1 | 3 | Improved learning stability |
| Precision | fp16 | False | True (CUDA GPU) | Faster training, lower memory usage |
| Loss Design (RAG-SFT) | Label assignment | labels = input_ids | Prompt masked (-100) | Loss applied only to answer tokens |
| RAG – Retriever | Embedding model | – | distiluse-base-multilingual-cased-v2 | Lightweight semantic retrieval |
|  | FAISS index | IndexFlatL2 | IndexFlatIP + normalization | Cosine similarity approximation |
|  | top_k | 1 | 2 | Richer contextual grounding |
| Training Control | prediction_loss_only | False | True | Reduced logging overhead |
|  | Checkpointing | Frequent | Epoch / limited saves | Disk efficiency |
| Deployment | Push to Hub | Manual | push_to_hub=True | Reproducible model sharing |

In Table 2. the redrussianarmy/gpt2-turkish-cased model, several parameters were systematically adjusted to improve training efficiency and stability under limited computational resources. In particular, gradient accumulation and mixed-precision training were employed to achieve a larger effective batch size without exceeding GPU memory constraints. Logging and checkpointing frequencies were substantially reduced to minimize I/O overhead, resulting in faster training iterations. Evaluation-related settings were disabled during exploratory experiments to further streamline the process.

**Table 3**. Parameter Updates for *LLaMA-2-7B*

| **Category** | **Parameter** | **Initial Setting** | **Updated Setting** | **Description** |
| --- | --- | --- | --- | --- |
| Model Loading | load_in_4bit | Disabled | Enabled (NF4) | Reduced GPU memory usage and enabled training of the 7B model under limited VRAM conditions. |
| Quantization | bnb_4bit_  quant_type | – | nf4 | Provided better numerical stability compared to FP16-only setups. |
| Precision | bnb_4bit_  compute_dtype | fp16 | bf16 (Ampere+) | Improved numerical stability and throughput on compatible GPUs. |
| Batching | per_device_  train_batch_size | 2 | 1 | Prevented out-of-memory errors on Colab GPUs. |
| Batching | gradient_  accumulation_steps | 16 | 8 (or 4) | Reduced training time while preserving convergence behavior. |
| Batching | Effective Batch Size | 16 | 8.Nis | Faster iteration cycles with comparable loss convergence. |
| Optimization | learning_rate | 5,00E-05 | 2,00E-04 | More suitable for LoRA-based adaptation. |
| Training Control | num_train_epochs | 1 | 1 (unchanged) | Epoch-based training was kept minimal due to large dataset size. |
| Training Control | max_steps | Not set | 500–1000 | Allowed explicit control over total training duration. |
| Warmup | warmup_ratio | 0.10 | 0.03 | Prevented over-warmup in short training runs. |
| Checkpointing | save_steps | 500 | 2000–5000 | Reduced I/O overhead during training. |
| Logging | logging_steps | 50 | 100–200 | Decreased logging overhead. |
| Data Loading | dataloader_  num_workers | 2 | 0 | Avoided multiprocessing overhead in Colab. |
| Memory Optimization | gradient_  checkpointing | Enabled | Disabled | Improved training speed under 4-bit + LoRA setup. |
| Sequence Length | MAX_LEN | 768 | 512 | Significantly reduced computation time per step. |
| Optimizer | optim | adamw | paged_adamw_8bit | Lower memory footprint and improved efficiency. |
| LoRA Configuration | target_modules | Default | q, k, v, o, gate, up, down_proj | Ensured full coverage of LLaMA attention and MLP layers. |

In Table 3. the *LLaMA-2-7B* model under limited computational resources, a 4-bit quantization strategy (NF4) combined with LoRA-based fine-tuning was adopted. This configuration significantly reduced GPU memory usage while preserving numerical stability, particularly when using bfloat16 precision on compatible hardware. Due to memory constraints, the per-device batch size was set to one, and the effective batch size was controlled through gradient accumulation, which was progressively reduced to accelerate training without degrading convergence behavior. The learning rate was increased to better suit LoRA adaptation, while the warmup ratio was lowered to avoid excessive warmup in short training schedules. To further optimize runtime performance, input sequence length was shortened, checkpoint saving and logging frequencies were reduced, and data loading was simplified to minimize overhead in the python programing. Additionally, an 8-bit AdamW optimizer and carefully selected LoRA target modules covering both attention and feed-forward layers were employed to ensure efficient parameter updates.

**Table 4.** MT5 Encoder–Decoder Models (mt5-base, mt5-small)

| **Category** | **Parameter** | **Initial Setting** | **Updated Setting** | **Description** |
| --- | --- | --- | --- | --- |
| Model Scale | Model variant | mt5-base | mt5-small (final) | mt5-base showed numerical instability (fp16 overflow, nan loss) under long RAG contexts. mt5-small provided more stable gradients, lower memory usage, and smoother convergence. |
| Precision | fp16 / bf16 | Enabled | Disabled | Mixed precision caused gradient overflow and inf/nan loss values, especially with long encoder inputs. Disabling improved numerical stability. |
| Learning Rate | learning_rate | 2,00E-05 | 1,00E-05 | High learning rate caused unstable loss with RAG-enriched inputs. Reduced LR stabilized training dynamics. |
| Loss Computation | Label masking | Incomplete / implicit | Explicit pad → -100 masking | Ensured that loss is computed only on learnable tokens; resolved loss=0.000 issue. |
| Dataset Filtering | Invalid label samples | Included | Removed | Samples with fully masked labels caused loss collapse; filtering restored valid loss computation. |
| Input Length | MAX_SOURCE_LEN | Unbounded / default | 384 | Long RAG contexts increased memory usage and instability. Limiting encoder input improved efficiency and stability. |
| Target Length | MAX_TARGET_LEN | Unbounded / default | 96 | Prevented excessive decoder sequence length and unnecessary padding. |
| Gradient Control | max_grad_norm | Not set | 1.0 | Gradient clipping prevented exploding gradients and nan loss values. |
| Batch Size | per_device_train_batch_size | 1 | 1 (unchanged) | GPU memory constraints required keeping batch size minimal. |
| Effective Batch | gradient_accumulation_steps | 1 | 8–16 | Enabled larger effective batch size without increasing GPU memory usage; improved gradient smoothness. |
| Logging | logging_steps | Very frequent | Reduced (50–200) | Excessive logging slowed training; reduced frequency improved runtime efficiency. |
| Checkpointing | save_steps | Very frequent | Reduced (500–1000) | Prevented I/O bottlenecks and disk overuse during training. |

In Table 4, the MT5 encoder–decoder model, several parameters were iteratively adjusted to address loss computation failures and numerical instability. In particular, explicit label masking, dataset filtering, reduced learning rates, gradient clipping, and constrained input lengths were applied. Due to persistent instability observed in the mt5-base model under RAG-enriched contexts, the mt5-small variant was ultimately preferred, resulting in a stable and reproducible training process.

**Table 5.** MT5 Encoder–Decoder Models (Fine-Tuning + RAG)

| **Category** | **Parameter** | **Initial Setting** | **Updated Setting** | **Rationale** |
| --- | --- | --- | --- | --- |
| Model Architecture | Architecture | - | Encoder–Decoder (facebook/mbart-large-50-many-to-many-mmt) | The core architecture was retained; observed issues were configuration- rather than architecture-related. |
| Language Configuration | src_lang /  tgt_lang | Not specified | tr_TR / tr_TR | Explicit language codes are mandatory in mBART-50; missing codes caused tokenization and decoder start errors. |
| Language Configuration | decoder_  start_token_id | Default / None | lang_code_to_id  ["tr_TR"] | Ensured correct decoder initialization with the target language token. |
| Input Length | MAX_SOURCE  _LEN | 512 (default) | 384–512 (controlled) | RAG-augmented contexts led to overly long encoder inputs, increasing memory usage and instability. |
| Target Length | MAX_TARGET  _LEN | 128 | 96–128 (controlled) | Reduced the risk of producing fully padded target sequences, which caused zero-loss issues. |
| Batch Size | per_device_  train_batch_size | 2–4 | 1 | mBART-large is memory-intensive; smaller batch size prevented OOM and instability. |
| Effective Batch Size | Effective batch size | 2–4 | 16 | Achieved via gradient accumulation to maintain stable gradient updates. |
| Optimization | gradient_  accumulation_steps | 1 | 16 | Compensated for small batch size and improved gradient stability. |
| Precision | fp16 | Enabled | Disabled | Mixed-precision training caused numerical overflow (inf/nan) with long sequences. |
| Precision | bf16 | Disabled | Disabled | Kept disabled for hardware compatibility and numerical stability. |
| Learning Rate | learning_rate | 3,00E-05 | 2e-5 → 1e-5 | Higher learning rates led to unstable training in seq2seq + RAG settings. |
| Warmup Strategy | warmup_ratio | 0.0 | 0.03–0.06 | Improved early-stage optimization stability for encoder–decoder training. |

In Table 5. during the fine-tuning of the mBART (facebook/mbart-large-50-many-to-many-mmt) model, several parameter adjustments were required to ensure numerical stability and correct loss computation. In particular, explicit language code configuration (src_lang, tgt_lang, and decoder_start_token_id) was necessary due to the multilingual nature of mBART-50. Training instability issues such as zero loss and NaN/Inf values were addressed by correcting label masking (pad tokens mapped to −100), reducing the learning rate, disabling mixed-precision training, and applying gradient clipping. Additionally, batch size was minimized and compensated through gradient accumulation to achieve a stable effective batch size. For RAG-based experiments, encoder input length and the number of retrieved contexts were constrained to prevent excessive sequence lengths. These adjustments collectively enabled a stable and reproducible training process for the mBART model.
