# Supplementary material for: Designing metaverse interaction systems for the Turkish language enhanced by fine-tuning and retrieval-augmented generation (RAG)
Source: Sci Rep. 2026 Apr 20;16:18294. doi: 10.1038/s41598-026-35392-x (PMC13261109; doi:10.1038/s41598-026-35392-x)
Supplement: Supplementary file 5 — Supplementary Information 5. [file 41598_2026_35392_MOESM5_ESM.docx]

| gpt2_fine_tuning_responses | | | | |
| --- | --- | --- | --- | --- |
| **ID** | **Questions**  **(Turkish Language)** | **Answer**  **(Turkish Language)** | **Question (Translation)** | **Answer (Translation)** |
| 1 | Kahvaltı hazır mı? | Evet, hafta sonu için hazırlanıyorum. Bu harika! Ne zamandır içiyorsun?  Duygusal deneyim lere sahip olmanın tek | Is breakfast ready? | Yes, I am preparing for the weekend. That’s great! How long have you been drinking? The only way of having emotional experience s |
| 2 | Bugün dışarı çıkacak mıyız? | Hayır. Neden olmasın ki?   Duygusal deneyim lerle iligli diyalog larda ınırdım. Onu özlüyorum | Are we going out today? | No. Why wouldn’t it be? I would be limited in dialogues related to emotional experience s. I miss him. |
| 3 | Film izleyelim mi? | Evet, en iyi filmlerinden bazıları.  Özlem lerle iligli diyalog ını seviyorum  Duygusal deneyim | Shall we watch a movie? | Yes, some of his best movies. I like the dialogue related to longing s Emotional experience |
| 4 | Hava bugün soğuk mu? | Evet, bu gece soğuk ve güneşli olacak.  Hava durumuyla iligli diyalog ını incele iyorsun. Yakında daha | Is it cold today? | Yes, it will be cold and sunny tonight. You are analyz ing the dialogue related to the weather. Soon more |
| 5 | Ders başladı mı? | Evet ve bir süre önce okulumuzu terk etti.  Okulda geçen konuşmalar ını izleyerek zaman geçirin  Duygusal deneyim | Has the class started? | Yes and he left our school some time ago. Spend time by watching the conversations that take place at school Emotional experience |
| 6 | Eve geç mi kalacağız? | Evet. Çok mutluyum!  Mutluluk ını geri getirme : (25 yıl)  Duygusal | Will we be late getting home? | Yes. I am very happy! Bringing back your happiness: (25 years) Emotional |
| 7 | Toplantı iptal mi? | Evet, onay aldım. Bu hiç eğlenceli değil. Çok fazla testten geçtim ama sonunda çok kötü olduğunu düşündüm.  Duygusal deneyim | Is the meeting cancelled? | Yes, I got approval. This is not fun at all. I went through many tests but in the end I thought it was very bad. Emotional experience |
| 8 | Çay ister misin? | Evet, sanırım bu doğru.  Günlük konuşma diyalog ını latan şeyler ektedir. Kendi başına | Would you like some tea? | Yes, I think this is correct. The things that start the daily conversation dialogue are attached. On its own |
| 9 | İnternet çalışıyor mu? | Evet ama telefon hatlarım şu anda çok zayıf. Bu konuda kendini daha iyi hissetmelisin! Telefonla konuşurken iyi şanslar ve sonuçlar için | Is the internet working? | Yes but my phone lines are very weak right now. You should feel better about this! For good luck and results while talking on the phone |
| 10 | Otobüs geldi mi? | Evet, ben de yaptım. Her şey yolunda gitti  Duygusal deneyim ve sosyal ilişki lerini yeniden canlandır | Has the bus arrived? | Yes, I did too. Everything went well Revive emotional experience and your social relationship s |
| 11 | Saat kaç oldu? | Ah, bunu duyduğuma üzüldüm. Bir hafta önce evimi temizlemişti ve evde yalnızdım.  Duygusal deneyim land ımı dile getirme konusunda | What time is it? | Ah, I am sorry to hear that. He had cleaned my house a week ago and I was alone at home. About expressing my emotional experience ed |
| 12 | Işıkları kapattın mı? | Evet! Umarım her şey yolunda gider.  Ev ortamında geçen özel diyalog lardan birinde geçen diyalog larda geçen diyalog | Did you turn off the lights? | Yes! I hope everything goes well. The dialogue that takes place in one of the special dialogues occurring in the home environment |
| 13 | Kitabı bulabildin mi? | Evet! Bir sürü kitap okudum.  Günlük konuşma diyalog ını ektin eyen harika bir oyundu  Duygusal deneyim | Were you able to find the book? | Yes! I read a lot of books. It was a great game that did not add to the daily conversation dialogue Emotional experience |
| 14 | Müzik açalım mı? | Çok müzikli olmalı.  Günlük konuşma diyalog ını ve şarkı söylemeyi dene  Duygusal deneyim | Shall we play some music? | It must be very musical. Try daily conversation dialogue and singing Emotional experience |
| 15 | Sınav zor muydu? | Evet. Sanırım çok iyi geçti ama bu yıl tekrar başlamak için elimden geleni yapıyorum.  Duygusal deneyim lere șerinin tadını çıkarın | Was the exam difficult? | Yes. I think it went very well but I am doing my best to start again this year. Enjoy the flavor of emotional experience s |
| 16 | Telefonum nerede? | Evim, eşim ve ben yaklaşık 4 ay önce, eski evimden yeni taşındık.  Duygusal deneyim le iligli | Where is my phone? | My home, my spouse and I moved from my old house to a new one about 4 months ago. Related to emotional experience |
| 17 | Bugün yağmur var mı? | Hayır, sadece çok sıcak.  Ev ortamında geçen özel diyalog lara göre ilişki kurabiliyorum.  Duygusal deneyim lerle ilgili | Is it going to rain today? | No, it is just very hot. I can establish a relationship according to the special dialogues taking place in the home environment. Related to emotional experience s |
| 18 | Kapıyı kilitledin mi? | Evet! Bekleyemem. Bu biraz uzun sürdü ama artık penceremin her yerinde oturuyordum ve kapı açıktı.  Duygusal deneyim land | Did you lock the door? | Yes! I can’t wait. This took a bit long but I was sitting all over my window and the door was open. Emotional experience ed |
| 19 | Proje bitti mi? | Ne kadar erken başladınız?  Duygusal deneyim lem le iligli diyalog lerle ilgili diyalog | Is the project finished? | How early did you start? Dialogue related to dialogues about my emotional experience lem |
| 20 | Kargo geldi mi? | Evet, teslimatçıya bildireceğim. Ah hayır! Çok üzgün olmalısın!! O zaman sana nasıl davranacağını söyle! | Has the package arrived? | Yes, I will inform the delivery person. Oh no! You must be very sorry!! Then tell how he should treat you! |

| gpt2_RAG_responses | | | | |
| --- | --- | --- | --- | --- |
| **ID** | **Questions**  **(Turkish Language)** | **Answer**  **(Turkish Language)** | **Question (Translation)** | **Answer (Translation)** |
| 1 | Kahvaltı hazır mı? | Yemek ve yiyeceklerle ilgili diyaloglar - Yemek ve yiyeceklerle ilgili diyaloglar - Yemek ve yiyeceklerle ilgili diyaloglar - Yemek ve | Is breakfast ready? | Dialogues related to food and foods - Dialogues related to food and foods - Dialogues related to food and foods - Food and |
| 2 | Bugün dışarı çıkacak mıyız? | Sabah 5'te kalkacağım. Umarım daha iyi olur ve çok sinir bozucudur. - Üzüntü - Üzüntü - Üzüntü | Are we going out today? | I will wake up at 5 in the morning. I hope it gets better and it is very annoying. - Sadness - Sadness - Sadness |
| 3 | Film izleyelim mi? | Evet, değil. En azından onun için çok eğlenceli bir film izleyeceğim. - Günlük konuşma diyalog - Günlük konuşma diyalog - Günlük konuşma | Shall we watch a movie? | Yes, not. At least I will watch a very fun movie for him. - Daily conversation dialogue - Daily conversation dialogue - Daily conversation |
| 4 | Hava bugün soğuk mu? | Hava nasıldı? Bu konuda endişeliyim. - Hava durumuyla iligli diyalog - Hava durumuyla iligli diyalog - Hava durumuyla | Is it cold today? | How was the weather? I am worried about this. - Dialogue related to the weather - Dialogue related to the weather - With the weather |
| 5 | Ders başladı mı? | Evet, sadece bir ders ama sonunda mezun oldum. - Okulda geçen konuşmalar - Okulda geçen konuşmalar - Okulda geçen konuşmalar - Okulda geçen | Has the class started? | Yes, just one class but in the end I graduated. - Conversations taking place at school - Conversations taking place at school - Conversations taking place at school - Taking place at school |
| 6 | Eve geç mi kalacağız? | Evet, her şey yoluna girecek. İyi olacak ama bu konuda gerçekten gerginim. - Korku / Kaygı - Korku / Kaygı | Will we be late getting home? | Yes, everything will be fine. It will be good but I am really nervous about this. - Fear / Anxiety - Fear / Anxiety |
| 7 | Toplantı iptal mi? | Hayır, ama yine de bu konuda kendimi biraz kötü hissettim. - Üzüntü - Üzüntü - Üzüntü - Üzüntü | Is the meeting cancelled? | No, but still I felt a little bad about this. - Sadness - Sadness - Sadness - Sadness |
| 8 | Çay ister misin? | Hayır, sadece bazı arkadaşlarımla bira içiyorum. Bu konuda hiçbir şey bilmiyorum. - Yemek ve yiyeceklerle ilgili diyaloglar - Yemek ve yiyecek | Would you like some tea? | No, I am just drinking beer with some of my friends. I know nothing about this. - Dialogues related to food and foods - Food and foods |
| 9 | İnternet çalışıyor mu? | Bilgisayarda telefon kullanıyorum. - İş bulmayla ilgili diyalog - İş bulmayla ilgili diyalog - İş bulmayla ilgili diyalog - İş | Is the internet working? | I am using a phone on the computer. - Dialogue related to finding a job - Dialogue related to finding a job - Dialogue related to finding a job - Job |
| 10 | Otobüs geldi mi? | Toplu taşıma araçlarında. Bütün bunlara sahip olduğum için çok mutluyum ve iyiyim! - Mutluluk - Mutluluk - Mutluluk - Mutluluk < | Has the bus arrived? | On public transportation vehicles. I am very happy and fine because I have all of these! - Happiness - Happiness - Happiness - Happiness < |
| 11 | Saat kaç oldu? | 14.00'te, yani 5 dakika içinde, bu yıl daha iyi olacak! - Günlük konuşma diyalog - Günlük konuşma diyalog - | What time is it? | At 14.00, that is in 5 minutes, it will be better this year! - Daily conversation dialogue - Daily conversation dialogue - |
| 12 | Işıkları kapattın mı? | Hayır, ama şimdi bir sürü yeni oyun var. - Özlem - Özlem - Özlem - Özlem <cevap> Sadece tek bir | Did you turn off the lights? | No, but now there are lots of new games. - Longing - Longing - Longing - Longing Only a single |
| 13 | Kitabı bulabildin mi? | Evet, sadece iki kez okudum. Sanırım bunu başardığım için çok mutluydum. - Mutluluk - Mutluluk - Mutluluk - Mutluluk | Were you able to find the book? | Yes, I only read it twice. I think I was very happy because I achieved this. - Happiness - Happiness - Happiness - Happiness |
| 14 | Müzik açalım mı? | Evet, evet! Müzik albümüne bayıldım. Bu arada o da müzik çalıyordu. - Özlem - Özlem - Özlem - Özlem | Shall we play some music? | Yes, yes! I loved the music album. By the way, he was also playing music. - Longing - Longing - Longing - Longing |
| 15 | Sınav zor muydu? | Hayır, çok fazla sınav ve çok az çalışma. Bu yüzden iyi yaptım. - Okulda geçen konuşmalar - Okulda geçen konuşmalar - Okulda geçen | Was the exam difficult? | No, too many exams and very little studying. That is why I did well. - Conversations taking place at school - Conversations taking place at school - Taking place at school |
| 16 | Telefonum nerede? | (1 saat içinde) Sadece telefonumu bekliyorum. Bana da mesaj gönderecekler! - Günlük konuşma diyalog - Günlük konuşma diyalog - Günlük | Where is my phone? | (Within 1 hour) I am just waiting for my phone. They will also send me a message! - Daily conversation dialogue - Daily conversation dialogue - Daily |
| 17 | Bugün yağmur var mı? | Hayır, bir süredir yağmur yağmıyor. Çok korkmuştum. Bir daha asla güneş ışığına çıkmadım. - Hava durumuyla iligli diyalog | Is it going to rain today? | No, it has not rained for a while. I was very scared. I never went out into sunlight again. - Dialogue related to the weather |
| 18 | Kapıyı kilitledin mi? | Hayır ama kimse bana söylemedi. Biraz utandım. Sadece çok üzüldüm. - Üzüntü - Üzüntü - Üzüntü - | Did you lock the door? | No but nobody told me. I was a little embarrassed. I was just very sad. - Sadness - Sadness - Sadness - |
| 19 | Proje bitti mi? | Hayır, bir dahaki sefere daha iyi olacağını sanmıyorum. Sadece biraz uzun sürdü ama bu hafta sonu çok iyiydi. - Parkta ve seyahat sırasında geçen | Is the project finished? | No, I do not think it will be better next time. It just took a little long but this weekend was very good. - Taking place in the park and during travel |
| 20 | Kargo geldi mi? | Hayır. O zamanlar çok utandım! Çok hastaydım ama ben de aynı şekilde hissettim - Üzüntü - Üzüntü - Üzüntü | Has the package arrived? | No. I was very embarrassed at that time! I was very sick but I felt the same way too - Sadness - Sadness - Sadness |

| Llama_fine_tuning_responses | | | | |
| --- | --- | --- | --- | --- |
| **ID** | **Questions**  **(Turkish Language)** | **Answer**  **(Turkish Language)** | **Question (Translation)** | **Answer (Translation)** |
| 1 | Kahvaltı hazır mı? | çünkü kahvalt hazr. | Is breakfast ready? | because breakfas t is read y. |
| 2 | Bugün dışarı çıkacak mıyız? | çünkü doada bizim gibi yaşamay salar. | Are we going out today? | because in natur e they do not liv e like us. |
| 3 | Film izleyelim mi? | çünkü onlar da bu dünyada yaşadmz salar. | Shall we watch a movie? | because they also in this world liv ed s. |
| 4 | Hava bugün soğuk mu? | çünkü souk yaşadmz salar. | Is it cold today? | because col d liv ed s. |
| 5 | Ders başladı mı? | çünkü doada bulunmadmz salar. | Has the class started? | because in natur e we did not exis t s. |
| 6 | Eve geç mi kalacağız? | çünkü ev yaşamay salar. | Will we be late getting home? | because they do not liv e at hom e. |
| 7 | Toplantı iptal mi? | çünkü toplant iptal ediyor. | Is the meeting cancelled? | because the meetin g cancel s. |
| 8 | Çay ister misin? | ay ay misin? | Would you like some tea? | are you ay ay? |
| 9 | İnternet çalışıyor mu? | internet çalşyor. | Is the internet working? | internet is workin g. |
| 10 | Otobüs geldi mi? | çünkü otobüs geldi. | Has the bus arrived? | because the bus arrive d. |
| 11 | Saat kaç oldu? | çünkü saat yaşadmz snrl dikkatli kullanmalyz. | What time is it? | because the time we liv ed limi t we must use carefull y. |
| 12 | Işıkları kapattın mı? | çünkü onlar da bu dünyada bizim gibi yaşamay salar. | Did you turn off the lights? | because they also in this world do not liv e like us. |
| 13 | Kitabı bulabildin mi? | çünkü onlar da bu dünyada bulabilirsiniz. | Were you able to find the book? | because you can also find them in this world. |
| 14 | Müzik açalım mı? | çünkü müzik açlyorum. | Shall we play some music? | because I am openin g music. |
| 15 | Sınav zor muydu? | Snav zor muydu? | Was the exam difficult? | Was the exa m difficul t? |
| 16 | Telefonum nerede? | - | Where is my phone? | - |
| 17 | Bugün yağmur var mı? | çünkü yaşamay var. | Is it going to rain today? | because there is livin g. |
| 18 | Kapıyı kilitledin mi? | çünkü kapy kilitledi. | Did you lock the door? | because he/she locke d the doo r. |
| 19 | Proje bitti mi? | CEVAP: Proje bitti mi? | Is the project finished? | ANSWER: Is the project finished? |
| 20 | Kargo geldi mi? | çünkü onlar geldi. | Has the package arrived? | because they arrive d. |

| Llama_rag_responses | | | | |
| --- | --- | --- | --- | --- |
| **ID** | **Questions**  **(Turkish Language)** | **Answer**  **(Turkish Language)** | **Question (Translation)** | **Answer (Translation)** |
| 1 | Kahvaltı hazır mı? | Hayır, bu yüzden ona baktığımı düşündüğümde kendimi kötü hissettim. Bu çok komik! Ben de aynısını yapardım. Umarım bir daha asla. | Is breakfast ready? | No, so when I thought that I was looking at him/her I felt bad. This is very funny! I would do the same. I hope never again. |
| 2 | Bugün dışarı çıkacak mıyız? | Evet, biraz daha sıcak olabilir. Ah benim! Umarım yavaş gider ve bir dahaki sefere daha iyi gelebilirsiniz! İş arkadaşlarından biri: O kadar yavaş ge | Are we going out today? | Yes, it can be a little warmer. Oh my! I hope it goes slow and you can come better next time! One of the coworkers: So slow go |
| 3 | Film izleyelim mi? | Yok be. Kızlarım dışarı çıktı. | Shall we watch a movie? | No man. My daughters went out. |
| 4 | Hava bugün soğuk mu? | Evet, çok sıcak. Ah be adam! Kızım bu hafta sonu bir küçük yolculuk yapmak istiyor. Umarım güzel vakit geçirirsiniz! | Is it cold today? | Yes, very hot. Oh man! My daughter wants to make a small trip this weekend. I hope you have a nice time! |
| 5 | Ders başladı mı? | Evet, ve her zaman çok hızlı atlıyorlar. Gerçekten bir sürü yaratıcı insan gibi.Bu harika! Tebrikler! Umarım senin için iyi gider. | Has the class started? | Yes, and they always jump very fast. Really like many creative people. This is great! Congratulations! I hope it goes well for you. |
| 6 | Eve geç mi kalacağız? | Sanırım geçti ama henüz onu bulamadık. Korku filmleri hakkında konuşuyorum. | Will we be late getting home? | I think it passed but we have not found it yet. I am talking about horror movies. |
| 7 | Toplantı iptal mi? | Evet, kesinlikle. Birden fazla çeşit toplantı bulunabilir. Herhangi bir yöntemde daha iyi bulduğunuzu düşünmelisiniz. | Is the meeting cancelled? | Yes, definitely. More than one type of meeting can be found. You should think that you find it better in any method. |
| 8 | Çay ister misin? | Evet, birkaç şey yapıyorum ve bu benim ilk oyunum. Ne tür bir iş? Sanırım iyi olacak. Bence bir dünyanın en büyük oy | Would you like some tea? | Yes, I am doing a few things and this is my first game. What kind of job? I think it will be good. I think one of the world’s biggest ga |
| 9 | İnternet çalışıyor mu? | Yapıyor, yaklaşık bir saat sürdü. Birkaç arkadaşımla gittik ve çok eğlenceliydi! Arkadaşlık / Sosyal ilişki, Mutluluk | Is the internet working? | It does, it took about one hour. We went with a few friends and it was very fun! Friendship / Social relationship, Happiness |
| 10 | Otobüs geldi mi? | Evet, otobüs 15 dakika sonra geldi. Korku filmleri, Duygusal Korku filmleri, Korku / Korku / Korku, Sanal gerçeklik filmleri, Yalnı | Has the bus arrived? | Yes, the bus came after 15 minutes. Horror movies, Emotional horror movies, Fear / Fear / Fear, Virtual reality movies, Alon e |
| 11 | Saat kaç oldu? | O 35'teydi. Kız arkadaşımla ve birbirimizle geçirdiğimiz zamanı özlüyorum. Bu çok zor olmalı. Bazen insanlar bunu yapmak istediğini bilmi | What time is it? | It was at 35. I miss the time I spent with my girlfriend and with each other. This must be very hard. Sometimes people know that they want to do this |
| 12 | Işıkları kapattın mı? | hayır, sadece yüksek bir çekiç mi istiyorsun? Bağlam için teşekkür ederim. Çekiçleri de seviyorum, çekiçleri aldım. Hala onları almay | Did you turn off the lights? | no, do you just want a high hammer? Thank you for the context. I also like hammers, I bought the hammers. Still not taking them |
| 13 | Kitabı bulabildin mi? | Evet, onu yaklaşık bir ay önce aldım. O kadar uzun zamandır bekliyordum ki. O kadar uzun süre beklemek zorunda kalmanız çok kötü. | Were you able to find the book? | Yes, I bought it about a month ago. I had been waiting for so long. It is very bad that you have to wait for such a long time. |
| 14 | Müzik açalım mı? | Evet, dürüst olmak gerekirse şimdiye kadar kendi başına oynamamı sağlıyor. Bir şeye ihtiyacın varken bunu yapabileceğini düşünmemiş | Shall we play some music? | Yes, honestly it makes me play on my own until now. I did not think that it could do this when you need something |
| 15 | Sınav zor muydu? | Evet, bunu düşündüğümden emin değilim. Sanırım sadece geliştirmeye çalıştım ve sonunda aldım. İyi şanslar! Bu konuda gergin misin? | Was the exam difficult? | Yes, I am not sure that I thought this. I think I just tried to improve and in the end I got it. Good luck! Are you nervous about this? |
| 16 | Telefonum nerede? | Kız arkadaşım benimle birlikteydi. Oh, bunu duyduğuma üzüldüm. Ben de olurdum. Ona ne dedi? Yemekten çok utandı. Onunla konuşmayı sev | Where is my phone? | My girlfriend was with me. Oh, I am sorry to hear that. I would be too. What did he/she say to her? He/She was very embarrassed about the meal. I like talking with him/her |
| 17 | Bugün yağmur var mı? | Biraz yağmur gibi görünüyor. Çok gürültülü. Oh evet, yağmur muhtemelen olacak. Bu kadar uzun süre geceye kalkmaktan nefret ediyorum. | Is it going to rain today? | It looks a bit like rain. Very noisy. Oh yes, rain will probably happen. I hate getting up to the night for such a long time. |
| 18 | Kapıyı kilitledin mi? | Şu anda yapıyorum.İyi işte. Bunun neden bir soru olduğunu biliyorum. İletişim formumu kullanmayı düşündün mü? | Did you lock the door? | I am doing it right now. It is good. I know why this is a question. Did you think about using my contact form? |
| 19 | Proje bitti mi? | Evet, bu kadar hızlı geçtiğini söyledi. Kendine güvenmiyor olmalısın. Arkadaşlarımın ne yapacağını bilmiyorum. | Is the project finished? | Yes, he/she said that it passed this fast. You must not trust yourself. I do not know what my friends will do. |
| 20 | Kargo geldi mi? | Evet, eve gönderildi. Umarım o kadar çok teslimat etmedi. Evet ama ihtiyacı olanların olmasına rağmen çok fazla yapıyorlar. | Has the package arrived? | Yes, it was sent home. I hope he/she did not make so many deliveries. Yes but even though there are those who need it they do too much. |

| Mbart_finetunned_responses | | | | |
| --- | --- | --- | --- | --- |
| **ID** | **Questions**  **(Turkish Language)** | **Answer**  **(Turkish Language)** | **Question (Translation)** | **Answer (Translation)** |
| 1 | Kahvaltı hazır mı? | Hayır, evet! | Is breakfast ready? | No, yes! |
| 2 | Bugün dışarı çıkacak mıyız? | Hayır, bir gün dışarı çıkacağız. | Are we going out today? | No, we will go out one day. |
| 3 | Film izleyelim mi? | Hayır, filmleri severim. | Shall we watch a movie? | No, I like movies. |
| 4 | Hava bugün soğuk mu? | Hayır, hava çok soğuk. | Is it cold today? | No, the weather is very cold. |
| 5 | Ders başladı mı? | Hayır, değil mi? | Has the class started? | No, isn’t it? |
| 6 | Eve geç mi kalacağız? | Hayır, geç kalacağız. | Will we be late getting home? | No, we will be late. |
| 7 | Toplantı iptal mi? | Hayır, toplantı iptal edildi. | Is the meeting cancelled? | No, the meeting was cancelled. |
| 8 | Çay ister misin? | Hayır, sadece bir tane. | Would you like some tea? | No, just one. |
| 9 | İnternet çalışıyor mu? | Hayır, internet yok. | Is the internet working? | No, there is no internet. |
| 10 | Otobüs geldi mi? | Hayır, değil mi? | Has the bus arrived? | No, isn’t it? |
| 11 | Saat kaç oldu? | Saat 10'daydı. | What time is it? | It was at 10 o’clock. |
| 12 | Işıkları kapattın mı? | Hayır, değilim. | Did you turn off the lights? | No, I am not. |
| 13 | Kitabı bulabildin mi? | Hayır, bu konuda hiçbir fikrim yoktu. | Were you able to find the book? | No, I had no idea about this. |
| 14 | Müzik açalım mı? | Hayır, sadece müzik dinlemeyi seviyorum. | Shall we play some music? | No, I just like listening to music. |
| 15 | Sınav zor muydu? | Evet, çok zordu. | Was the exam difficult? | Yes, it was very difficult. |
| 16 | Telefonum nerede? | Birkaç saatlik mesafede. | Where is my phone? | At a distance of a few hours. |
| 17 | Bugün yağmur var mı? | Hayır, çok yağmur yağıyor. | Is it going to rain today? | No, it is raining a lot. |
| 18 | Kapıyı kilitledin mi? | Hayır kilitledim. | Did you lock the door? | No I locked. |
| 19 | Proje bitti mi? | Hayır, henüz bitmedi. | Is the project finished? | No, it has not finished yet. |
| 20 | Kargo geldi mi? | Hayır, değil mi? | Has the package arrived? | No, isn’t it? |

| Mbart_RAG_responses | | | | |
| --- | --- | --- | --- | --- |
| **ID** | **Questions**  **(Turkish Language)** | **Answer**  **(Turkish Language)** | **Question (Translation)** | **Answer (Translation)** |
| 1 | Kahvaltı hazır mı? | Evet, hazır. | Is breakfast ready? | Yes, ready. |
| 2 | Bugün dışarı çıkacak mıyız? | Hayır, bir gün dışarı çıkacağız. | Are we going out today? | No, we will go out one day. |
| 3 | Film izleyelim mi? | Hayır, film izlemeyi denedim. | Shall we watch a movie? | No, I tried to watch a movie. |
| 4 | Hava bugün soğuk mu? | Evet, hava sıcak. | Is it cold today? | Yes, the weather is hot. |
| 5 | Ders başladı mı? | Hayır, o kadar da kötü değil. | Has the class started? | No, it is not that bad. |
| 6 | Eve geç mi kalacağız? | Hayır, geç kalacağız. | Will we be late getting home? | No, we will be late. |
| 7 | Toplantı iptal mi? | Hayır, toplantı iptal edildi. | Is the meeting cancelled? | No, the meeting was cancelled. |
| 8 | Çay ister misin? | Evet, ben de. | Would you like some tea? | Yes, me too. |
| 9 | İnternet çalışıyor mu? | Evet, internette çalışıyor. | Is the internet working? | Yes, it works on the internet. |
| 10 | Otobüs geldi mi? | Hayır, değil mi? | Has the bus arrived? | No, isn’t it? |
| 11 | Saat kaç oldu? | 4 saat. | What time is it? | 4 hours. |
| 12 | Işıkları kapattın mı? | Hayır, şükürler olsun. | Did you turn off the lights? | No, thank goodness. |
| 13 | Kitabı bulabildin mi? | Hayır, bu konuda hiçbir fikrim yoktu. | Were you able to find the book? | No, I had no idea about this. |
| 14 | Müzik açalım mı? | Hayır, bir tane alacağım. | Shall we play some music? | No, I will buy one. |
| 15 | Sınav zor muydu? | Evet, zordu. | Was the exam difficult? | Yes, it was difficult. |
| 16 | Telefonum nerede? | Telefonun yanında. | Where is my phone? | Next to the phone. |
| 17 | Bugün yağmur var mı? | Evet, yağmur yağıyor. | Is it going to rain today? | Yes, it is raining. |
| 18 | Kapıyı kilitledin mi? | Hayır, neyse ki çok sinirlendim | Did you lock the door? | No, fortunately I got very angry |
| 19 | Proje bitti mi? | Hayır, henüz bitmedi. | Is the project finished? | No, it has not finished yet. |
| 20 | Kargo geldi mi? | Evet, çok fazla. | Has the package arrived? | Yes, very much. |
| Mt5_finetunned_responses | | | | |
| **ID** | **Questions**  **(Turkish Language)** | **Answer**  **(Turkish Language)** | **Question (Translation)** | **Answer (Translation)** |
| 1 | Kahvaltı hazır mı? | Evet, kahvaltı hazır. | Is breakfast ready? | Yes, breakfast is ready. |
| 2 | Bugün dışarı çıkacak mıyız? | Evet, bugün dışarı çıkacaksınız. madde 20) | Are we going out today? | Yes, you will go out today. article 20) |
| 3 | Film izleyelim mi? | Evet, film izleyelim. | Shall we watch a movie? | Yes, let’s watch a movie. |
| 4 | Hava bugün soğuk mu? | Evet, hava bugün çok soğuk olabilir. ilk derece hava durumu tahmincisi olan hava durumu tahmincisidir. | Is it cold today? | Yes, the weather may be very cold today. it is the weather forecaster who is a first degree weather forecaster. |
| 5 | Ders başladı mı? | Evet, dersler başlamıştır. | Has the class started? | Yes, the classes have started. |
| 6 | Eve geç mi kalacağız? | Evet, eve geç kalacağız. | Will we be late getting home? | Yes, we will be late getting home. |
| 7 | Toplantı iptal mi? | Evet, toplantı iptal edilebilir. | Is the meeting cancelled? | Yes, the meeting can be cancelled. |
| 8 | Çay ister misin? | Evet, çay içmek ister misin? | Would you like some tea? | Yes, would you like to drink tea? |
| 9 | İnternet çalışıyor mu? | Evet, internet çalışıyorsa ve sorun çözülmezse, tüketici hakem heyetine başvurarak sorunun çözülmesini talep edebilir, çözüm bulamazsanız tüketici hakem heyetine başvurarak şikayette bulunabilirsiniz. | Is the internet working? | Yes, if the internet is working and the problem is not resolved, you can request the resolution of the problem by applying to the consumer arbitration committee, if you cannot find a solution you can apply to the consumer arbitration committee and file a complaint. |
| 10 | Otobüs geldi mi? | Evet, otobüs geldi. | Has the bus arrived? | Yes, the bus has arrived. |
| 11 | Saat kaç oldu? | Saat kaç oldu? geçici madde 21) | What time is it? | What time is it? provisional article 21) |
| 12 | Işıkları kapattın mı? | Evet, ışıkları kapattınız mı? | Did you turn off the lights? | Yes, did you turn off the lights? |
| 13 | Kitabı bulabildin mi? | Evet, kitabı bulabildin mi? | Were you able to find the book? | Yes, were you able to find the book? |
| 14 | Müzik açalım mı? | Evet, müzik açalım. | Shall we play some music? | Yes, let’s play music. |
| 15 | Sınav zor muydu? | Evet, zordu. | Was the exam difficult? | Yes, it was difficult. |
| 16 | Telefonum nerede? | Telefonum nerede? tck md. 3/f.1) | Where is my phone? | Where is my phone? tpc art. 3/p.1) |
| 17 | Bugün yağmur var mı? | Evet, yağmur bekleniyor. | Is it going to rain today? | Yes, rain is expected. |
| 18 | Kapıyı kilitledin mi? | Evet, kapıyı kilitledin ve kilitledin. | Did you lock the door? | Yes, you locked the door and locked it. |
| 19 | Proje bitti mi? | Evet, proje bittiyse, ilgili idareye veya kamu tüzel kişiliğine ait bir inşaat ruhsatı verilir. bu ruhsat, resmi gazete'de yayımlanarak yürürlüğe girer. | Is the project finished? | Yes, if the project is finished, a construction permit belonging to the relevant administration or public legal entity is issued. this permit enters into force by being published in the official gazette. |
| 20 | Kargo geldi mi? | Evet, kargo geldiyse ve ödeme dekontunuzla birlikte teslim edildiyse, ödeme dekontunuzla birlikte tüketici hakem heyetine başvurabilirsiniz. | Has the package arrived? | Yes, if the cargo has arrived and has been delivered together with your payment receipt, you can apply to the consumer arbitration committee together with your payment receipt. |

| MT5_RAG_responses.csv | | | | |
| --- | --- | --- | --- | --- |
| **ID** | **Questions**  **(Turkish Language)** | **Answer**  **(Turkish Language)** | **Question (Translation)** | **Answer (Translation)** |
| 1 | Kahvaltı hazır mı? | Evet güzel bir kahvaltı hazırladım. | Is breakfast ready? | Yes, I prepared a nice breakfast. |
| 2 | Bugün dışarı çıkacak mıyız? | Evet hava güzel olursa yürüyüşe gidebiliriz. | Are we going out today? | Yes, if the weather is nice we can go for a walk. |
| 3 | Film izleyelim mi? | Evet, bir de yabancı dilde dublaj var. | Shall we watch a movie? | Yes, there is also dubbing in a foreign language. |
| 4 | Hava bugün soğuk mu? | Evet güneşli ama akşama doğru yağmur bekleniyor. | Is it cold today? | Yes, it is sunny but rain is expected towards the evening. |
| 5 | Ders başladı mı? | Evet ilk derste. | Has the class started? | Yes, in the first class. |
| 6 | Eve geç mi kalacağız? | Hayır evde tadilat var. | Will we be late getting home? | No, there is renovation at home. |
| 7 | Toplantı iptal mi? | Evet hava durumu nedeniyle ertelendi. | Is the meeting cancelled? | Yes, it was postponed due to the weather conditions. |
| 8 | Çay ister misin? | Evet bir bardak çay. | Would you like some tea? | Yes, a glass of tea. |
| 9 | İnternet çalışıyor mu? | Evet ama sadece belirli kişiler çalışıyor. | Is the internet working? | Yes, but only certain people are working. |
| 10 | Otobüs geldi mi? | Evet yolcu indiriliyor. | Has the bus arrived? | Yes, the passenger is being dropped off. |
| 11 | Saat kaç oldu? | Saat 10.30da saat 14.00te. | What time is it? | At 10.30 at 14.00. |
| 12 | Işıkları kapattın mı? | Evet gece geç saatlere kadar açık. | Did you turn off the lights? | Yes, it is open until late at night. |
| 13 | Kitabı bulabildin mi? | Hayır evde unutmuşum. | Were you able to find the book? | No, I forgot it at home. |
| 14 | Müzik açalım mı? | Evet fonda bir şeyler çalalım. | Shall we play some music? | Yes, let’s play something in the background. |
| 15 | Sınav zor muydu? | Evet zordu. | Was the exam difficult? | Yes, it was difficult. |
| 16 | Telefonum nerede? | Telefonumun sağ tarafında gizli kamera var. | Where is my phone? | There is a hidden camera on the right side of my phone. |
| 17 | Bugün yağmur var mı? | Evet yarın sağanak bekleniyor. | Is it going to rain today? | Yes, heavy rain is expected tomorrow. |
| 18 | Kapıyı kilitledin mi? | Evet zilyetliğin açık olması iyi olur. | Did you lock the door? | Yes, it would be good for possession to be open. |
| 19 | Proje bitti mi? | Hayır inşaat devam ediyor. | Is the project finished? | No, the construction is continuing. |
| 20 | Kargo geldi mi? | Evet dağıtım şirketi dağıtımdan sorumlu. | Has the package arrived? | Yes, the distribution company is responsible for distribution. |

| Qwen_Fine_tuning_responses.csv | | | | |
| --- | --- | --- | --- | --- |
| **ID** | **Questions**  **(Turkish Language)** | **Answer**  **(Turkish Language)** | **Question (Translation)** | **Answer (Translation)** |
| 1 | Kahvaltı hazır mı? | Yani, bu muhteşem! 15. yılım için daha iyi olacak Bağlam: Duygusal deney | Is breakfast ready? | So, this is magnificent! It will be better for my 15th year Context: Emotional experience |
| 2 | Bugün dışarı çıkacak mıyız? | Yemek mi? İşte ama bazı şerbiye döndüğün bana nerede olduğunu bilmiyorum. Her | Are we going out today? | Food? Here but some sherbiye when you returned I do not know where it is. Every |
| 3 | Film izleyelim mi? | Yıllardır kendimi biraz daha fazla yola çıkarmıştım. Bu çok iyiydi. Korku! Bahse gir | Shall we watch a movie? | For years I had set myself out on the road a little more. This was very good. Fear! I bet |
| 4 | Hava bugün soğuk mu? | Çok hayır ama hiç uzak değil mi? Şey. Onu yapmak istemedim ama onu yapabileceğimi sanırım. Ba | Is it cold today? | Very no but is it not far at all? Thing. I did not want to do it but I think I could do it. Ba |
| 5 | Ders başladı mı? | Yani, ama en iyileriyle ilgili konuları yapmalısın. Bence. İyi bir arkadaşım var ve bu | Has the class started? | So, but you should do the topics related to the best ones. I think. I have a good friend and this |
| 6 | Eve geç mi kalacağız? | Evinin 2015'a devam edildiğinde. Sadece daha sonra birlikte oynamaya devam etm | Will we be late getting home? | When your house was continued to 2015. Just later continue to play together |
| 7 | Toplantı iptal mi? | Yemek izlemeye gidiyorum ve çok iyi çalışıyorum Bağlam: Duygusal deneyimleravediye | Is the meeting cancelled? | I am going to watch food and I am working very well Context: Emotional experiencessgift |
| 8 | Çay ister misin? | Yapmadı. Yani daha fazla çay alıyorum. Şok edici olmalıydı ama bu gerçekten çok iyi bir şey olabilir! | Would you like some tea? | He/She did not do it. So I am getting more tea. It should have been shocking but this can really be a very good thing! |
| 9 | İnternet çalışıyor mu? | Daha sonra. Bağlam: Duygusal deneyimlerzbollah ve ehteren dünyaya gidiyor. Kız arkadaşım | Is the internet working? | Later. Context: Emotional experienceszbollah and ehteren goes to the world. My girlfriend |
| 10 | Otobüs geldi mi? | Gören değil ama yeterince geçtim. Örkbende yaşıyorum. Yeni otobüste ne yaptım? Bir h | Has the bus arrived? | Not seeing but I passed enough. I live in Örkben. What did I do on the new bus? A h |
| 11 | Saat kaç oldu? | Bir saat. Bir iş için özlüyorum ama bir gün önce çok sıkıştırdım ve iki hafta içinde yani | What time is it? | One hour. I long for a job but one day before I squeezed a lot and within two weeks so |
| 12 | Işıkları kapattın mı? | Harika yapacak kadar izin vermek için izini aldım! Bağlam: Duygusal deneyimler 10 classı | Did you turn off the lights? | I got permission to allow it enough to do great! Context: Emotional experiences 10 class |
| 13 | Kitabı bulabildin mi? | Yani. Onu alırsam, onu alırdım Yeni bir yolda dönerken geçirdim ama s | Were you able to find the book? | So. If I take it, I would take it I spent it while turning on a new road but s |
| 14 | Müzik açalım mı? | Onu gerçekten izlemek için biraz korkunç. Bu iyi. Bağlam: Duygusal deneyim 5 sınıf | Shall we play some music? | It is a bit scary to really watch it. This is good. Context: Emotional experience 5 class |
| 15 | Sınav zor muydu? | Çok uzun ve güzel bir zeviri aldı, bu yüzden benim için çok iyi geliyor. Ama her zaman bunu yaparken çok | Was the exam difficult? | It took a very long and beautiful translation, so it comes very good for me. But always while doing this very |
| 16 | Telefonum nerede? | 2.02.212345'dur, ancak bunu tekrar vermekten gerekir. Diyin | Where is my phone? | It is 2.02.212345, but it is necessary to give this again. Say |
| 17 | Bugün yağmur var mı? | Belki! Çok fazla bakman gerekiyordu. Bağlam: Duygusal deneyimler processıumesiyle il | Is it going to rain today? | Maybe! You had to look a lot. Context: Emotional experiences with its processıumesi |
| 18 | Kapıyı kilitledin mi? | Yavru, sadece bir kez ve ona sahip olmak için çakırım. Şok ediyor! Ne kadar zamand | Did you lock the door? | Puppy, just once and I strike to have it. It shocks! How much time |
| 19 | Proje bitti mi? | Yani, öyleyim! Örümek alımda birlikte başka bir şey yaptım. Yaptığımdan beri | Is the project finished? | So, I am like that! I did something else together in my knitting purchase. Since I did |
| 20 | Kargo geldi mi? | ildiyim. 18 yaşında ve 65 yaşındayım. Bağlam: Duygusal deneyim architekt | Has the package arrived? | I was. I am 18 years old and I am 65 years old. Context: Emotional experience architect |

| Qwen_RAG_responses.csv | | | | |
| --- | --- | --- | --- | --- |
| **ID** | **Questions**  **(Turkish Language)** | **Answer**  **(Turkish Language)** | **Question (Translation)** | **Answer (Translation)** |
| 1 | Kahvaltı hazır mı? | Öyle ama her zaman daha iyi olamayacağım. Her zaman bir şey yapmak için dolaşmaya devam ediyorlar | Is breakfast ready? | That is so but I will not always be able to be better. They always continue to wander around to do something |
| 2 | Bugün dışarı çıkacak mıyız? | Bu kulağa çok iğrenç geliyor. Birisi çöp yapmaya karar verdiler ve bunu yaptığım için | Are we going out today? | This sounds very disgusting. Someone decided to make trash and because I did this |
| 3 | Film izleyelim mi? | Evet. 20 yıl sonra biraz daha ilginç oluyor ama bu o kadar çok şeyi hatırlıyor ki Görüntü | Shall we watch a movie? | Yes. After 20 years it becomes a little more interesting but it remembers so many things Image |
| 4 | Hava bugün soğuk mu? | O harika! Ama 10 saatten önce karanlık ve soğuk bir yere gittik. Sanırım hepsi yar | Is it cold today? | That is great! But before 10 hours we went to a dark and cold place. I think all of them yar |
| 5 | Ders başladı mı? | Yani, çok çalıştım ama yine de oyun oynardım Ondan sonra şahsen yapabileceğimi sanmıyorum | Has the class started? | So, I worked a lot but I would still play games After that I do not think I could do it personally |
| 6 | Eve geç mi kalacağız? | Ben de yapacağım. Her zaman benim için bir yeri bekliyorum. Ondan sonra kendimi çok kötü hissediyorum | Will we be late getting home? | I will do it too. I am always waiting for a place for me. After that I feel very bad |
| 7 | Toplantı iptal mi? | Evet, benim için 25$'lık bir eşiçiyi getirdi. Kızım onu aldatıp içeri | Is the meeting cancelled? | Yes, he/she brought a 25$ threshold for me. My daughter deceived him/her and inside |
| 8 | Çay ister misin? | Evcil yarın mı? Bir şansın var Yeni bir şey yapmayı seviyorum. En iyisi! Bu kon | Would you like some tea? | Pet tomorrow? You have a chance I like to do something new. The best! This sub |
| 9 | İnternet çalışıyor mu? | İyi yapar. Ama onları seviyorum. Konuyla ilgili sorular buldum Daha önce seviyor mus | Is the internet working? | He/She does well. But I like them. I found questions related to the subject Do you like before |
| 10 | Otobüs geldi mi? | Yıllık otobüste öyle oldu. Oh hayır bu harika. Bu kadar uzun bir yol. Umarım kendini daha | Has the bus arrived? | It happened like that on the annual bus. Oh no this is great. Such a long road. I hope you yourself more |
| 11 | Saat kaç oldu? | 20 Yapınca bir şey için çalışıyorum ama buna daha fazla destek vereceğimi sanmıyorum. Örnek | What time is it? | When doing 20 I am working for something but I do not think I will give more support to this. Example |
| 12 | Işıkları kapattın mı? | Yarım gün 126 doları ödemeydi. Sonunda iğrendiğimi biliyordum. Sor | Did you turn off the lights? | Half a day was the payment of 126 dollars. In the end I knew that I was disgusted. Que |
| 13 | Kitabı bulabildin mi? | Yaptı ve çok minnettarım! Bu konuda çok stresliydim ama sonunda buna değer oldu. Ondan sonra on | Were you able to find the book? | He/She did it and I am very grateful! I was very stressed about this but in the end it was worth it. After that on |
| 14 | Müzik açalım mı? | Ama yetişik olarak ona biraz daha dolaşmam gerekecek. Oğlum benim için işe geçmedi ama | Shall we play some music? | But as an adult I will need to walk around him/her a bit more. My son did not go to work for me but |
| 15 | Sınav zor muydu? | O kadar iyi değil. Daha sonra bir sürü sorularla konuşmaya çalıştım ama sadece her şeyi alamadım. Ok | Was the exam difficult? | Not that good. Later I tried to talk with a lot of questions but I just could not get everything. Ok |
| 16 | Telefonum nerede? | Hahaha. Gerçekten kafamı çaldım ve bir evin içine koydular. Biraz tuhaf bir şeyi | Where is my phone? | Hahaha. I really stole my head and they put it inside a house. A bit of a strange thing |
| 17 | Bugün yağmur var mı? | Yeni bir palisade bulacağım. Yani onu seviyorum! Notlarla ilgili çok önemli bir soruydu | Is it going to rain today? | I will find a new palisade. So I love it! It was a very important question related to notes |
| 18 | Kapıyı kilitledin mi? | Evet ama onu yaralandırdım Örnek verilen Daha fazla söylemek istemiyorum İşte | Did you lock the door? | Yes but I injured him/her Given as an example I do not want to say more Here |
| 19 | Proje bitti mi? | Yapmıştım ama onu çok iyi tanıyor ve yapamazdım. Kredi kartımı yedin ve bana her şeyi özlü | Is the project finished? | I had done it but he/she knows him/her very well and I could not do it. You ate my credit card and miss everything for me |
| 20 | Kargo geldi mi? | Hiç değil, onun başına gelip geçtin! Kötü bir şey yapmış olmamalı. Bir köpek. Umar | Has the package arrived? | Not at all, you came and passed over him/her! He/She must not have done a bad thing. A dog. Hope |
